# Genome‐wide association study of facial emotion recognition in children and association with polygenic risk for mental health disorders

**DOI:** 10.1002/ajmg.b.32558

**Published:** 2017-06-13

**Authors:** Jonathan R.I. Coleman, Kathryn J. Lester, Robert Keers, Marcus R. Munafò, Gerome Breen, Thalia C. Eley

**Affiliations:** ^1^ King's College London, Institute of Psychiatry, Psychology and Neuroscience, MRC Social Genetic and Developmental Psychiatry (SGDP) Centre London UK; ^2^ National Institute for Health Research Biomedical Research Centre South London and Maudsley National Health Service Trust London UK; ^3^ School of Psychology University of Sussex Brighton UK; ^4^ School of Biological and Chemical Sciences Queen Mary University of London London UK; ^5^ MRC Integrative Epidemiology Unit University of Bristol Bristol UK; ^6^ UK Centre for Tobacco and Alcohol Studies, School of Experimental Psychology University of Bristol Bristol UK

**Keywords:** ALSPAC, faces, genetics, genomics, polygenic risk scores

## Abstract

Emotion recognition is disrupted in many mental health disorders, which may reflect shared genetic aetiology between this trait and these disorders. We explored genetic influences on emotion recognition and the relationship between these influences and mental health phenotypes. Eight‐year‐old participants (*n* = 4,097) from the Avon Longitudinal Study of Parents and Children (ALSPAC) completed the Diagnostic Analysis of Non‐Verbal Accuracy (DANVA) faces test. Genome‐wide genotype data was available from the Illumina HumanHap550 Quad microarray. Genome‐wide association studies were performed to assess associations with recognition of individual emotions and emotion in general. Exploratory polygenic risk scoring was performed using published genomic data for schizophrenia, bipolar disorder, depression, autism spectrum disorder, anorexia, and anxiety disorders. No individual genetic variants were identified at conventional levels of significance in any analysis although several loci were associated at a level suggestive of significance. SNP‐chip heritability analyses did not identify a heritable component of variance for any phenotype. Polygenic scores were not associated with any phenotype. The effect sizes of variants influencing emotion recognition are likely to be small. Previous studies of emotion identification have yielded non‐zero estimates of SNP‐heritability. This discrepancy is likely due to differences in the measurement and analysis of the phenotype.

## 1 INTRODUCTION

Emotion permeates everyday social interaction and represents a central component of human (and other primate) societies (Brothers, 1990; Ekman, 2007; Salovey & Mayer, 1989). The importance of emotion recognition as a social skill is further indicated by the disruption of emotional recognition in a number of psychiatric disorders, including autism (Harms, Martin, & Wallace, 2010), schizophrenia (Kohler, Walker, Martin, Healey, & Moberg, 2010), depression (Bourke, Douglas, & Porter, 2010; Kohler, Hoffman, Eastman, Healey, & Moberg, 2011), bipolar disorder (Kohler et al., 2011), and anxiety (Demenescu, Kortekaas, den Boer, & Aleman, 2010). The ability to infer emotions displayed by others could represent an important influence on the individual that shapes their behavior within a society, as well as their mental health (Lopes, Salovey, Côté, Beers, & Petty, 2005).

To date, a few studies have examined the role of genes in facial emotion recognition, implicating variants in the oxytocin receptor gene *OXTR* with the identification of emotions (Skuse et al., 2014), and the catechol‐O‐methyl transferase gene *COMT* with response time to emotional faces (Weiss et al., 2007). Investigations of the effect of variation in the promoter region of the serotonin transported gene (5HTTLPR) found no differences between emotions in terms of identification but found some evidence of an association with the intensity of emotion at which recognition occurred (Antypa, Cerit, Kruijt, Verhoeven, & Van der Does, 2011). There has also been a considerable literature linking 5HTTLPR to amygdala activation, including in response to emotional faces (Canli & Lesch, 2007). However, these studies use a candidate gene approach, which is limited by focusing on a few regions of assumed relevance, and usually relies on small sample sizes that are underpowered to detect likely effect sizes (Dick et al., 2015; Ioannidis, 2003). Previous work in the Philadelphia Neurodevelopmental Cohort has investigated emotion identification (among other phenotypes) genome‐wide, focusing on estimation of heritability (identifying a common‐variant heritability of 36%) and polygenic risk relationships with schizophrenia (Germine et al., 2016; Robinson et al., 2015).

Alternative approaches have also provided insights into the genetics of emotion recognition. Epidemiological observation of emotion recognition deficits in X‐linked disorders including Turner's syndrome and fragile X disorder argues for a role of variants on the X chromosome (Bouras, Turk, & Cornish, 1998; Lawrence, Kuntsi, Coleman, Campbell, & Skuse, 2003; Skuse, 2006). A family‐based quantitative genetic study of individuals with schizophrenia has estimated the heritability (additive genetic component of variance) of emotion recognition in faces at approximately 35% (Greenwood et al., 2007). In contrast, research on typically developing twins in childhood identified a large heritable component of general emotion recognition in faces that was shared across different emotions (75%), although no emotion‐specific components were identified (Lau et al., 2009).

Facial emotion recognition deficits have been reported in individuals suffering from schizophrenia, bipolar disorder, depression, autism spectrum disorder, and mixed evidence exists for similar deficits in anorexia and anxiety disorders (Bourke et al., 2010; Collin, Bindra, Raju, Gillberg, & Minnis, 2013; Demenescu et al., 2010; Harms et al., 2010; Kohler et al., 2010; Kohler et al., 2011). Large GWAS of these disorders exist, and may predict variance in emotion recognition in the present cohort (Otowa et al., 2016; Ripke et al., 2013; Schizophrenia Working Group of the Psychiatric Genomics C, 2014; Sklar et al., 2011). Increased understanding of intact emotion recognition may aid in understanding the nature and importance of emotion recognition deficits in these disorders. Accordingly, we investigated the association between polygenic risk scores derived from GWAS of these disorders and facial emotion recognition phenotypes to assess whether genetic correlations mirror reported comorbidities.

In this study, we performed GWAS of non‐verbal emotion recognition in children from the Avon Longitudinal Study of Parents and Children (ALSPAC). We then used polygenic risk score analysis to predict individual differences in emotion recognition within this cohort, using polygenic risk scores from studies of psychiatric disorders in which emotion recognition is impaired.

## 2 METHOD

### 2.1 Participants

Participants were drawn from ALSPAC, which has been described in detail elsewhere (Boyd et al., 2012). In brief, approximately 15,000 pregnant women resident in Avon, UK with expected dates of delivery between April 1st, 1991 and December 31st, 1992 were recruited into a prospective birth cohort to study the effects of environmental and genetic influences on health and development. Additional information on the ALSPAC cohort is available on the study website, through a fully searchable data dictionary (http://www.bris.ac.uk/alspac/researchers/data-access/data-dictionary/).

### 2.2 Measurement of facial emotion recognition

A total of 7,297 of the child participants in ALSPAC underwent the Diagnostic Analysis of Non‐Verbal Accuracy test (DANVA) as part of the “Focus at 8” assessment, performed when the participants were approximately 8 years old (Nowicki & Carton, 1993). The “Focus at 8” assessments comprised four sessions investigating psychometric and psychological characteristics, taking place across half a day. The DANVA was performed as part of the Activities session. Two tests from the DANVA were used, measuring the ability of participants to extract emotional information from the vocal tone (paralanguage) and face of actors. However, only data from the faces task was available to be analyzed in this study. During the task, the participant was shown 24 images of children displaying one of four emotions: happiness, sadness, anger, or fear. The image was displayed for 2 s, after which the participant was asked to identify the emotion verbally, and their response was recorded.

### 2.3 Genotyping and assessment of population stratification

The generation and quality control of genome‐wide genotype data are described on the ALSPAC website (http://www.bristol.ac.uk/media-library/sites/alspac/migrated/documents/gwas-data-generation.pdf). In brief, 9,912 of the child participants were genotyped on the Illumina HumanHap550 Quad microarray, and the resulting genotypes imputed to the HapMap2 release 22 for autosomes, and HapMap 3 release 2 for the X chromosome using MACH and Minimac, respectively (Howie, Fuchsberger, Stephens, Marchini, & Abecasis, 2012; Li, Willer, Ding, Scheet, & Abecasis, 2010). Following quality control to remove poorly‐performing variants and samples, 8,365 samples and 2,487,351 variants were available (500,527 genotyped). Additional filtering to that described in the ALSPAC documentation was applied to the imputed dataset to remove rare variants (minor allele frequency <0.01) and variants that had been poorly imputed (MACH Rsq < 0.3) before analysis.

Participants self‐reported white Western European ancestry. Principal components analysis of the genotyped data using EIGENSOFT yielded no principal components associated with the DANVA phenotypes at a level greater than chance. Given that the cohort comprised individuals of white Western European ancestry from a single region, no further correction for population stratification was made.

### 2.4 Analysis

Results from the DANVA were used to measure the participant's general ability at emotion recognition by calculating a proportion index (Rosenthal & Rubin, 1989). This measures a participant's performance across all 24 trials of the DANVA, scaled such that a score of 0.5 represents performance at chance (Eq. 1, where options is the number of choices, in this case 4). For example, if a participant correctly identified 21 of the 24 emotions, they would have a proportion index of 0.929. The proportion index was arcsine transformed and used as a phenotype in GWAS.

$$Proportion\;Index=\frac{\frac{Correct\;Responses}{Trials}\times \left(Options-1\right)}{1+\left(\frac{Correct\;Responses}{Trials}\times \left(Options-2\right)\right)}$$

Equation 1: Calculation of the proportion index.

In order to assess emotion‐specific genetic influences, unbiased hit rates were created for each emotion (Wagner, 1993). The unbiased hit rate represents the proportion of correct responses for a given emotion, weighted by the number of times the participant gave that response for the wrong face (Eq. 2). For example, if the participant identified all six happy faces correctly but wrongly identified two fearful faces as happy, they would have an unbiased hit rate for happy faces of 0.75.

$${Unbiased\;hit\;rate}_{i}=\frac{{Correct\;Responses}_{i}}{{Trials}_{i}}\times \frac{{Correct\;Responses}_{i}}{\left({Correct\;Responses}_{i}+{Incorrect\;Responses}_{i}\right)}$$

Equation 2: Calculation of the unbiased hit rate, where *i* is a given emotion.

Unbiased hit rates for each emotion were arcsine transformed and used as phenotypes in genome‐wide association studies (GWAS) performed in ProbAbel (http://www.genabel.org/), using MACH‐imputed dosage data (Aulchenko, Struchalin, & van Duijn, 2010; Li et al., 2010). Following each individual GWAS, variants were clumped in PLINK1.9 to identify linkage‐independent loci (Chang et al., 2015). Specifically, all variants were assigned to a locus if they were in linkage disequilibrium (*r* ^2^ > 0.25) with a nearby (<250 kb) variant with a lower *p*‐value.

All GWAS analyses controlled for fixed effects of gender, age at assessment (in weeks), IQ at assessment, and whether the activities session was the first, second, third, or fourth performed (with first used as the reference condition; Supplementary Table S2). Further covariates were considered for inclusion, including summary results from each section of the Development and Wellbeing Assessment (DAWBA; (Goodman, Ford, Richards, Gatward, & Meltzer, 2000)), and components of the Family Adversity Index (Bowen, Heron, Waylen, & Wolke, 2005). However, these additional covariates were found to be uncorrelated with the DANVA phenotypes, and so were not included.

Performance on the Social and Communication Disorders Checklist (SCDC) was correlated with the phenotypic outcome. This questionnaire is a measure of flexibility and responsiveness to social interactions, and as such may involve the same cognitive processes as the DANVA (Skuse, James, Bishop, & Coppin, 1997). Analyses were run both with and without this covariate; these results were very similar, and so only analyses not including the questionnaire are presented. Recent analyses within ALSPAC investigated genetic variation associated with performance on this measure (St Pourcain et al., 2014). Accordingly, the results of the analysis of emotion recognition were contrasted with those found by St Pourcain et al. (2014).

Following the GWAS, exploratory secondary analyses were performed to investigate associations between higher‐order genetic elements and emotion recognition. Specifically, heritability estimation was performed using LD Score regression and the GREML option in GCTA (Bulik‐Sullivan et al., 2015; Yang, Lee, Goddard, & Visscher, 2011). These tools provide complementary methods to estimate the proportion of heritability explained by variants assessed in the study, using summary statistics and genotype data, respectively. A previous study of emotion recognition in children examined a score equivalent to the summed correct responses score used prior to conversion to the proportion index, yielding a heritability estimate of 36% (Robinson et al., 2015). In order to compare results directly between this study and that of Robinson et al. (2015), sensitivity analysis was run in GCTA using the summed correct responses score.

Results from external GWAS of schizophrenia (Schizophrenia Working Group of the Psychiatric Genomics C, 2014), bipolar disorder (Sklar et al., 2011), depression (Ripke et al., 2013), anxiety (Otowa et al., 2016), autism spectrum disorder (PGC.ASD.euro.all.25Mar2015.txt.gz, unpublished), and anorexia (pgc.an.13May2016.zip, unpublished; unpublished summary statistics available at https://www.med.unc.edu/pgc/results-and-downloads) were used for polygenic risk scoring. Associations between external traits and specific and general emotion recognition were assessed using the default high‐resolution polygenic risk scoring option implemented in PRSice (Euesden, Lewis, & O'Reilly, 2015; Purcell et al., 2009). Specifically, 10,000 risk scores were calculated from each of the external GWAS using an increasing threshold for the inclusion of single nucleotide polymorphisms (SNPS). Variants were included if their associated *p*‐value from the external GWAS fell beneath this threshold (*p* = 0.00005 to *p* = 0.5 in steps of 0.00005), and were weighted by their effect size in the external GWAS.

Within each of polygenic risk score analysis, an adjusted alpha threshold of *p* = 0.001 was used to correct for the assessment of multiple correlated risk scores (Euesden et al., 2015). Multiple analyses were performed across five DANVA phenotypes (recognition of happy, sad, angry and fearful faces, and overall recognition), using results from seven external GWAS studies (schizophrenia, bipolar disorder, major depressive disorder, autism, anorexia nervosa, and anxiety assessed as a case‐control and as a continuous phenotype). The number of effective tests resulting from these multiple analyses was determined using the Nyholt‐Šidák method (Nyholt, 2004). Specifically, the correlation matrix of the 35 optimal polygenic risk scores (Table 4) was calculated and spectral decomposition was used to determine the number of effective tests.

### 2.5 Ethics

Ethics approval for the study was obtained from the ALSPAC Ethics and Law Committee and the Local Research Ethics Committees. ALSPAC operates in accordance with the principles laid out in the Declaration of Helsinki (Mumford, 1999).

## 3 RESULTS

### 3.1 Data available for analysis and demographics

Of the 7,297 participants who completed the DANVA, 483 were excluded from the analysis because they provided responses for fewer than 23 of the 24 faces, 50 because their parent reported a diagnosis of autism spectrum disorder, and 118 because their IQ was less than 70. This resulted in 6,646 participants, of whom 4,097 also had genome‐wide genotyping data (2,487,351 variants available following imputation) and made up the analyzed cohort.

Demographic data for the cohort is displayed in Table 1. The cohort contained slightly more females (50.4%) and ranged from 7 to 10 years old (389–543 weeks, mean = 450 weeks, SD = 12 weeks). IQ ranged from 70 (lower IQs were removed) to 145 (mean = 106, SD = 15.7).

**Table 1:** Descriptive statistics for the analyzed cohort

Demographic data on the cohort	
*N*	4,097
Female gender (N [%])	2,066 [50.4]
Age in weeks (mean [SD])	450 [12.0]
IQ (mean [SD])	106 [15.7]
SCDC (mean [SD])	2.67 [3.39]

SCDC, sociocommunicative disorders checklist.

### 3.2 Performance of the DANVA faces task

Summed scores for the correct identification of all faces had only a modest internal consistency (Cronbach's alpha = 0.64), and summed scores for specific emotions had poor internal consistency (Cronbach's alpha ranges from 0.31–0.69), although this is limited by the small number of items per emotion (Supplementary Table S1). Measurements of skewness and kurtosis suggest that the arcsine transformation of the proportion index was necessary to improve the normality of this measure (Supplementary Table S1). Unbiased hit rates were acceptably normal before transformation, and their normality was largely unaffected by arcsine transformations (Supplementary Table S1). Transformed phenotypes were used to ensure consistency of treatment of all proportional phenotypes. Sensitivity analyses were performed on untransformed hit rates to assess the effect of this transformation.

Correct identification of faces differed by emotion. Participants were better at detecting happy faces compared to all other emotions, better at detecting sad faces than fearful or angry faces, and better at detecting fearful faces compared to angry faces (Table 2).

**Table 2:** Mean and 95% confidence intervals for correct responses for all emotions (out of 24) and individual emotions (out of 6), and *t*‐statistics and raw *p*‐values from paired *t*‐tests between individual emotions

	Correct responses		vs. Happy		vs. Sad		vs. Fearful	
Emotion	Mean	95%CI	*t*	*p*	*t*	*p*	*t*	*p*
All	19.4	19.3–19.5	–	–	–	–	–	–
Happy	5.71	5.69–5.73	–	–	–	–	–	–
Sad	5.30	5.27–5.33	−26.6	8.10 × 10^−144^	–	–	–	–
Fearful	4.47	4.43–4.51	−54.1	<10^−250^	−36.1	<10^−250^	–	–
Angry	3.94	3.90–3.98	−80.7	<10^−250^	−59.5	2.60 × 10^−248^	−20.6	5.89 × 10^−90^

All *p*‐values are significant at *α* = 0.0083 (Bonferroni correction for six tests).

### 3.3 GWAS results

**Figure 1:**
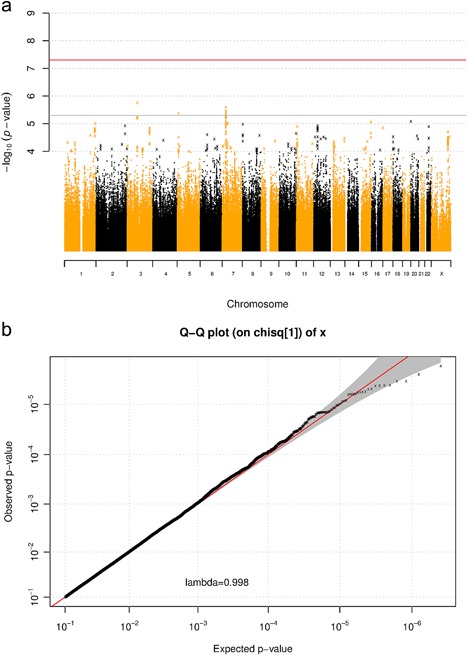
a) Manhattan plot showing associations between genetic variants and recognition of emotion faces in general. Base position of genetic variants on each chromosome are on the *x*‐axis, −log *p‐*value on the *y*‐axis. Genome‐wide significance (*p* = 5 × 10^−8^) is top line (red), and suggestive significance (*p* = 5 × 10^−6^) is bottom line (gray). b) Quantile–quantile plot shows observed associations between genetic variants and recognition of emotion in faces (*y*‐axis) do not deviate from those expected under the null distribution (*x*‐axis). Lambda median is a measure of genomic inflation. Lambda ≈ 1, indicating minimal inflation due to confounds. Color figure can be viewed at wileyonlinelibrary.com

No variants were identified at conventional levels of genome‐wide significance (*p* = 5 × 10^−8^) in any GWAS, but 10 loci reached suggestive levels of significance across the GWAS of individual emotions (Supplementary Figures S1–S4), with 5 of these loci attaining suggestive significance for general emotion recognition, along with an additional two variants (*p *< 5 × 10^−6^, Table 3, Figure 1).

**Table 3:** Linkage‐independent loci from the individual GWAS, and general emotion recognition GWAS with *p *< 5 × 10^−6^ (bold) in at least one analysis (gray)

Independent clumps associated with emotion recognition with *p *< 5 × 10^−6^												
			Happy		Sad		Fearful		Angry		General	
Sentinel SNP	A1	CHR	*Z*	*p*	*Z*	*p*	*Z*	*p*	*Z*	*p*	*Z*	*p*
rs9550616	A	13	**−4.69**	**2.88 × 10^−6^**	−1.76	0.0776	−1.27	0.205	−2.23	0.0260	−3.29	0.00102
rs3770081	G	2	−1.73	0.00845	**−4.77**	**1.94 × 10^−6^**	−1.08	0.278	−4.33	1.55 × 10^−5^	−3.69	2.33 × 10^−4^
rs12705054	A	7	−1.53	0.126	**−4.65**	**3.45 × 10^−6^**	−1.58	0.114	−3.11	0.00188	−3.72	1.98 × 10^−4^
rs2080301	A		−3.76	1.70 × 10^−4^	−3.90	9.88 × 10^−5^	−3.32	8.94 × 10^−4^	−4.10	8.16 × 10^−6^	**−4.57**	**4.96 × 10^−6^**
rs17604090	A	7	2.77	0.00556	**4.60**	**4.30 × 10^−6^**	2.92	0.00354	4.47	4.27 × 10^−5^	**4.71**	**2.56 × 10^−6^**
rs10248839	C		3.12	0.00182	4.16	3.25 × 10^−5^	2.83	0.00468	**4.61**	**4.10 × 10^−6^**	**4.61**	**4.18 × 10^−6^**
rs1146849	A	13	−1.20	0.230	**−4.60**	**4.31 × 10^−6^**	−1.51	0.130	−4.03	5.61 × 10^−5^	−3.30	9.59 × 10^−4^
rs654861	A	6	2.66	0.00776	1.78	0.0754	**4.86**	**1.19 × 10^−6^**	2.11	0.0351	3.96	7.72 × 10^−5^
rs2304503	A	3	−2.72	0.00655	−0.590	0.555	**−4.64**	**3.59 × 10^−6^**	−0.382	0.702	−2.50	0.0123
rs10499395	G	7	2.91	0.00368	2.01	0.0441	1.41	0.157	**4.76**	**2.03 × 10^−6^**	3.66	2.59 × 10^−4^
rs4930838	A	12	0.551	0.582	2.29	0.0220	0.798	0.425	**4.65**	**3.42 × 10^−6^**	2.62	0.00872
rs683257	A	6	−2.39	0.0167	−2.98	0.00288	−2.00	0.0461	**−4.58**	**4.72 × 10^−6^**	−3.68	2.33 × 10^−4^
rs17016200	G	3	2.50	0.0124	4.33	1.49 × 10^−5^	3.67	2.49 × 10^−4^	3.84	1.26 × 10^−4^	**4.79**	**1.74 × 10^−6^**
rs1423494	C	5	−3.36	7.81 × 10^−4^	−3.54	4.09 × 10^−4^	−3.46	5.42 × 10^−4^	−3.58	3.50 × 10^−4^	**−4.61**	**4.23 × 10^−6^**

Each locus is represented by a sentinel SNP, that with the lowest *p*‐value in the locus. One locus on chromosome 7 showed different sentinel SNPs across different analyses, so is represented by three SNPs. Positive direction of effect means better recognition of emotion with each effect allele (A1). Locus information is provided in Supplementary Table S3.

Sensitivity analyses of the specific emotion GWAS using untransformed hit rates produced results that did not qualitatively differ from those using the transformed phenotypes (Supplementary Table S4). All variants with *p* < 5 × 10^−6^ in a specific analysis in the main GWAS had *p* < 5 × 10^‐5^ in the relevant sensitivity analysis (Supplementary Table S5).

Post‐hoc power analyses were conducted using Genetic Power Calculator (Purcell, Cherny, & Sham, 2003). The cohort of 4,097 participants is adequate to detect a variant capturing 0.97% of variance at 80% power. For comparison, the most variance captured by any of the top SNPs in the analysis of individual emotions was 0.047% (rs3770081, sad faces, Table 3); a total of 84,320 participants would be required to capture this level of variance at 80% power.

### 3.4 Polygenic risk scoring

Spectral decomposition of the optimal scores from the 35 polygenic risk scoring analyses suggested 33.22 effective tests were performed, resulting in an adjusted alpha threshold of 3.01 × 10^−5^ (≈0.001/33.22) Figure 1.

Polygenic risk scores from the most recent GWAS of schizophrenia, bipolar disorder, depression and autism spectrum disorder from the Psychiatric Genomics Consortium showed no predictive effects in the sample (Table 4). Three PRS passed correction for the 10,000 non‐independent tests involved in a single PRSice analysis (*p *< 0.001): autism predicting fear recognition (*p* = 7.32 × 10^−4^), anxiety (as a case‐control phenotype) predicting recognition of happy faces (*p* = 6.72 × 10^−4^) and anxiety (as a factor score) predicting angry faces (*p* = 6.62 × 10^−4^; (Euesden et al., 2015)). However, none was significant when taking into account the testing of multiple phenotypes (all *p *> 3.01 × 10^−5^; (Nyholt, 2004)). Plots of PRS associations across common thresholds are provided for each analysis in the Supplementary Material (Supplementary Figures S5–S11).

**Table 4:** Variance explained and *p* values for the best polygenic risk scores from the mental health GWAS, predicting recognition of emotion

Emotion predicted	Best threshold	*p*‐value at best threshold	Variance explained (R^2^)
Schizophrenia risk predicting emotions			
Happy	0.123	0.222	0.000358
Sad	0.00565	0.0317	0.00109
Angry	0.0295	0.175	0.000433
Fearful	0.0464	0.456	0.000131
Proportion index	0.0176	0.163	0.000452
Bipolar disorder risk predicting emotions			
Happy	0.06525	0.0494	0.000928
Sad	0.00115	0.322	0.000232
Angry	0.00820	0.125	0.000553
Fearful	0.1083	0.0941	0.000661
Proportion index	0.1083	0.0280	0.00112
Major depressive disorder risk predicting emotions			
Happy	0.1989	0.0488	0.000933
Sad	0.0101	0.122	0.000564
Angry	0.00455	0.0303	0.00110
Fearful	0.00125	0.137	0.000520
Proportion index	0.2237	0.0381	0.00100
Autism spectrum disorder risk predicting emotions			
Happy	0.00345	0.0610	0.000844
Sad	6.00 × 10^−4^	0.0116	0.00150
Angry	7.00 × 10^−4^	0.110	0.000600
Fearful	0.01365	7.32 × 10^−4^	0.00268
Proportion index	1.00 × 10^−4^	0.0204	0.00125
Anorexia risk predicting emotions			
Happy	0.00350	0.0737	0.000769
Sad	8.50 × 10^−4^	0.137	0.000523
Angry	0.2103	0.0799	0.000722
Fearful	0.00555	0.163	0.000459
Proportion index	8.50 × 10^−4^	0.0581	0.000835
Anxiety (case‐control) risk predicting emotions			
Happy	0.03115	6.72 × 10^−4^	0.00278
Sad	5.00 × 10^−5^	0.216	0.000362
Angry	0.0382	0.1095	0.000603
Fearful	4.00 × 10^−4^	0.0818	0.000714
Proportion index	0.0382	0.0386	0.000994
Anxiety (factor score) risk predicting emotions			
Happy	0.00370	0.150	0.000498
Sad	5.50 × 10^−4^	0.0369	0.00103
Angry	2.50 × 10^−4^	6.62 × 10^−4^	0.00272
Fearful	0.00370	0.182	0.000420
Proportion index	2.50 × 10^−4^	0.0228	0.00120

Three associations passes the recommended *p* = 0.001 for a single analysis, but not the adjusted threshold (*p* = 3.01 × 10^−5^) for the 33.22 effective tests performed (Euesden et al., 2015; Nyholt, 2004).

### 3.5 Secondary analyses

Estimation of heritability was attempted from each of the individual emotion GWAS and from the general emotion recognition GWAS using LD Score regression (Bulik‐Sullivan et al., 2015). No analysis yielded an estimate significantly different from zero—the largest estimate was for sad face recognition: *h* ^2^ = 0.0077 (95CI: −0.209–0.224). Similar results were obtained from equivalent analyses in GCTA (Yang et al., 2011). Power analyses suggest that the sample of 4,097 has 80% power to detect heritability >0.22, sufficient to capture previously reported estimates of heritability (Greenwood et al., 2007; Robinson et al., 2015; Visscher et al., 2014). Sensitivity analyses examining the summed correct responses across all emotions in GCTA did not yield a significant estimate of heritability.

## 4 DISCUSSION

We performed GWAS of facial emotion recognition in a population cohort of children. In concordance with psychological and behavioral genomic studies to date, no variants of large effect were detected in the sample. Although no variants were present at genome‐wide significance, 12 independent loci were identified at a suggestive level of significance across the five analyses performed. The region on chromosome 7p15.1 is of most interest, as it passed the suggestive threshold in the analysis of both sad and angry faces, and in the analysis of general emotion recognition. The locus lies across an unstudied long non‐coding RNA (LOC646762) and near the genes *CHN2*, *PRR15*, and *WIPF3*. Of these genes, chimerin 2 (*CHN2*) is the most interesting candidate. It encodes beta‐chimerin, a rho‐GTPase activating protein involved in the phospholipase C cell signaling pathway, proposed to have a regulatory function in the central nervous system (Yang & Kazanietz, 2007). *CHN2* is highly expressed in the brain and has previously been implicated in schizophrenia, although neither this gene nor the locus of interest was present in the largest GWAS of schizophrenia to date (Hashimoto et al., 2005; Schizophrenia Working Group of the Psychiatric Genomics C, 2014). However, it should be noted that biological interest has proved an unreliable indicator of true association in GWAS to date (Collins & Sullivan, 2013). Furthermore, although the region discussed passes the threshold for suggestive significance, it is not genome‐wide significant, and as such could be accounted for by random chance alone.

The sample size studied is relatively large for a psychological study; however, it is modest for a GWAS. As such, analyses only had statistical power to detect moderate effect sizes. Studies of psychological and behavioral traits to date suggest emotion recognition is likely to be highly polygenic, with multiple variants each contributing only a small effect (Munafo & Flint, 2014). Our results are consistent with such a model, and place an upper bound on the effect sizes to be expected from any larger study or meta‐analysis. However, these results are also consistent with the null hypothesis of no genetic effects. The weight of evidence from the literature supports the hypothesized polygenicity of emotion recognition (Germine et al., 2016; Robinson et al., 2015). The results presented herein do not provide additional support, yet polygenicity remains more likely than the absence of a common, additive genetic component to emotion recognition.

Estimation of heritability was performed using common SNP data, which captures only a proportion of total heritability (Wray et al., 2013). No estimate of heritability could be obtained from the analyses presented. Previous attempts to use this method for behavioral phenotypes have reported similarly non‐significant or low estimates of heritability, which may result from differences in analytical approach and sample characteristics (Pappa et al., 2015; St Pourcain et al., 2015; Trzaskowski, Dale, & Plomin, 2013). The null estimate of heritability does not appear to be due to sample size, as power calculations suggest the cohort was powered to detect the 36% SNP heritability previously reported (Robinson et al., 2015). Although this study and that of Robinson et al. (2015) assessed similarly sized cohorts of juvenile participants of European ancestry (*N* = 4,097 and *N* = 3,661, respectively), there are a number of methodological differences that may underlie the differing results. First, there are some demographic differences—Robinson et al. (2015) studied an American cohort with ages ranging 8–21, whereas the ALSPAC cohort is British and younger (ages ranged 7–10). The approach to measuring emotion recognition also differed. Robinson et al. (2015) used the Penn Computerized Neurocognitive Battery (CNB) Emotion Identification test (Gur et al., 2012). This measure assesses the same four emotions as the DANVA (but also includes a neutral face condition) and its output is the sum of all correct responses. As such, it is equivalent to the summed correct answers from the DANVA before calculation of the proportion index. The use of a proportion index in this study cannot account for the discrepancy in heritability estimates, because null results were obtained using the summed correct answers from the DANVA as a phenotype in GCTA. The reported internal consistency of the Penn CNB Emotion Identification test (Cronbach's alpha = 0.75) was superior to that achieved by the DANVA in this study (0.64), suggesting that the lower reliability of the DANVA phenotype might account for the observed discrepancy.

Previous studies of emotion recognition by Greenwood et al. (2007) and Lau et al. (2009) differed considerably from the current study in their sample composition and analytical approach. The estimate of heritability from Greenwood et al. (2007) is derived from the Penn CNB Emotion Identification test described above (Kohler et al., 2003). In addition, the participants differ considerably—Greenwood et al. (2007) studied families of adults with schizophrenia, whereas the data analyzed herein were drawn from a population cohort of children prior to puberty (after which there is evidence for an increase in facial emotion recognition ability; Thomas, De Bellis, Graham, & LaBar, 2007).

The cohort studied by Lau et al. (2009) was more similar to that investigated in this study, being comprised of 10 year old twins, albeit selected for high levels of parent‐reported anxiety (Lau et al., 2009). However, although accuracy of emotion recognition was measured, the experiment used a face morphing from a neutral condition to an emotional condition, rather than static images. The analysis of heritability also differs. The reported figure of 75% is derived from a latent factor analysis model in which a single genetic factor influences emotion recognition in all faces. Estimates of heritability from individual emotions (both from univariate analyses and modeled as emotion‐specific effects in the latent class analysis) were not significantly different from zero (Lau et al., 2009). This suggests that there may be a general genetic component of emotion recognition that was not captured in the analyses presented in this paper. The differences between the study presented herein and previous studies reporting heritability estimates are such that it is difficult to make inferences about the accuracy or generalizability of previously reported estimates.

The conclusions about the relative power of these analyses are supported by the results from previous analyses of socio‐communicative ability. Neither the analyses of emotion recognition nor previous analyses of the Social and Communication Disorders Checklist identified individual variants at genome‐wide significance, which suggests low power to detect small effect sizes (St Pourcain et al., 2014). However, an estimate of heritability of 24% was obtained for socio‐communicative ability using GCTA (St Pourcain et al., 2014). The heritability of outcome from the Social and Communication Disorders Checklist in children (<11 years old) has previously been estimated at 74–78%, using twin‐based methods (Scourfield, Martin, Lewis, & McGuffin, 1999; Skuse, Mandy, & Scourfield, 2005). The estimate of heritability from common variants is only a third of that estimated from twin methods, further demonstrating low heritability estimates from common variants in behavioral phenotypes.

Polygenic risk scoring was unable to identify significant predictors. Although power estimation is possible in polygenic risk scoring, the number of variables involved makes accurate estimation difficult without prior knowledge of the relationship between the phenotypes under study (Dudbridge, 2013; Palla & Dudbridge, 2015).

Emotion recognition is a complex phenotype requiring attention to cues in multiple areas of the face, which change subtly in real‐time (Bassili, 1979). It is likely to involve an intricate network of neural interactions (Vuilleumier & Pourtois, 2007). The faces component of the DANVA (as used in the ALSPAC study) is a comparatively simple forced‐choice test between static pictures of the four emotions studied. As such, the DANVA can only provide a limited measure of facial emotion recognition. Furthermore, because the DANVA does not include a neutral face condition, we were unable to control for general face recognition ability in this analysis. As such, we cannot separate associations between genetic variants and face recognition from those with emotion recognition. Future studies could achieve this separation by meta‐analyzing GWAS of emotion recognition in faces and in voices. At least a proportion of the variants associated with emotion recognition in faces would be expected to be associated with recognition of emotion in verbal tone (such as in the paralanguage component of the DANVA, which was not available during this study).

We performed GWAS of non‐verbal emotion recognition in a population cohort of children. Although no variants were identified at genome‐wide significance, the modest power of the sample suggests an upper threshold on the expected effect sizes of individual variants on this phenotype. Similarly, we were unable to obtain an estimate of heritability for any emotion recognition phenotype, despite power to detect true SNP heritabilities of 22%, lower than the reported SNP heritability of 36%. Emotion recognition is a complex phenotype, and its measurement is a simplification by necessity. Insights into the genetics of emotion recognition could inform our understanding of psychiatric disorders and of the basis by which individuals interact with their environment. Accordingly, a challenge for future research will be to combine sensitive measures of emotion recognition with the sample sizes required to capture the small effect sizes of variants suggested by the behavioral genetic literature.

## Supporting information

Additional Supporting Information may be found online in the supporting information tab for this article.

**Table S1**. Distributional statistics for the phenotypes in the study, including measurements of internal consistency of the basic summed scores resulting from the DANVA.
**Table S2**. Associations between unbiased hit rates of emotion recognition for each emotion and covariates. Positive betas indicate more accurate emotion recognition.
**Table S3**. Locus information for top clumps associated with emotion recognition in GWAS. Base positions are hg19.
**Table S4**. Linkage‐independent loci from sensitivity analyses, performing individual emotion GWAS without arcsine transformation of the phenotype. Loci with *p *< 5 × 10^−6^ in bold, sentinel SNPs from each analysis shaded gray. Each locus is represented by a sentinel SNP, that with the lowest *p*‐value in the locus. One locus on chromosome 7 showed different sentinel SNPs across different analyses, so is represented by three SNPs. Positive direction of effect means better recognition of emotion with each effect allele (A1).
**Table S5**. Results from the sensitivity analysis without arcsine transformation for the sentinel SNPs listed in Table 3. SNPs with *p* < 5 × 10^−6^ are shown in bold. Sentinel SNPs from each of the four specific emotion analyses are shown in gray. b: Quantile‐quantile plot shows observed associations between genetic variants and recognition of happy faces (*y*‐axis) do not deviate from those expected under the null distribution (*x*‐axis). Lambda median is a measure of genomic inflation. Lambda ≈ 1, indicating minimal inflation due to confounds.
**Figure S1**.Manhattan plot showing associations between genetic variants and recognition of happy faces. Base position of genetic variants on each chromosome are on the *x*‐axis, ‐log *p*‐value on the *y*‐axis. Genome‐wide significance (*p *= 5 × 10^−8^) is top line (red), and suggestive significance (*p *= 5 × 10^−6^) is bottom line (gray).
**Figure S2**. Manhattan plot showing associations between genetic variants and recognition of sad faces. b: QQ plot showing no deviation from the null expectation for genetic variants and recognition of sad faces.
**Figure S3**. Manhattan plot showing associations between genetic variants and recognition of fearful faces. b: QQ plot showing no deviation from the null expectation for genetic variants and recognition of fearful faces.
**Figure S4**. Manhattan plot showing associations between genetic variants and recognition of angry faces. b: QQ plot showing no deviation from the null expectation for genetic variants and recognition of angry faces.
**Figure S5**. Association of Schizophrenia PRS across seven thresholds (Pt = 0.01, 0.05, 0.1, 0.2, 0.3, 0.4, 0.5) and the optimal threshold. a: Schizophrenia PRS association with response to happy faces. b: Schizophrenia PRS association with response to sad faces. c: Schizophrenia PRS association with response to angry faces. d: Schizophrenia PRS association with response to fearful faces. e: Schizophrenia PRS association with response to facial emotion as a proportion index.
**Figure S6**. Association of Bipolar Disorder PRS across seven thresholds (Pt = 0.01, 0.05, 0.1, 0.2, 0.3, 0.4, 0.5) and the optimal threshold. a: Bipolar Disorder PRS association with response to happy faces. b: Bipolar Disorder PRS association with response to sad faces. c: Bipolar Disorder PRS association with response to angry faces. d: Bipolar Disorder PRS association with response to fearful faces. e: Bipolar Disorder PRS association with response to facial emotion as a proportion index.
**Figure S7**. Association of Major Depressive Disorder PRS across seven thresholds (Pt = 0.01, 0.05, 0.1, 0.2, 0.3, 0.4, 0.5) and the optimal threshold. a: Major Depressive Disorder PRS association with response to happy faces. b: Major Depressive Disorder PRS association with response to sad faces. c: Major Depressive Disorder PRS association with response to angry faces. d: Major Depressive Disorder PRS association with response to fearful faces. e: Major Depressive Disorder PRS association with response to facial emotion as a proportion index.
**Figure S8**. Association of Autism Spectrum Disorder PRS across seven thresholds (Pt = 0.01, 0.05, 0.1, 0.2, 0.3, 0.4, 0.5) and the optimal threshold. a: Autism Spectrum Disorder PRS association with response to happy faces. b: Autism Spectrum Disorder PRS association with response to sad faces. c: Autism Spectrum Disorder PRS association with response to angry faces. d: Autism Spectrum Disorder PRS association with response to fearful faces. e: Autism Spectrum Disorder PRS association with response to facial emotion as a proportion index.
**Figure S9**. Association of Anorexia Nervosa PRS across seven thresholds (Pt = 0.01, 0.05, 0.1, 0.2, 0.3, 0.4, 0.5) and the optimal threshold. a: Anorexia Nervosa PRS association with response to happy faces. b: Anorexia Nervosa PRS association with response to sad faces. c: Anorexia Nervosa PRS association with response to angry faces. d: Anorexia Nervosa PRS association with response to fearful faces. e: Anorexia Nervosa PRS association with response to facial emotion as a proportion index.
**Figure S10**. Association of Anxiety (Case‐Control) PRS across seven thresholds (Pt = 0.01, 0.05, 0.1, 0.2, 0.3, 0.4, 0.5) and the optimal threshold. a: Anxiety (Case‐Control) PRS association with response to happy faces. b: Anxiety (Case‐Control) PRS association with response to sad faces. c: Anxiety (Case‐Control) PRS association with response to angry faces. d: Anxiety (Case‐Control) PRS association with response to fearful faces. e: Anxiety (Case‐Control) PRS association with response to facial emotion as a proportion index.
**Figure S11**. Association of Anxiety (Factor Score) PRS across seven thresholds (Pt = 0.01, 0.05, 0.1, 0.2, 0.3, 0.4, 0.5) and the optimal threshold. b: Anxiety (Factor Score) PRS association with response to sad faces. c: Anxiety (Factor Score) PRS association with response to angry faces. d: Anxiety (Factor Score) PRS association with response to fearful faces. e: Anxiety (Factor Score) PRS association with response to facial emotion as a proportion index.
