# Supplementary material for: Genome‐wide association study of facial emotion recognition in children and association with polygenic risk for mental health disorders
Source: Am J Med Genet B Neuropsychiatr Genet. 2017 Jun 13;174(7):701–11. doi: 10.1002/ajmg.b.32558 (PMC5638097; doi:10.1002/ajmg.b.32558)
Supplement: Supplementary file 1 — Table S1. Distributional statistics for the phenotypes in the study, including measurements of internal consistency of the basic summed scores resulting from the DANVA. Table S2. Associations between unbiased hit rates of emotion recognition for each emotion and covariates. Positive betas indicate more accurate emotion recognition. Table S3. Locus information for top clumps associated with emotion recognition in GWAS. Base positions are hg19. Table S4. Linkage‐independent loci from sensitivity analyses, performing individual emotion GWAS without arcsine transformation of the phenotype. Loci with p < 5 × 10^−6^ in bold, sentinel SNPs from each analysis shaded gray. Each locus is represented by a sentinel SNP, that with the lowest p‐value in the locus. One locus on chromosome 7 showed different sentinel SNPs across different analyses, so is represented by three SNPs. Positive direction of effect means better recognition of emotion with each effect allele (A1). Table S5. Results from the sensitivity analysis without arcsine transformation for the sentinel SNPs listed in Table 3. SNPs with p < 5 × 10^−6^ are shown in bold. Sentinel SNPs from each of the four specific emotion analyses are shown in gray. b: Quantile‐quantile plot shows observed associations between genetic variants and recognition of happy faces (y‐axis) do not deviate from those expected under the null distribution (x‐axis). Lambda median is a measure of genomic inflation. Lambda ≈ 1, indicating minimal inflation due to confounds. Figure S1.Manhattan plot showing associations between genetic variants and recognition of happy faces. Base position of genetic variants on each chromosome are on the x‐axis, ‐log p‐value on the y‐axis. Genome‐wide significance (p = 5 × 10^−8^) is top line (red), and suggestive significance (p = 5 × 10^−6^) is bottom line (gray). Figure S2. Manhattan plot showing associations between genetic variants and recognition of sad faces. b: QQ plot showing no deviation from the null expectatio [file AJMG-174-701-s001.docx]

**Supplemental Material for "** **Genome-wide association study of facial emotion recognition in children, and association with polygenic risk for mental health disorders "**

**Supplementary Tables**

Supplementary Table 1

| 1. **Psychometric and distribution statistics for summed correct responses** | | | | | | | |
| --- | --- | --- | --- | --- | --- | --- | --- |
| **Measure** | **Cronbach's alpha** | **Mean** | **SD** | **Range** | **Skewness  (SE = 0.038)** | **Kurtosis (SE = 0.076)** | |
| Correct responses (All) | 0.64 | 19.4 | 2.70 | 3-24 | **-0.919** | **1.22** | |
| Correct responses (Happy) | 0.31 | 5.71 | 0.596 | 0-6 | -2.55 | 9.22 | |
| Correct responses (Sad) | 0.33 | 5.30 | 0.906 | 1-6 | **-1.39** | **1.87** | |
| Correct responses (Angry) | 0.49 | 3.94 | 1.34 | 0-6 | **-0.573** | **-0.245** | |
| Correct responses (Fearful) | 0.69 | 4.47 | 1.37 | 0-6 | **-1.26** | **1.58** | |
| 1. **Distribution statistics for proportion index and unbiased hit rates before transformation** | | | | | | | |
| **Measure** | | **Mean** | **SD** | **Range** | **Skewness  (SE = 0.038)** | | **Kurtosis (SE = 0.076)** |
| Proportion index | | 0.923 | 0.0560 | 0.3-1 | -1.98 | | 9.22 |
| Hit rate (Happy) | | 0.714 | 0.142 | 0-1 | **-0.361** | | **0.647** |
| Hit rate (Sad) | | 0.745 | 0.201 | 0.03-1 | **-0.604** | | **-0.136** |
| Hit rate (Angry) | | 0.608 | 0.244 | 0-1 | **-0.426** | | **-0.582** |
| Hit rate (Fearful) | | 0.664 | 0.250 | 0-1 | **-0.757** | | **0.042** |
| 1. **Distribution statistics for proportion index and unbiased hit rates after arcsine transformation** | | | | | | | |
| **Measure** | | **Mean** | **SD** | **Range** | **Skewness  (SE = 0.038)** | | **Kurtosis (SE = 0.076)** |
| Arcsine-transformed proportion index | | 1.31 | 0.102 | 0.58-1.57 | **-0.484** | | **1.70** |
| Arcsine-transformed hit rate (Happy) | | 1.03 | 0.191 | 0-1.57 | **0.749** | | 2.16 |
| Arcsine-transformed hit rate (Sad) | | 1.10 | 0.294 | 0.17-1.57 | **0.225** | | **-0.555** |
| Arcsine-transformed hit rate (Angry) | | 0.917 | 0.306 | 0-1.57 | **0.029** | | **0.281** |
| Arcsine-transformed hit rate (Fearful) | | 0.989 | 0.340 | 0-1.57 | **-0.327** | | **0.592** |

Supplementary Table 1: Distributional statistics for the phenotypes in the study, including measurements of internal consistency of the basic summed scores resulting from the DANVA.

Supplementary Table 2

| **Covariate** | **Happy** | | **Sad** | | **Fearful** | | **Angry** | |
| --- | --- | --- | --- | --- | --- | --- | --- | --- |
|  | **B** | ***P*** | **B** | ***p*** | **B** | ***p*** | **B** | ***p*** |
| Female gender | 0.0202 | **7.26x10^-4^** | 0.0593 | **8.19x10^-11^** | 0.00440 | 0.675 | 0.0495 | **1.73x10^-7^** |
| Age | 0.00104 | **3.44x10^-5^** | 0.00203 | **1.34x10^-7^** | 0.00151 | **6.48x10^-4^** | 0.00271 | **1.32x10^-11^** |
| IQ | 0.00113 | **4.08x10^-9^** | 0.00227 | **1.06x10^-14^** | 0.00379 | **7.55x10^-29^** | 0.00228 | **6.90x10^-14^** |
| Activities Session  Second | 0.0177 | 0.064 | 0.0452 | **0.00190** | 0.0502 | **0.00283** | 0.0757 | **5.57x10^-7^** |
| Activities Session  Third | 0.00989 | 0.3178 | 0.0326 | **0.0306** | 0.00962 | 0.581 | 0.0373 | 0.0171 |
| Activities Session  Fourth | 0.00303 | 0.6605 | 0.0131 | 0.213 | -0.00404 | 0.740 | 0.0244 | 0.0256 |
| SCDC | -0.00355 | **6.44x10^-5^** | -0.00337 | **0.0126** | -0.00676 | **1.50x10^-5^** | -0.00512 | **2.62x10^-4^** |

Supplementary Table 2: Associations between unbiased hit rates of emotion recognition for each emotion and covariates. Positive betas indicate more accurate emotion recognition.

Supplementary Table 3

| **Independent clumps associated with emotion recognition with *p*<5x10^-6^** | | | | | | |
| --- | --- | --- | --- | --- | --- | --- |
| **Sentinel SNP** | **A1** | **CHR** | **A1 Freq** | **Imputation R^2^** | **Clump BP** | **Genes +/- 100kb** |
|  |  |  |  |  |  |  |
| rs9550616 | A | 13 | 0.355 | 0.817 | 20718089 - 20748357 | *ZMYM2, GJA3, GJB2, GJB6* |
| rs3770081 | G | 2 | 0.9622 | 0.462 | 86280925 - 86395807 | *POLR1A, PTCD3, SNORD94, IMMT, MRPL35, REEP1* |
| rs12705054 | A | 7 | 0.9364 | 0.982 | 98730819 - 99052428 | *SMURF1, KPNA7, MYH16, ARPC1A, ARPC1B, ARC41, PDAP1, BUD31, PTCD1, CPSF4, ATP5J2, ZNF789, ZNF394, ZKSCAN5, KIAA1015, C7orf38* |
| rs2080301  rs17604090  rs10248839 | A A C | 7 | 0.673  0.151 0.160 | 0.997 0.999  0.881 | 29635526 - 29754941 | *CHN2, PRR15, WIPF3* |
| rs1146849 | A | 13 | 0.278 | 0.981 | 72822770 - 72890603 | *-* |
| rs654861 | A | 6 | 0.968 | 0.811 | 117487047 - 117565744 | *VGLL2, ROS1, GOPC* |
| rs2304503 | A | 3 | 0.522 | 0.979 | 78676116 - 78979741 | *ROBO1* |
| rs10499395 | G | 7 | 0.068 | 0.994 | 11911677 - 11960047 | *THSD7A* |
| rs4930838 | A | 12 | 0.156 | 0.912 | 29029125 - 29101922 | *-* |
| rs683257 | A | 6 | 0.909 | 0.986 | 140877781 - 141222059 | *-* |
| rs17016200 | G | 3 | 0.855 | 1 | 78415391 - 78584757 | *ROBO1* |
| rs1423494 | C | 5 | 0.415 | 0.982 | 11333749 - 11381138 | *CTNND2* |

Supplementary Table 3: Locus information for top clumps associated with emotion recognition in GWAS. Base positions are hg19.

Supplementary Table 4

| **Independent clumps associated with emotion recognition with *p*<5x10^-6^ without arcsine transformation** | | | | | | | | | | |
| --- | --- | --- | --- | --- | --- | --- | --- | --- | --- | --- |
| **Sentinel SNP** | **A1** | **CHR** | **Happy** | | **Sad** | | **Fearful** | | **Angry** | |
|  |  |  | **Z** | ***p*** | **Z** | ***p*** | **Z** | ***p*** | **Z** | ***p*** |
| rs8016700 | A | 14 | **-4.74** | **2.20x10^-6^** | -2.51 | 0.0120 | -2.42 | 0.0157 | -1.89 | 0.0591 |
| rs11582690 | A | 1 | **-4.68** | **3.01x10^-6^** | -0.615 | 0.539 | -1.59 | 0.113 | -0.0806 | 0.936 |
| rs11881372 | A | 19 | **4.65** | **3.50x10^-6^** | 2.34 | 0.0191 | 3.82 | 1.38x10^-4^ | 2.35 | 0.0188 |
| rs6812996 | A | 4 | -2.03 | 0.0426 | **-4.91** | **9.38x10^-7^** | -2.79 | 0.00536 | -3.18 | 0.00149 |
| rs10257155 | G | 7 | 3.25 | 0.00117 | **4.80** | **1.66x10^-6^** | 2.19 | 0.0283 | 3.56 | 3.80x10^-4^ |
| rs17604090 | A | 7 | 2.95 | 0.00316 | **4.61** | **4.07x10^-6^** | 3.06 | 0.00223 | 4.30 | 1.73x10^-5^ |
| rs654861 | A | 6 | 2.46 | 0.0138 | 1.46 | 0.145 | **5.10** | **3.57x10^-7^** | 2.20 | 0.0281 |
| rs9907824 | G | 17 | 2.81 | 0.00501 | 3.15 | 0.00164 | **4.83** | **1.40x10^-6^** | 3.35 | 8.02x10^-4^ |
| rs16949992 | C | 15 | 2.68 | 0.00734 | 1.04 | 0.298 | **4.69** | **2.88x10^-6^** | 2.15 | 0.0317 |
| rs664533 | C | 1 | -3.01 | 0.00267 | -2.20 | 0.0277 | **-4.65** | **3.50x10^-6^** | -1.87 | 0.0621 |
| rs12415661 | C | 10 | 1.22 | 0.222 | 2.95 | 0.00316 | 1.31 | 0.189 | **4.92** | **9.14x10^-7^** |
| rs10499395 | G | 7 | 2.88 | 0.00398 | 2.33 | 0.0199 | 1.71 | 0.0881 | **4.65** | **3.38x10^-6^** |
| rs4366679 | C | 15 | 2.48 | 0.0132 | -1.13 | 0.257 | -1.42 | 0.156 | **-4.57** | **4.91x10^-6^** |

Supplementary Table 4: Linkage-independent loci from sensitivity analyses, performing individual emotion GWAS without arcsine transformation of the phenotype. Loci with *p*<5x10^-6^ in bold, sentinel SNPs from each analysis shaded grey. Each locus is represented by a sentinel SNP, that with the lowest *p*-value in the locus. One locus on chromosome 7 showed different sentinel SNPs across different analyses, so is represented by three SNPs. Positive direction of effect means better recognition of emotion with each effect allele (A1).

Supplementary Table 5

| **Statistics of sentinel SNPs from main analysis in analysis without arcsine transformation** | | | | | | | | | | |
| --- | --- | --- | --- | --- | --- | --- | --- | --- | --- | --- |
| **Sentinel SNP** | **A1** | **CHR** | **Happy** | | **Sad** | | **Fearful** | | **Angry** | |
|  |  |  | **Z** | ***p*** | **Z** | ***P*** | **Z** | ***p*** | **Z** | ***p*** |
| rs9550616 | A | 13 | -4.44 | 9.06x10^-6^ | -2.10 | 0.0362 | -1.42 | 0.156 | -2.61 | 0.00922 |
| rs3770081 | G | 2 | -1.75 | 0.0807 | -4.53 | 6.15x10^-6^ | -0.991 | 0.322 | -4.14 | 3.47x10^-5^ |
| rs12705054 | A | 7 | -1.76 | 0.0784 | -4.46 | 8.23x10^-6^ | -2.22 | 0.0265 | -3.51 | 4.47x10^-4^ |
| rs2080301  rs17604090  rs10248839 | A A C | 7 | -3.68  2.95  3.29 | 2.33x10^-4^ 0.00316 0.00101 | -3.73  **4.61**  4.10 | 1.96x10^-4^ **4.07x10^-6^** 4.14x10^-5^ | -2.91  3.06  2.96 | 0.00364 0.00223 0.00312 | -3.91 4.30 4.40 | 9.36x10^-5^ 1.73x10^-5^ 1.06x10^-5^ |
| rs1146849 | A | 13 | -1.26 | 0.204 | -4.22 | 2.47x10^-5^ | -1.39 | 0.164 | -4.13 | 3.71x10^-5^ |
| rs654861 | A | 6 | 2.46 | 0.0138 | 1.46 | 0.145 | **5.10** | **3.57x10^-7^** | 2.20 | 0.0281 |
| rs2304503 | A | 3 | -3.11 | 0.00188 | -0.425 | 0.671 | -4.38 | 1.16x10^-5^ | -0.541 | 0.588 |
| rs10499395 | G | 7 | 2.88 | 0.00398 | 2.33 | 0.0199 | 1.71 | 0.0881 | **4.65** | **3.38x10^-6^** |
| rs4930838 | A | 12 | 0.318 | 0.750 | 2.31 | 0.0212 | 0.614 | 0.539 | 4.47 | 7.85x10^-6^ |
| rs683257 | A | 6 | -2.16 | 0.0309 | -2.64 | 0.00826 | -2.25 | 0.0248 | -4.27 | 1.96x10^-5^ |
| rs17016200 | G | 3 | 2.63 | 0.00863 | 4.29 | 1.81x10^-5^ | 3.68 | 2.36x10^-4^ | 4.09 | 4.42x10^-5^ |
| rs1423494 | C | 5 | -3.78 | 1.57x10^-4^ | -3.74 | 1.83x10^-4^ | -3.56 | 3.80x10^-4^ | -3.57 | 3.67x10^-4^ |

Supplementary Table 5: Results from the sensitivity analysis without arcsine transformation for the sentinel SNPs listed in Table 3. SNPs with p < 5x10^-6^ are shown in bold. Sentinel SNPs from each of the four specific emotion analyses are shown in grey.

**Supplementary Figures**

Supplementary Figure 1


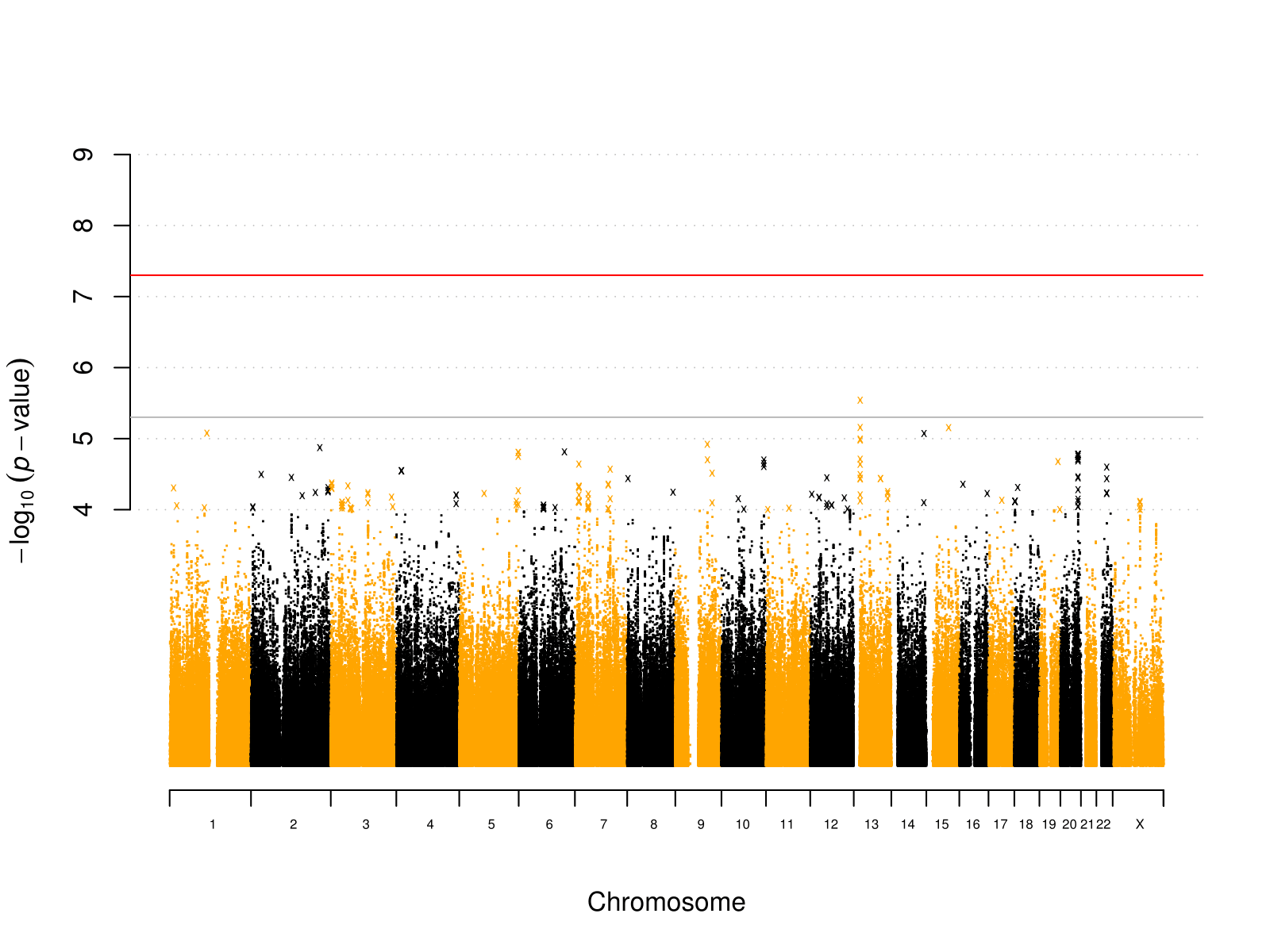


Supplementary Figure 1a: Manhattan plot showing associations between genetic variants and recognition of happy faces. Base position of genetic variants on each chromosome are on the x-axis, -log *p-*value on the y-axis. Genome-wide significance (*p*=5x10^-8^) is top line (red), and suggestive significance (*p*=5x10^-6^) is bottom line (grey).


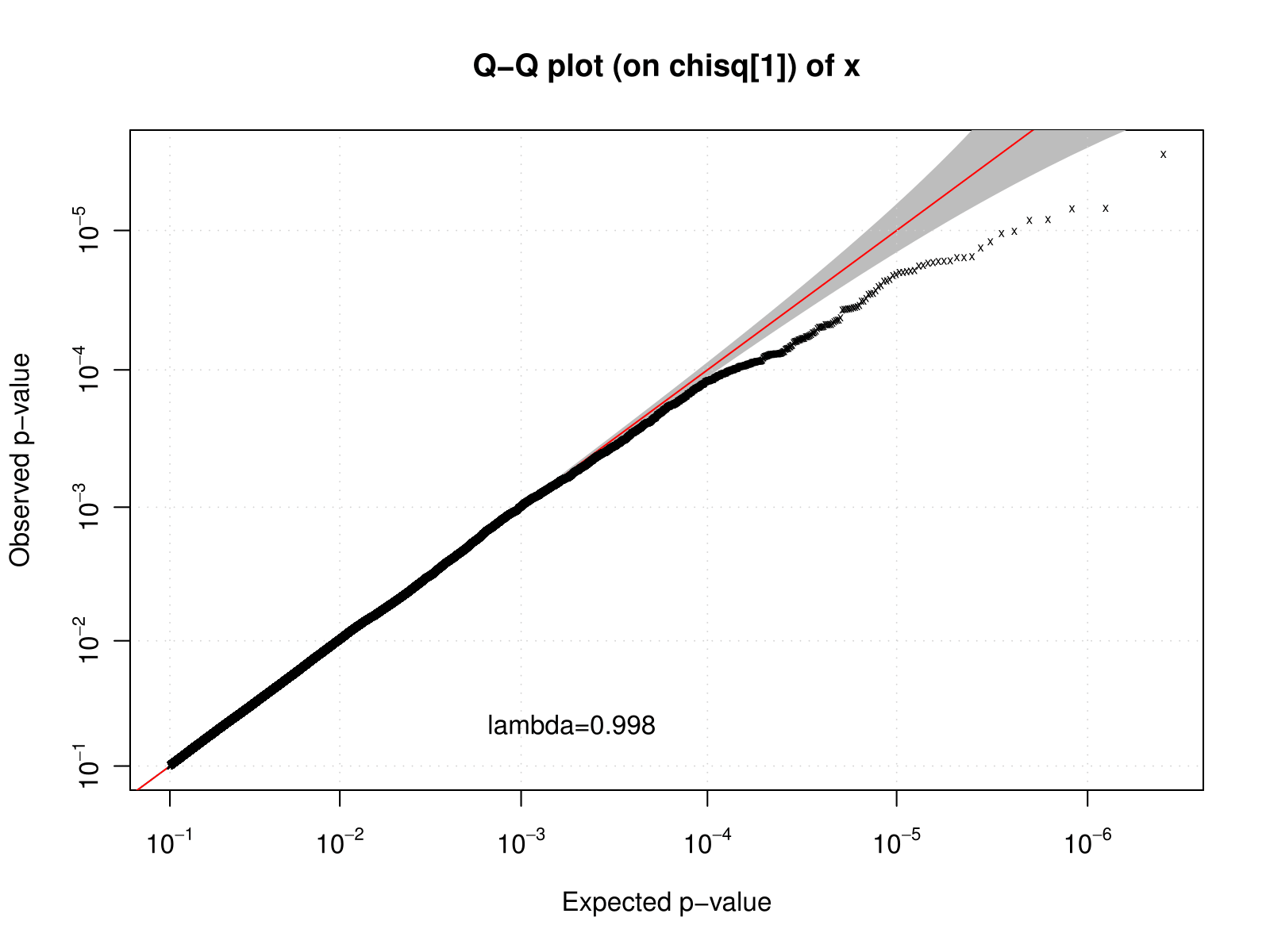


Supplementary Figure 1b: Quantile-quantile plot shows observed associations between genetic variants and recognition of happy faces (y-axis) do not deviate from those expected under the null distribution (x-axis). Lambda median is a measure of genomic inflation. Lambda ≈ 1, indicating minimal inflation due to confounds.

Supplementary Figure 2


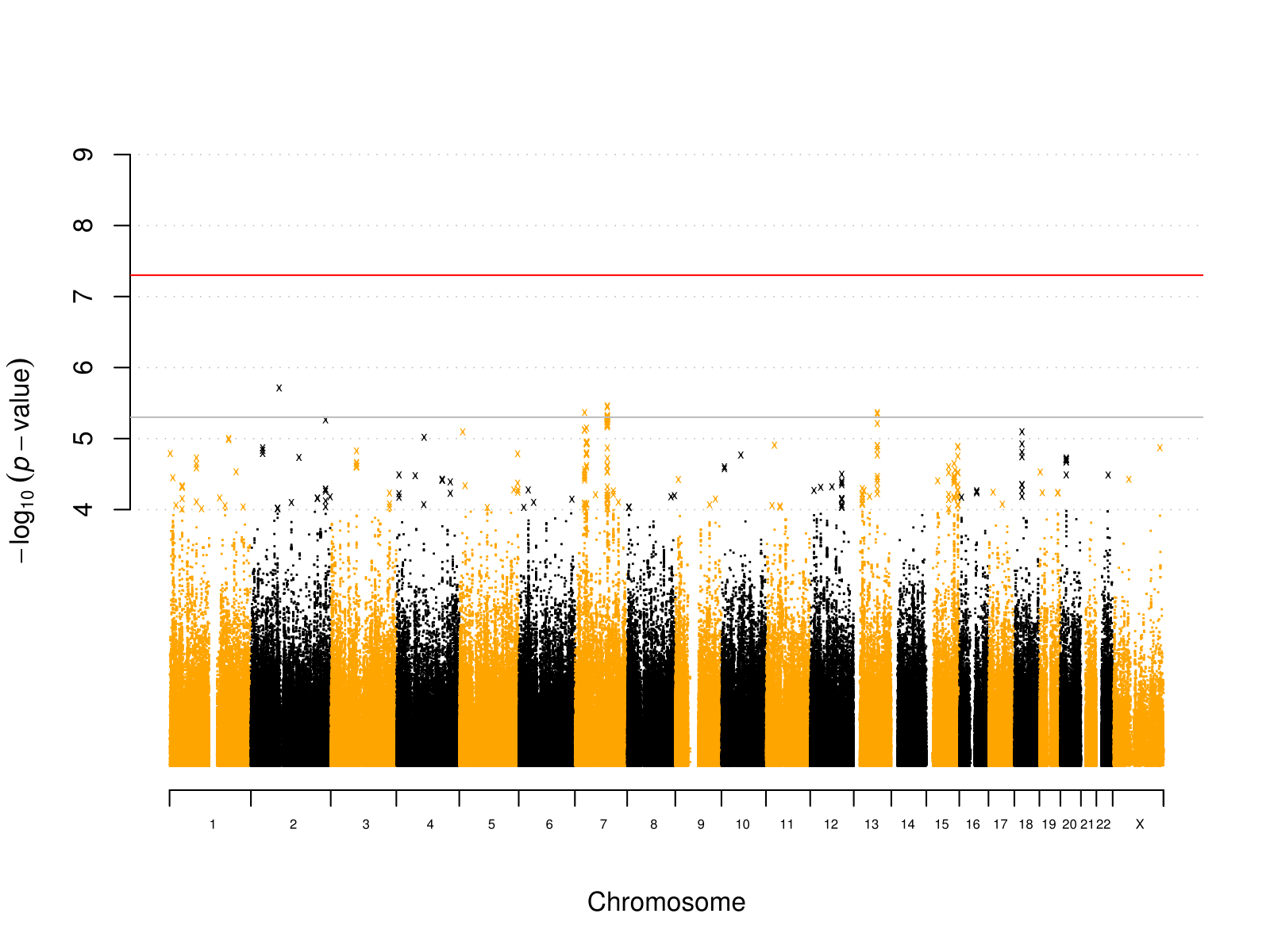


Supplementary Figure 2a: Manhattan plot showing associations between genetic variants and recognition of sad faces.


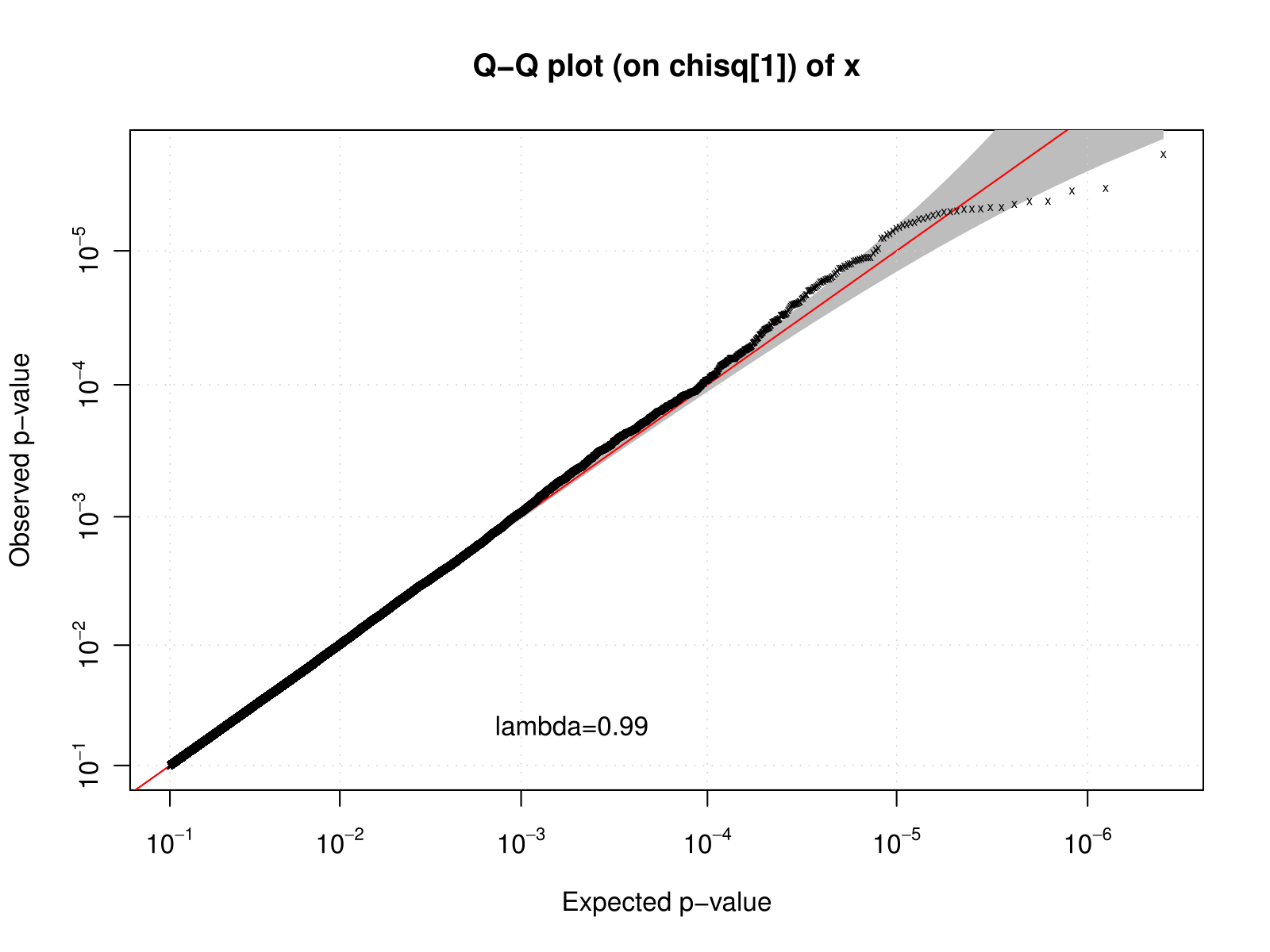


Supplementary Figure 2b: QQ plot showing no deviation from the null expectation for genetic variants and recognition of sad faces.

Supplementary Figure 3


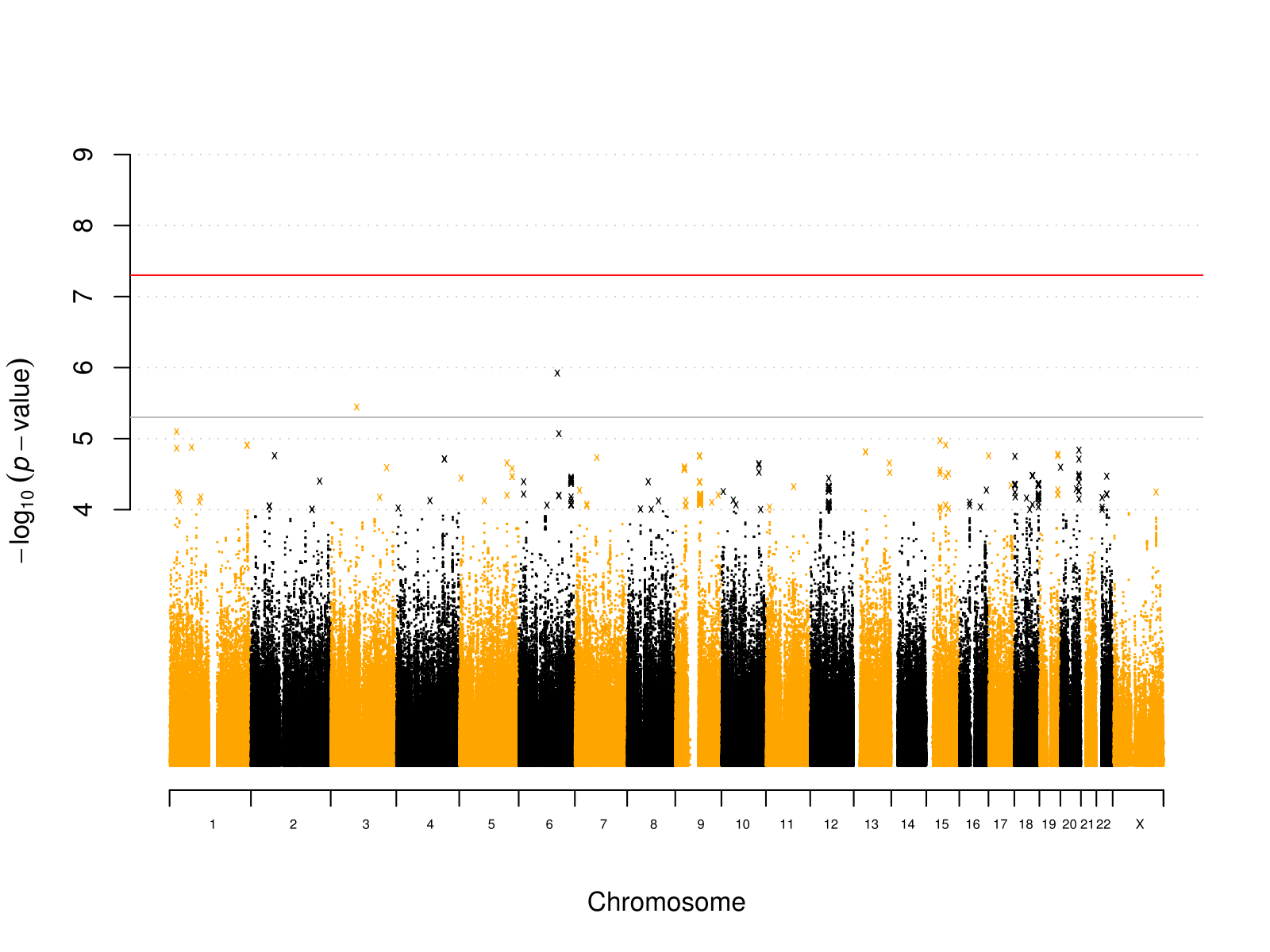


Supplementary Figure 3a: Manhattan plot showing associations between genetic variants and recognition of fearful faces.


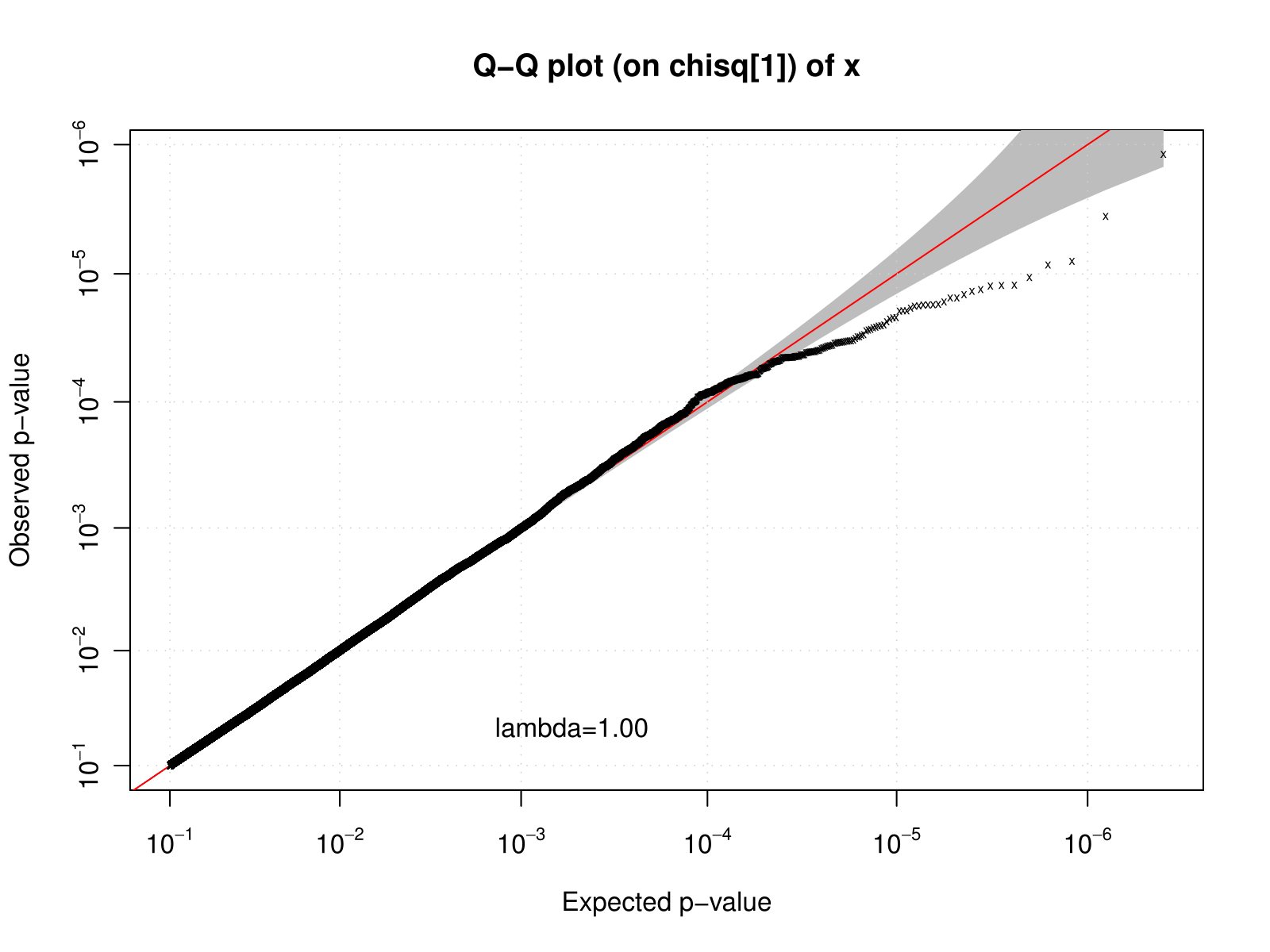


Supplementary Figure 3b: QQ plot showing no deviation from the null expectation for genetic variants and recognition of fearful faces.

Supplementary Figure 4


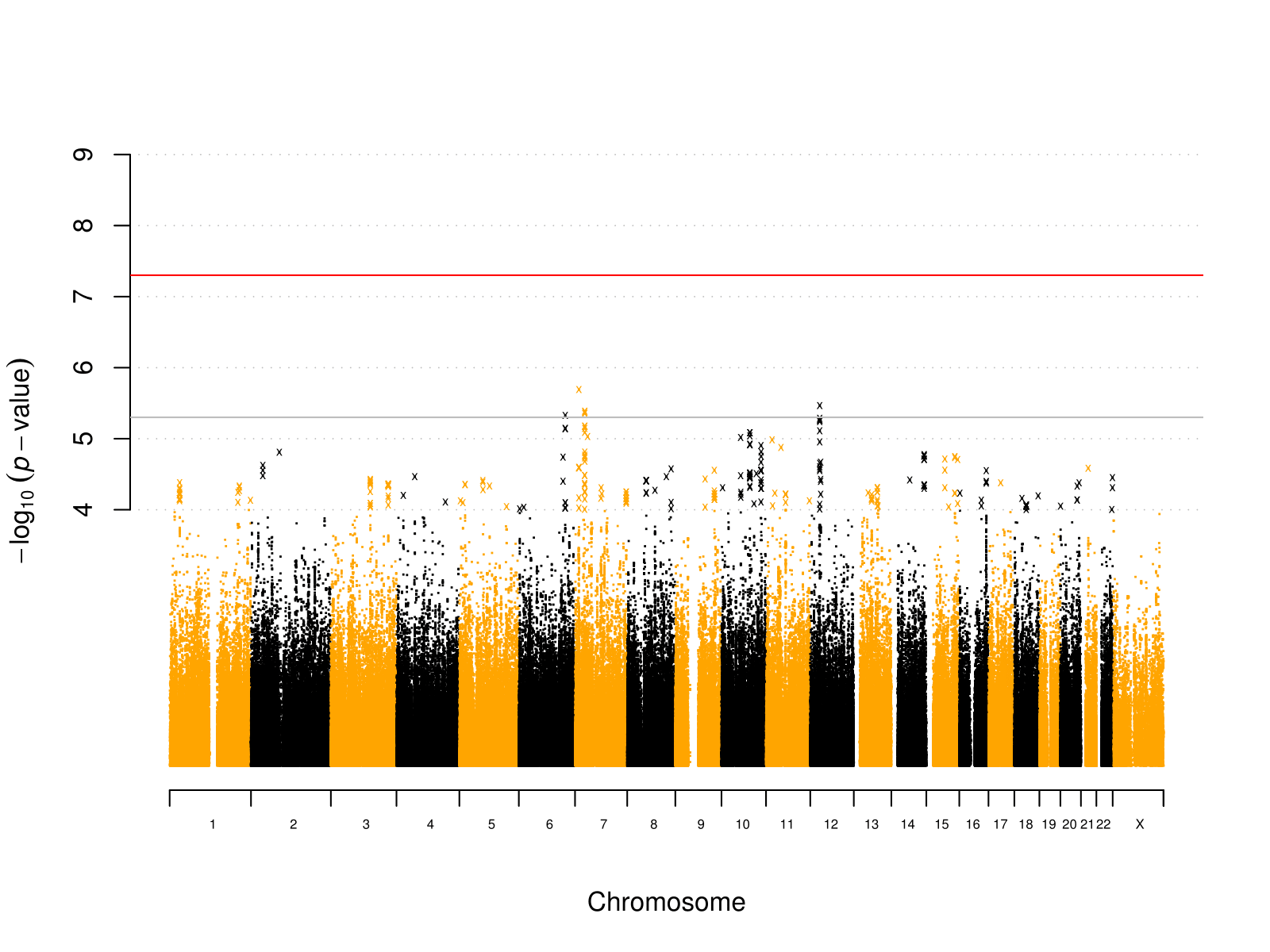


Supplementary Figure 4a: Manhattan plot showing associations between genetic variants and recognition of angry faces.


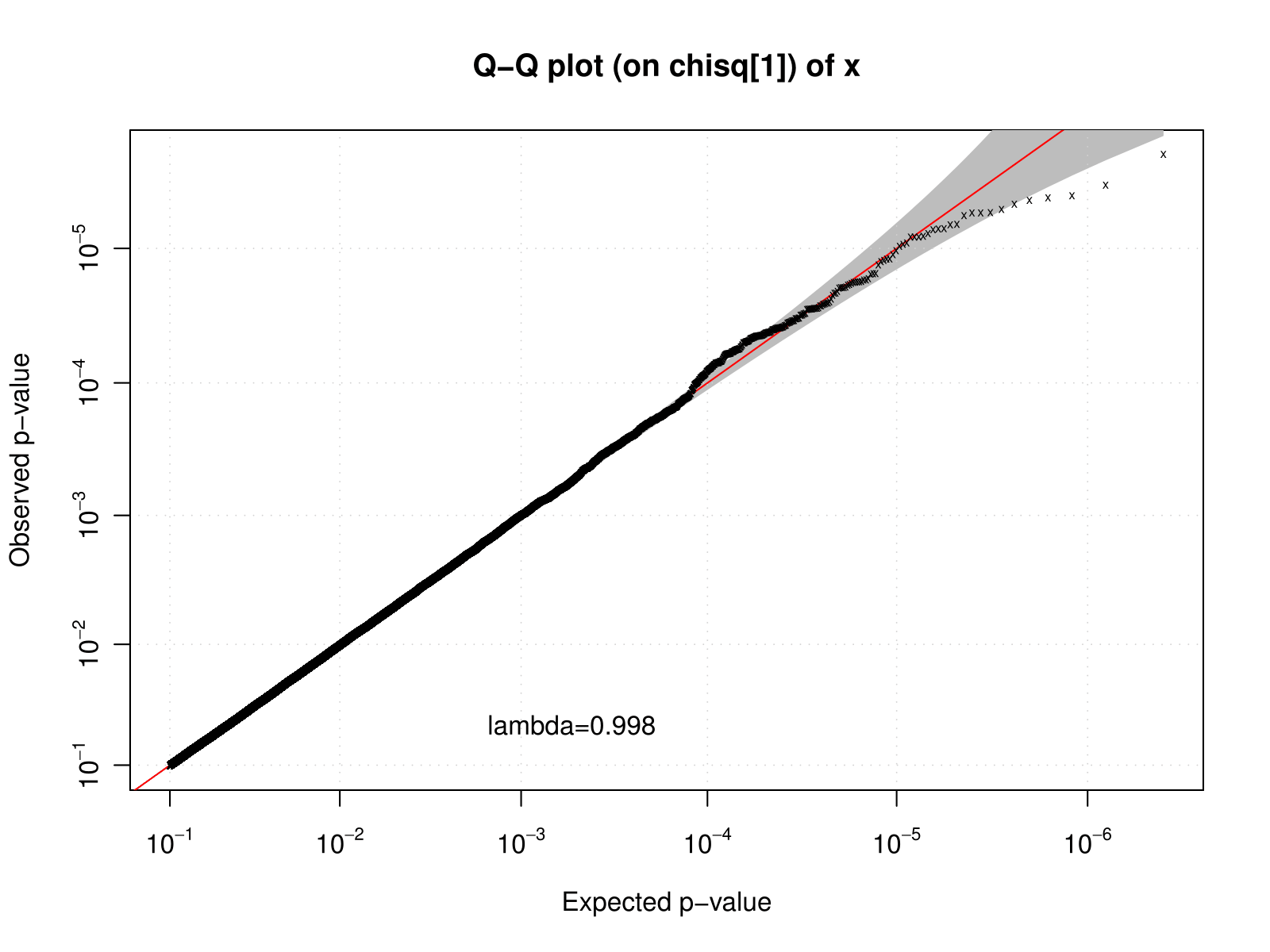


Supplementary Figure 4b: QQ plot showing no deviation from the null expectation for genetic variants and recognition of angry faces.

Supplementary Figure 5

Supplementary Figure 5: Association of Schizophrenia PRS across seven thresholds
(Pt = 0.01, 0.05, 0.1, 0.2, 0.3, 0.4, 0.5) and the optimal threshold.


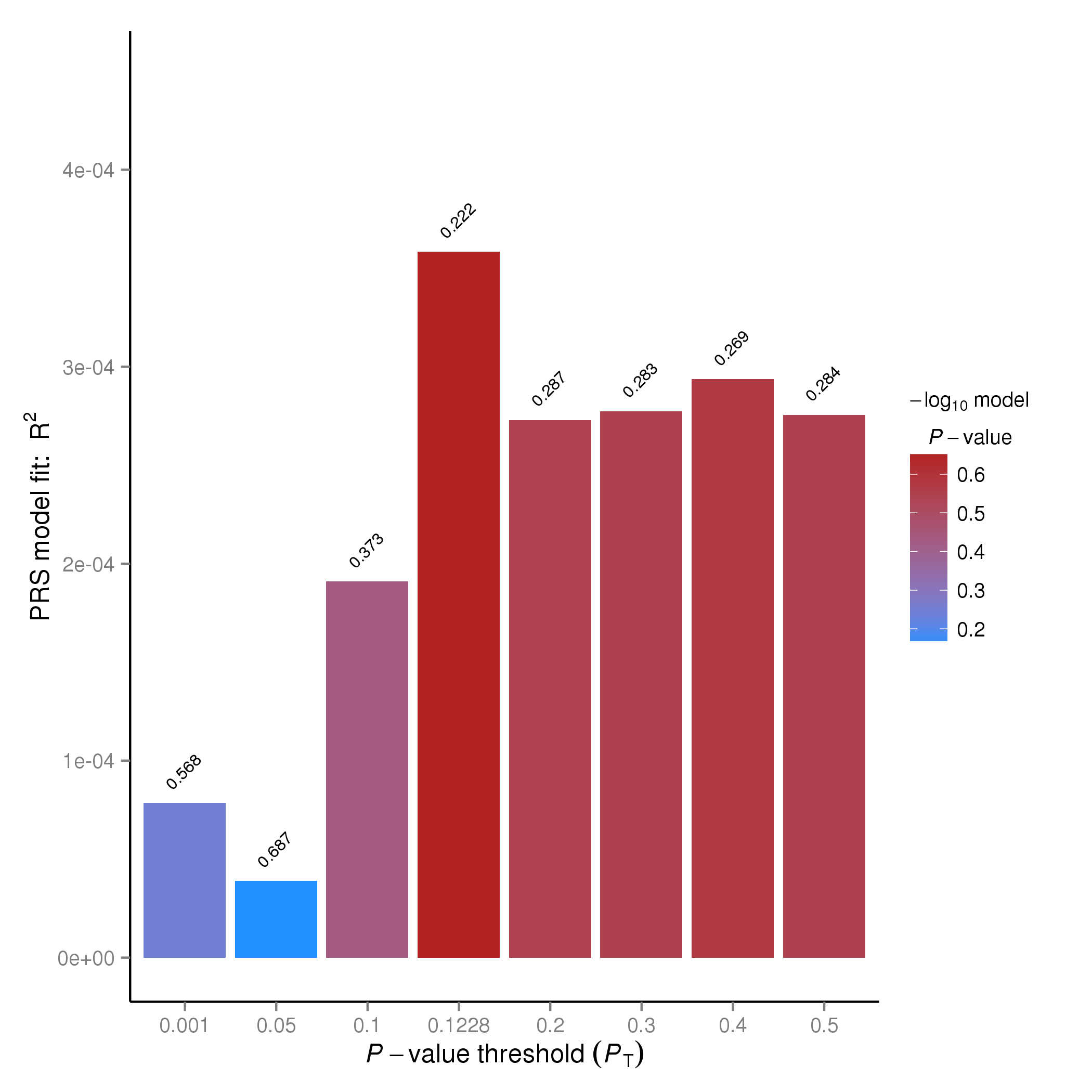


Supplementary Figure 5a: Schizophrenia PRS association with response to happy faces


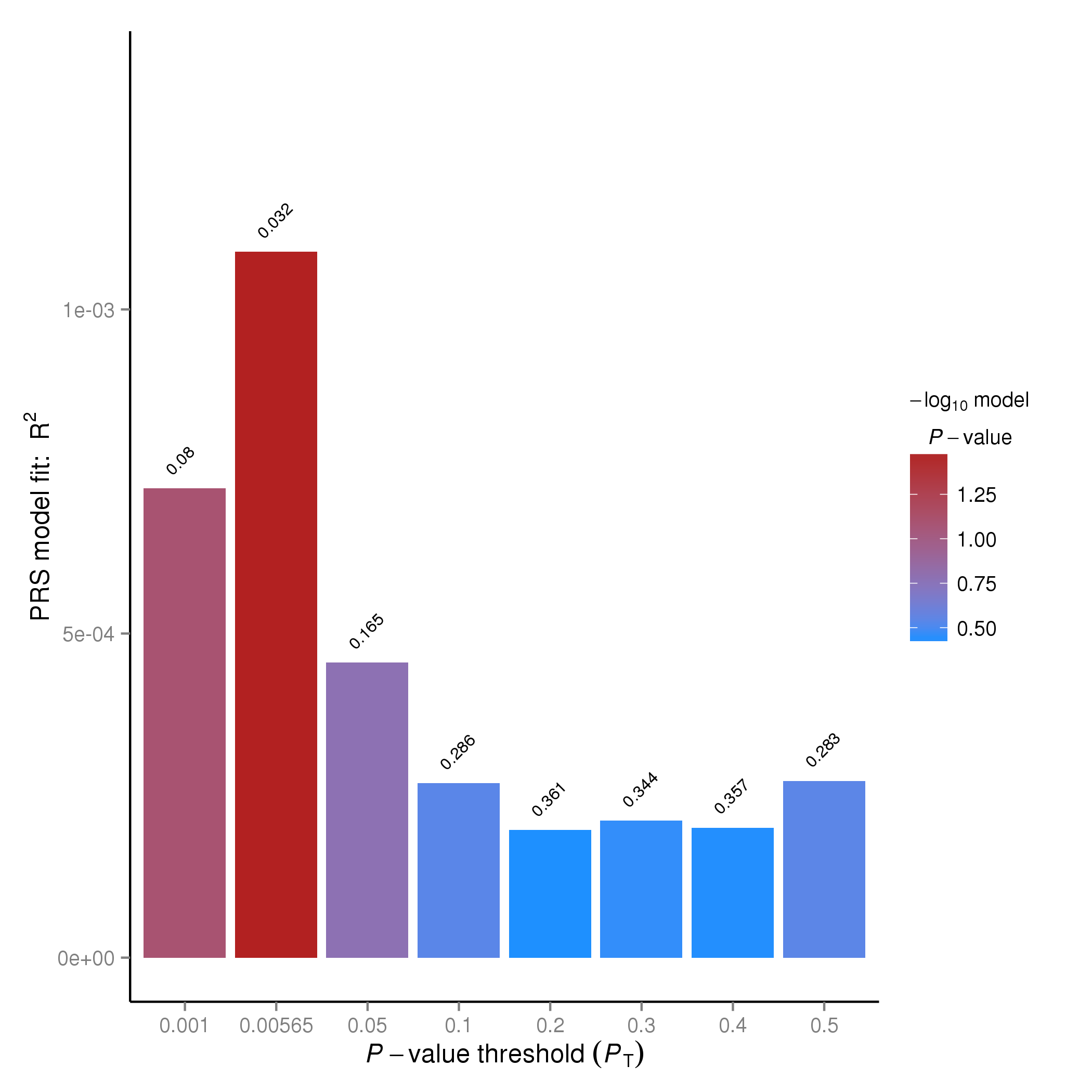


Supplementary Figure 5b: Schizophrenia PRS association with response to sad faces


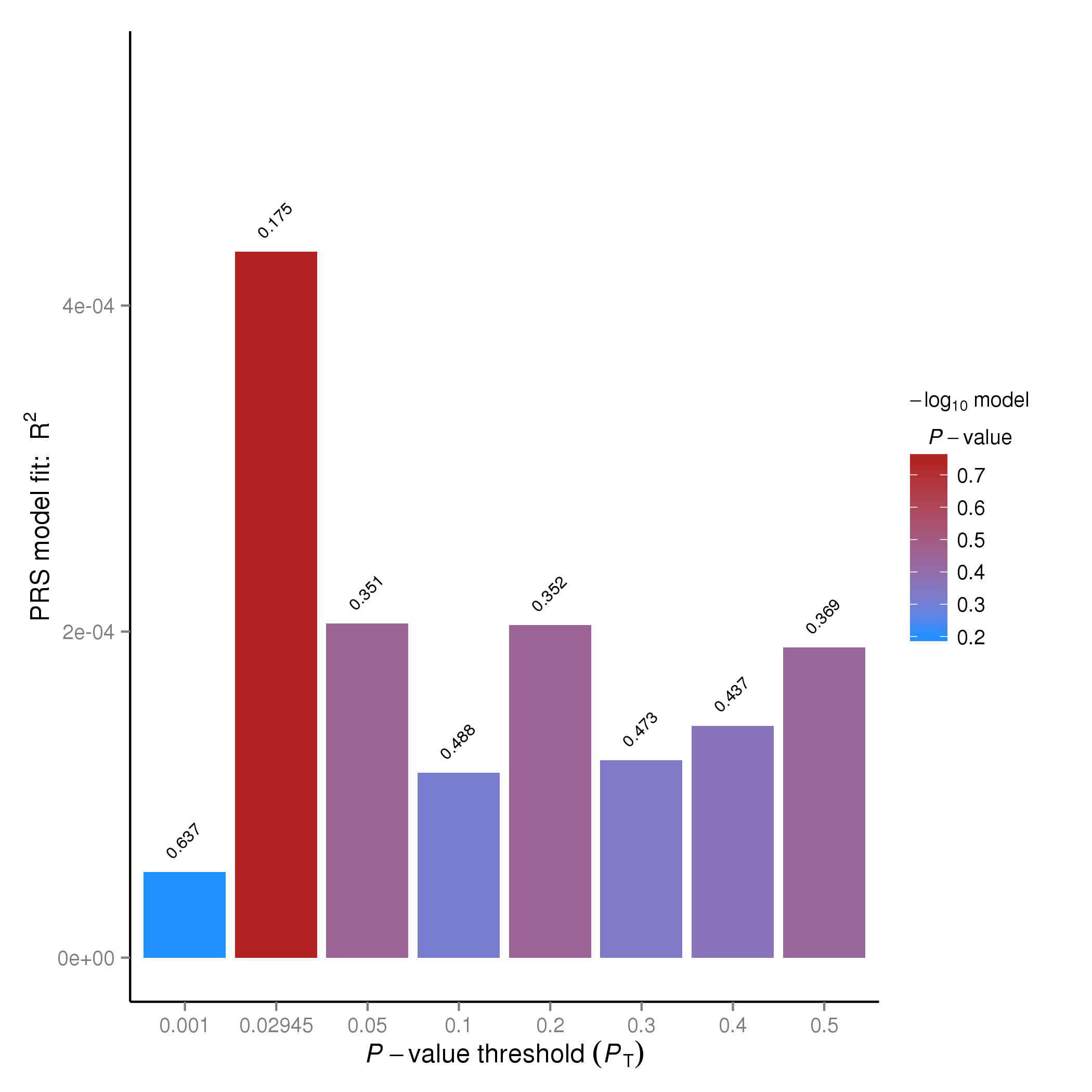


Supplementary Figure 5c: Schizophrenia PRS association with response to angry faces


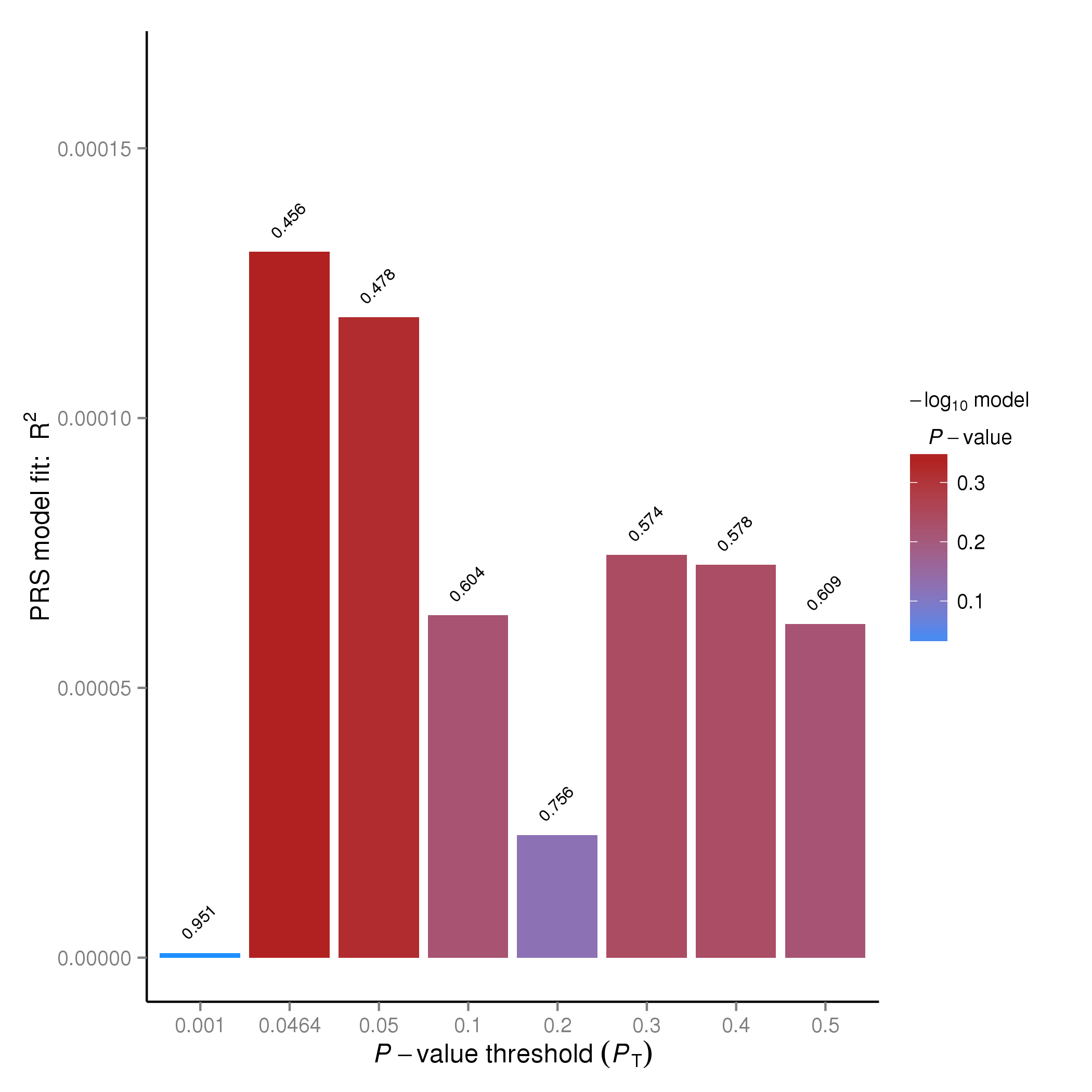


Supplementary Figure 5d: Schizophrenia PRS association with response to fearful faces


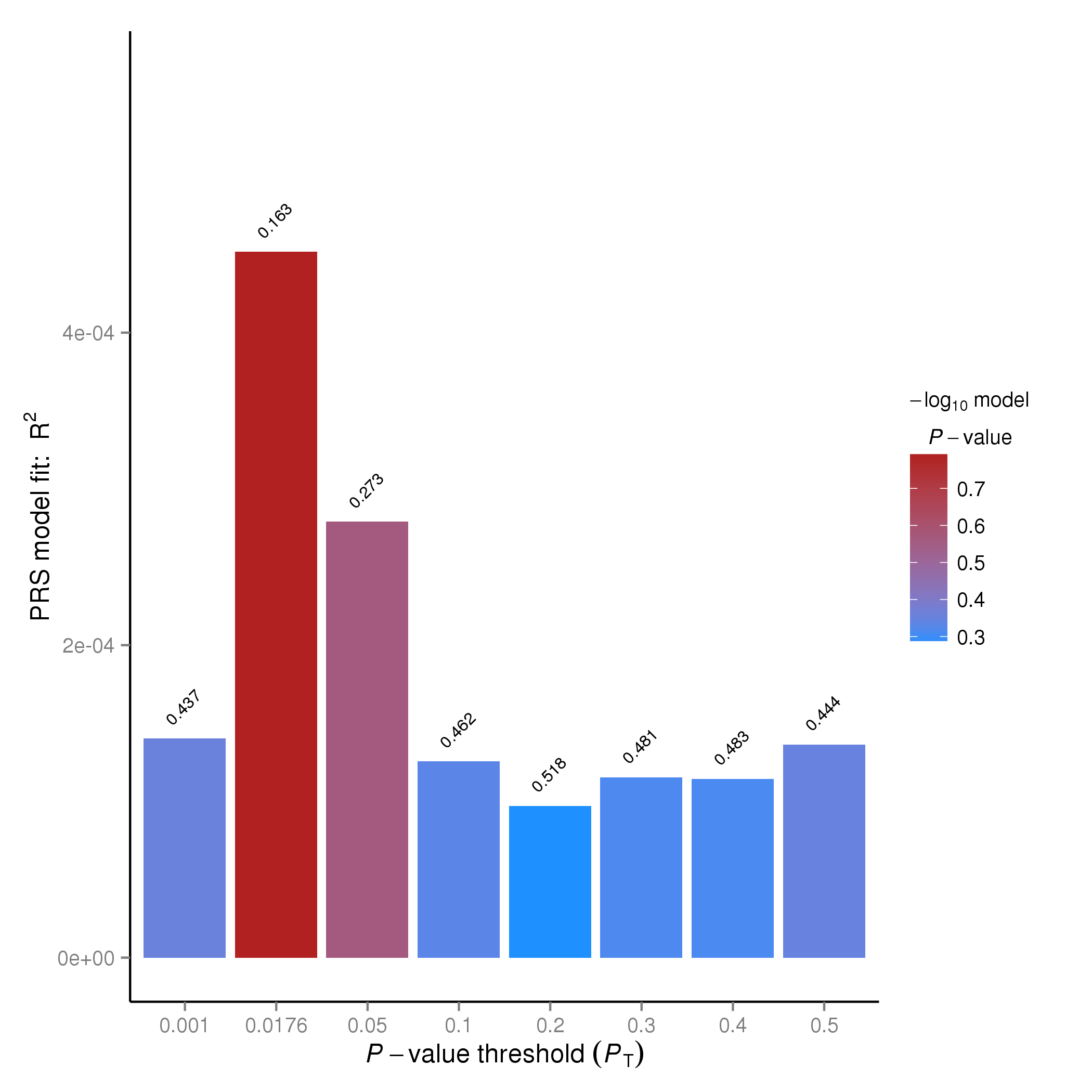


Supplementary Figure 5e: Schizophrenia PRS association with response to facial emotion as a proportion index

Supplementary Figure 6

Supplementary Figure 6: Association of Bipolar Disorder PRS across seven thresholds
(Pt = 0.01, 0.05, 0.1, 0.2, 0.3, 0.4, 0.5) and the optimal threshold.


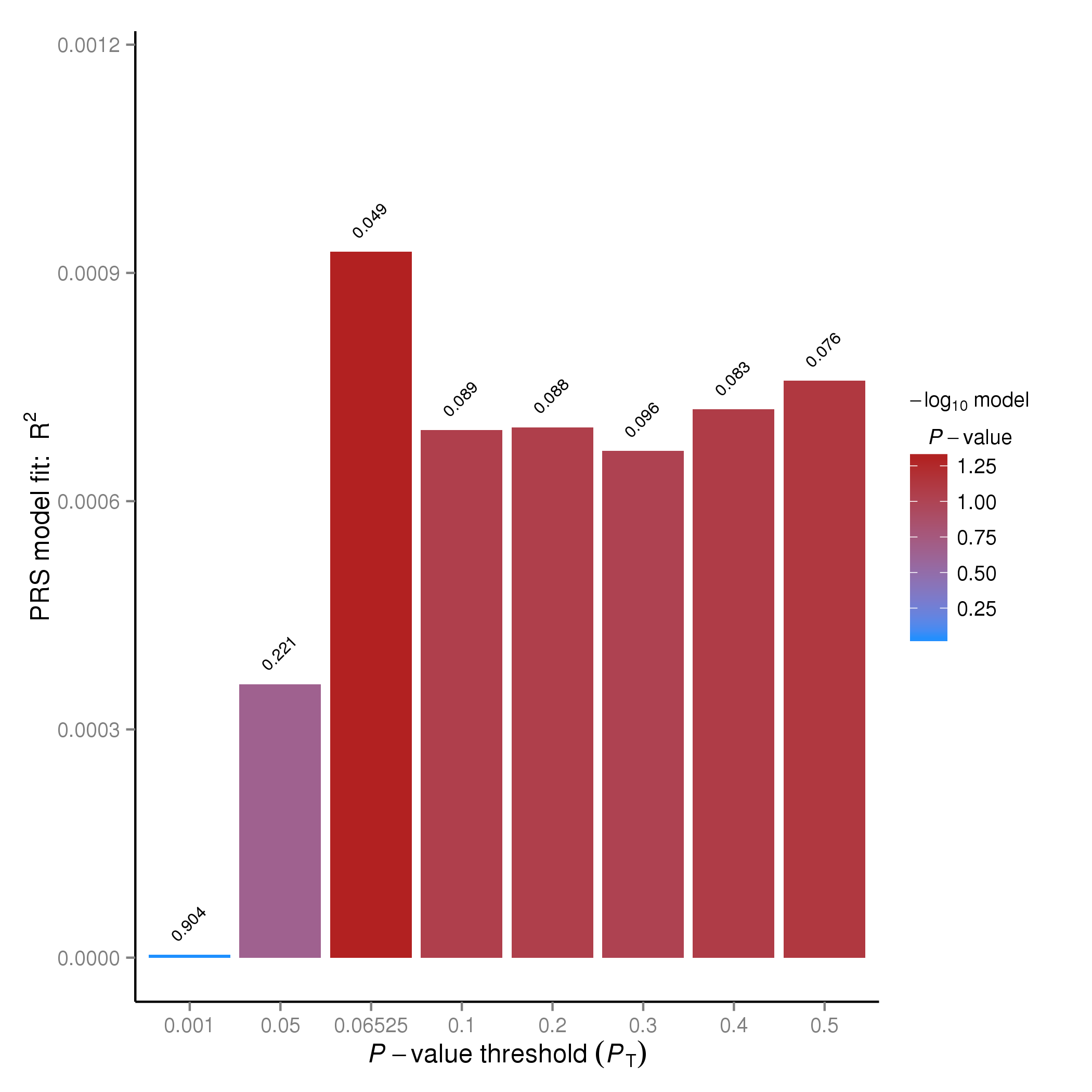


Supplementary Figure 6a: Bipolar Disorder PRS association with response to happy faces


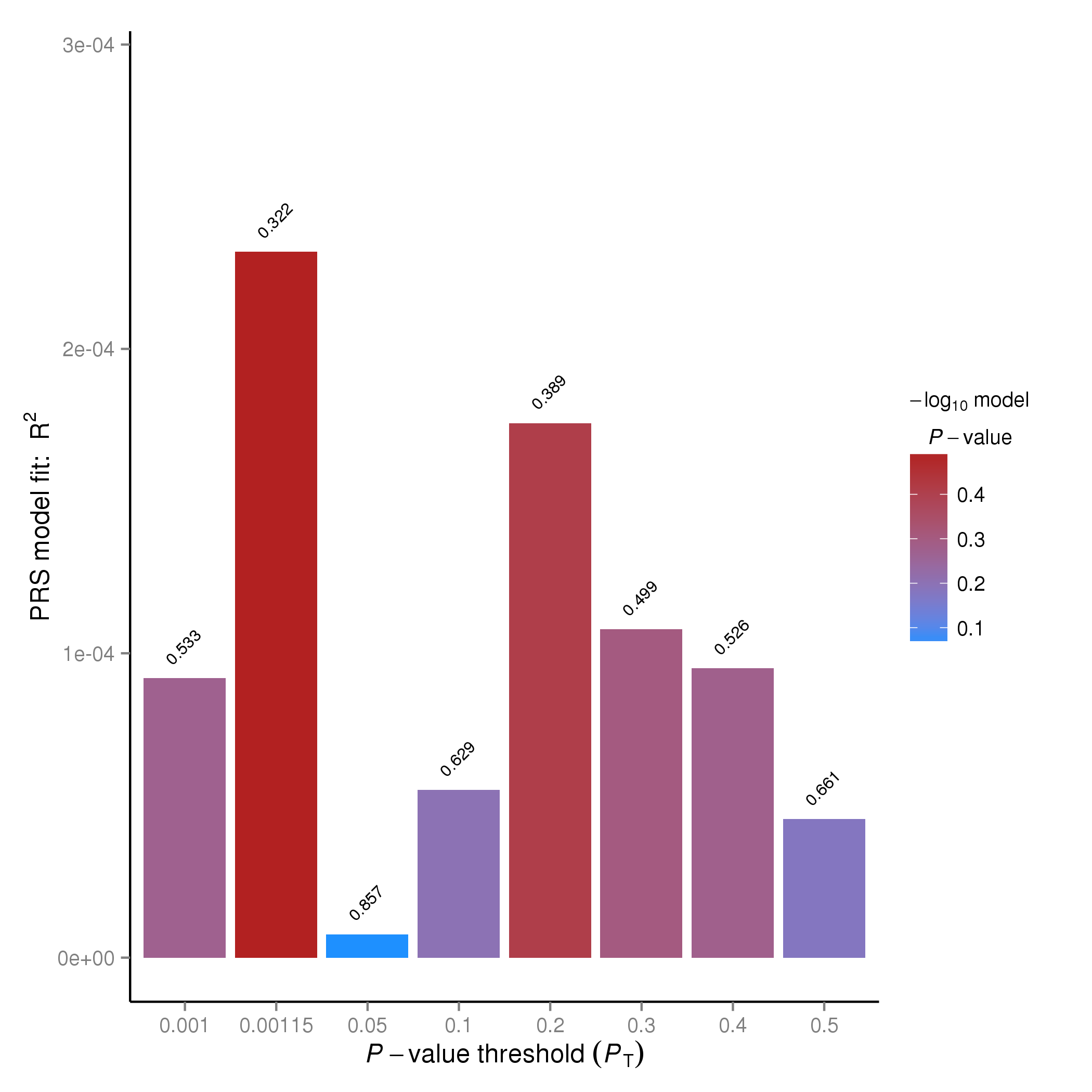


Supplementary Figure 6b: Bipolar Disorder PRS association with response to sad faces


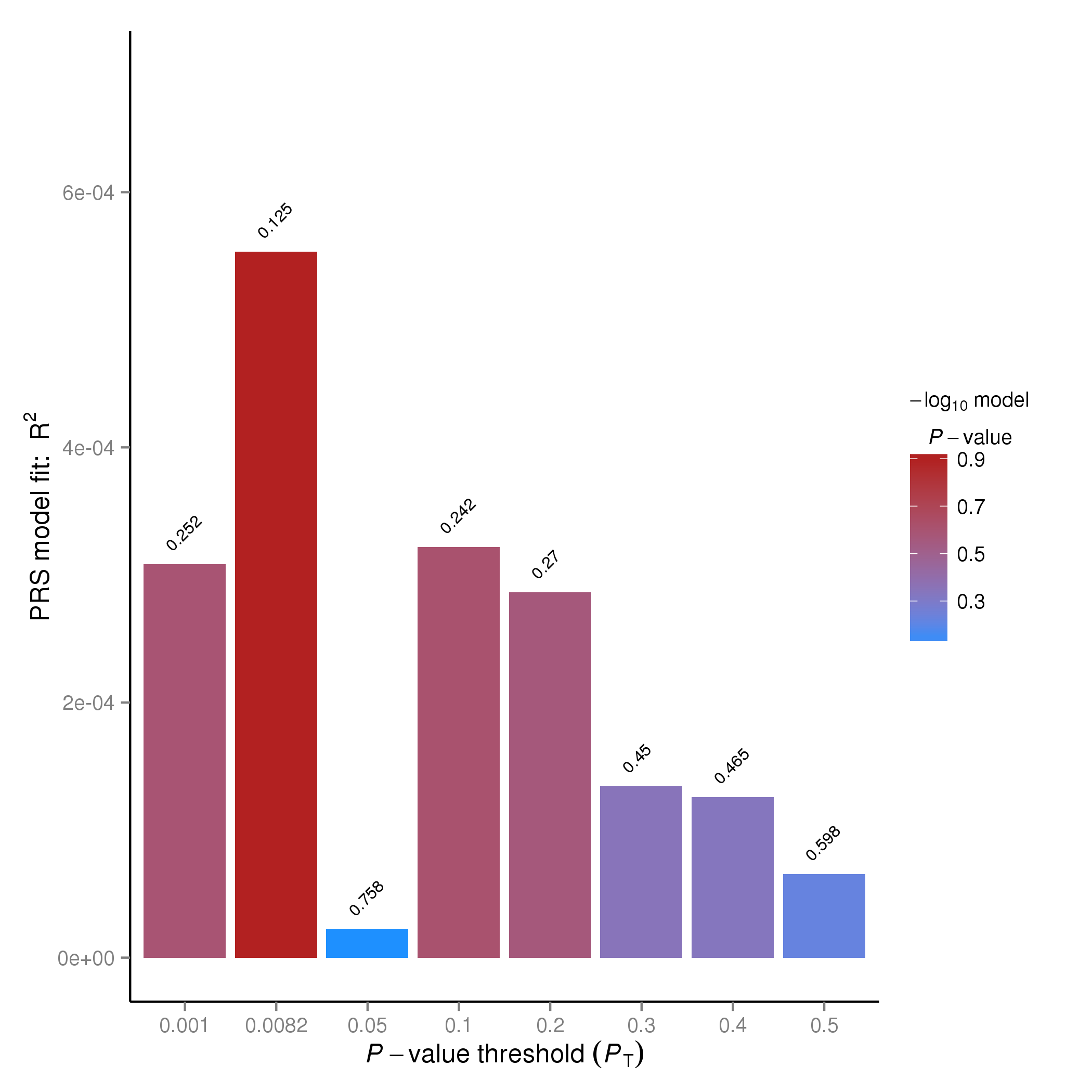


Supplementary Figure 6c: Bipolar Disorder PRS association with response to angry faces


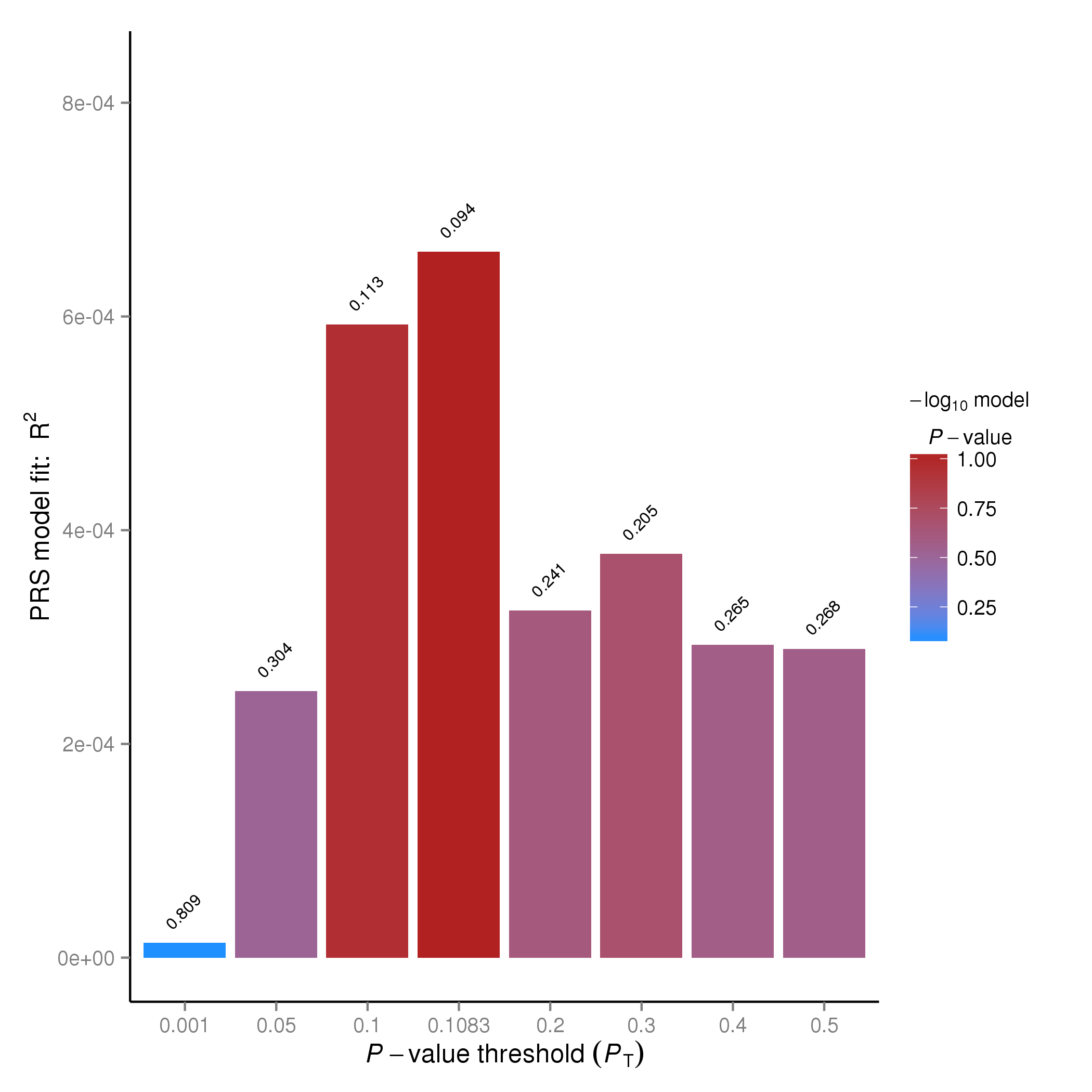


Supplementary Figure 6d: Bipolar Disorder PRS association with response to fearful faces


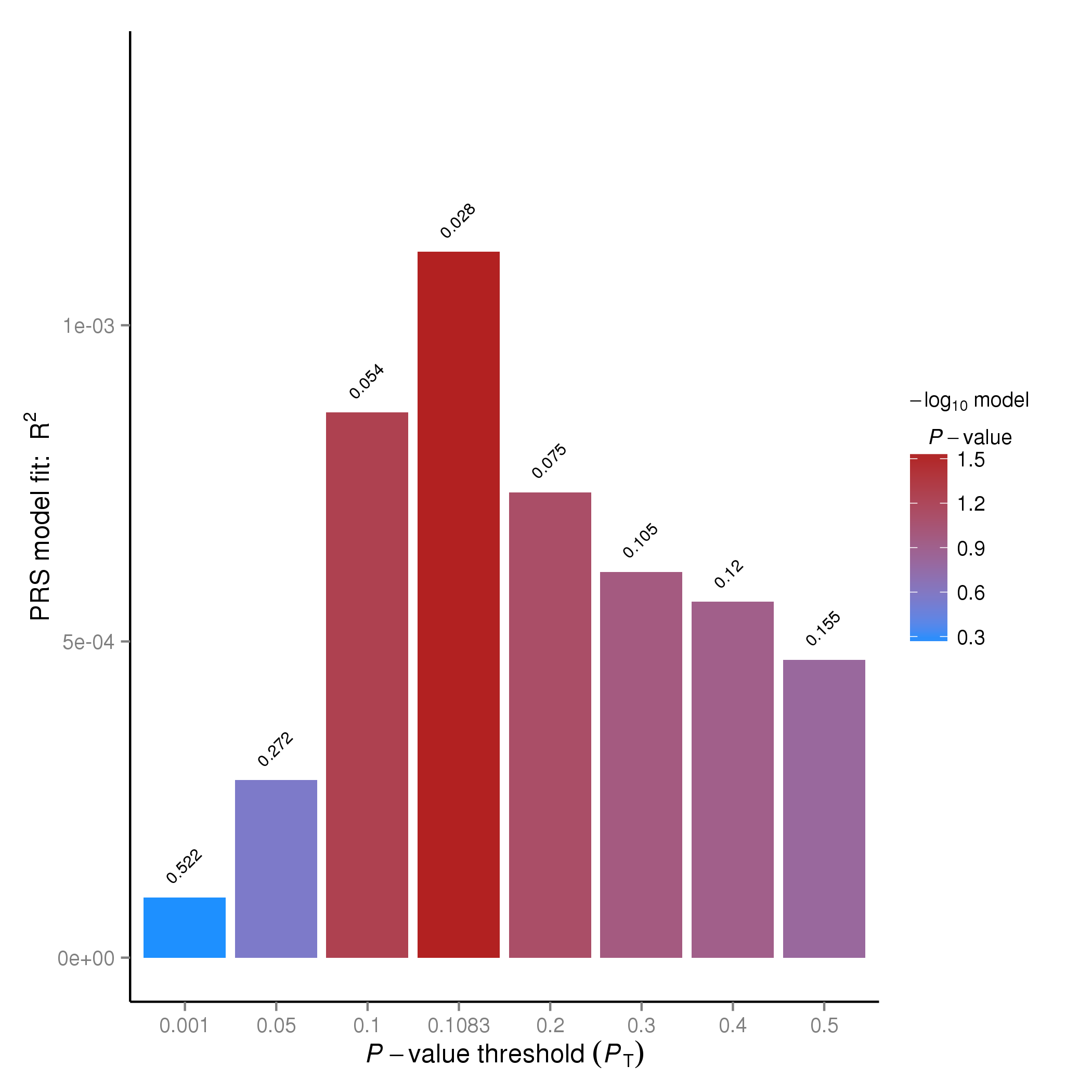


Supplementary Figure 6e: Bipolar Disorder PRS association with response to facial emotion as a proportion index

Supplementary Figure 7

Supplementary Figure 7: Association of Major Depressive Disorder PRS across seven thresholds
(Pt = 0.01, 0.05, 0.1, 0.2, 0.3, 0.4, 0.5) and the optimal threshold.


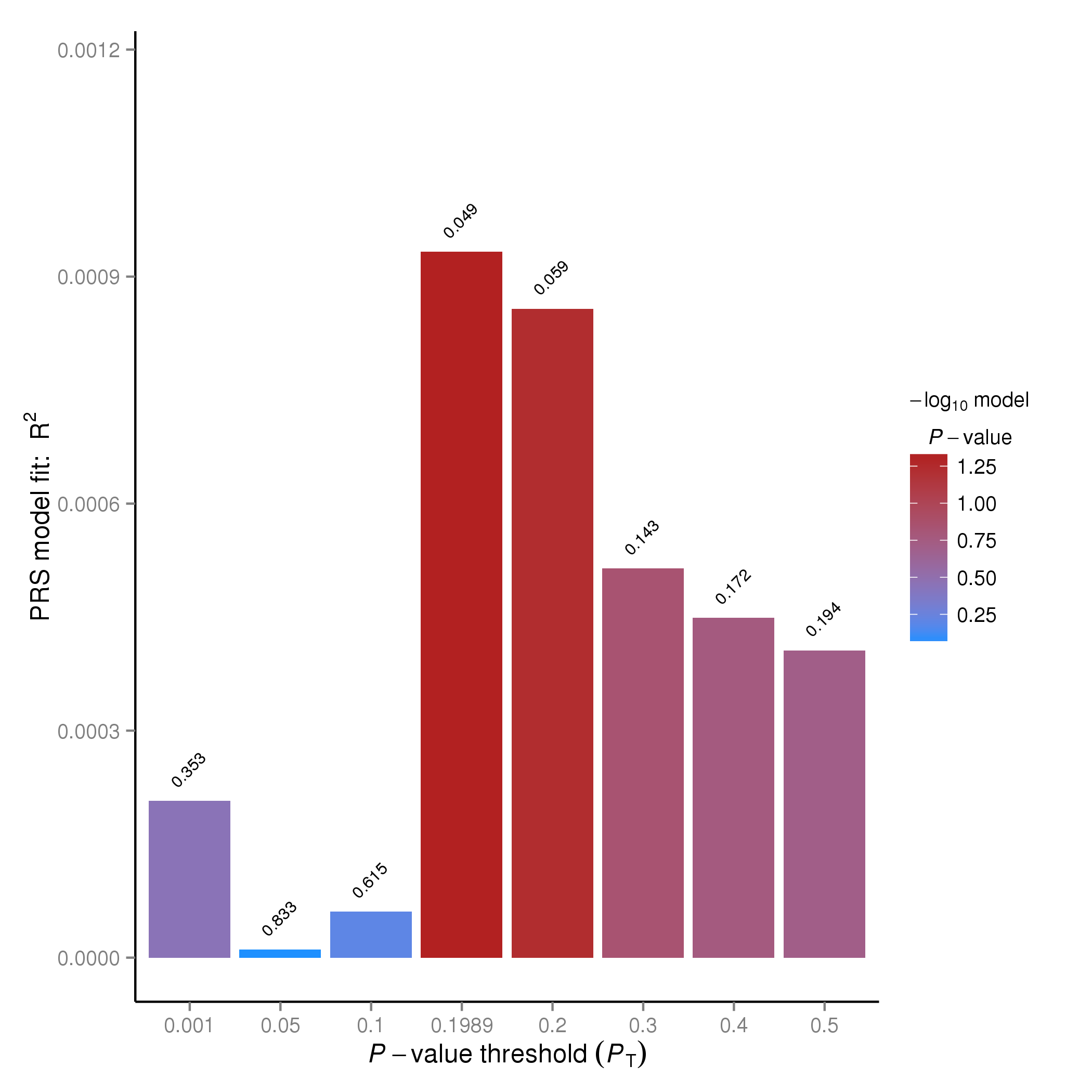


Supplementary Figure 7a: Major Depressive Disorder PRS association with response to happy faces


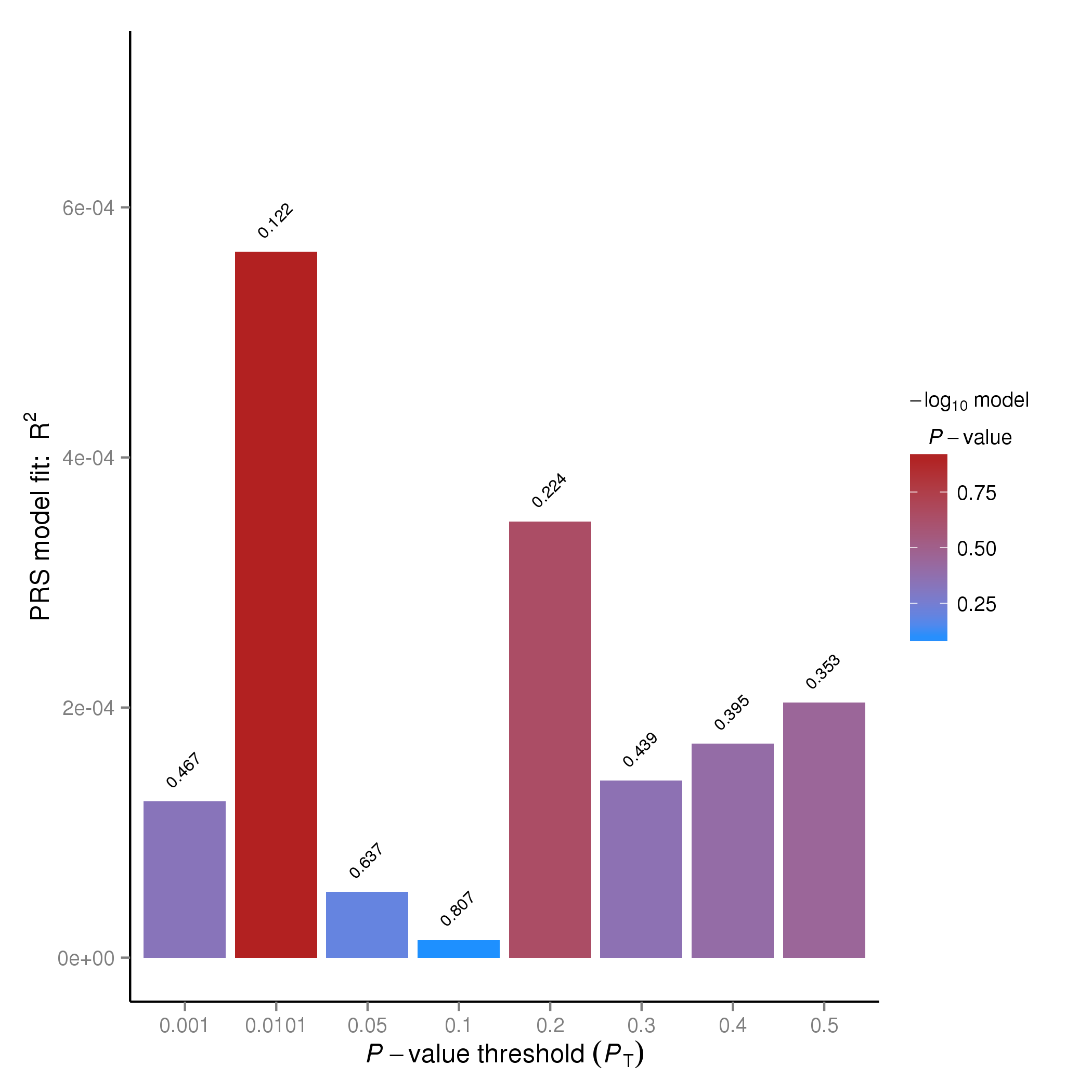


Supplementary Figure 7b: Major Depressive Disorder PRS association with response to sad faces


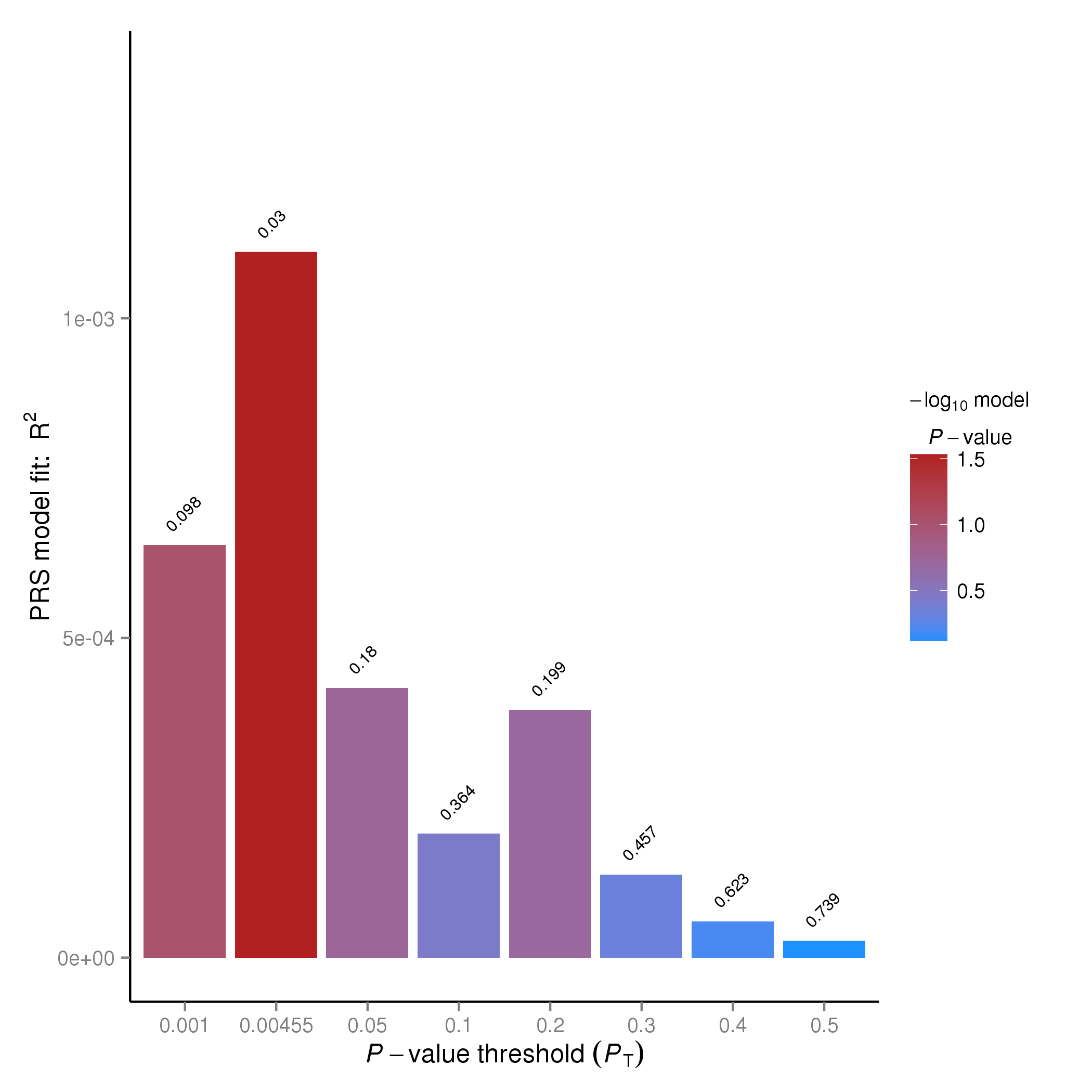


Supplementary Figure 7c: Major Depressive Disorder PRS association with response to angry faces


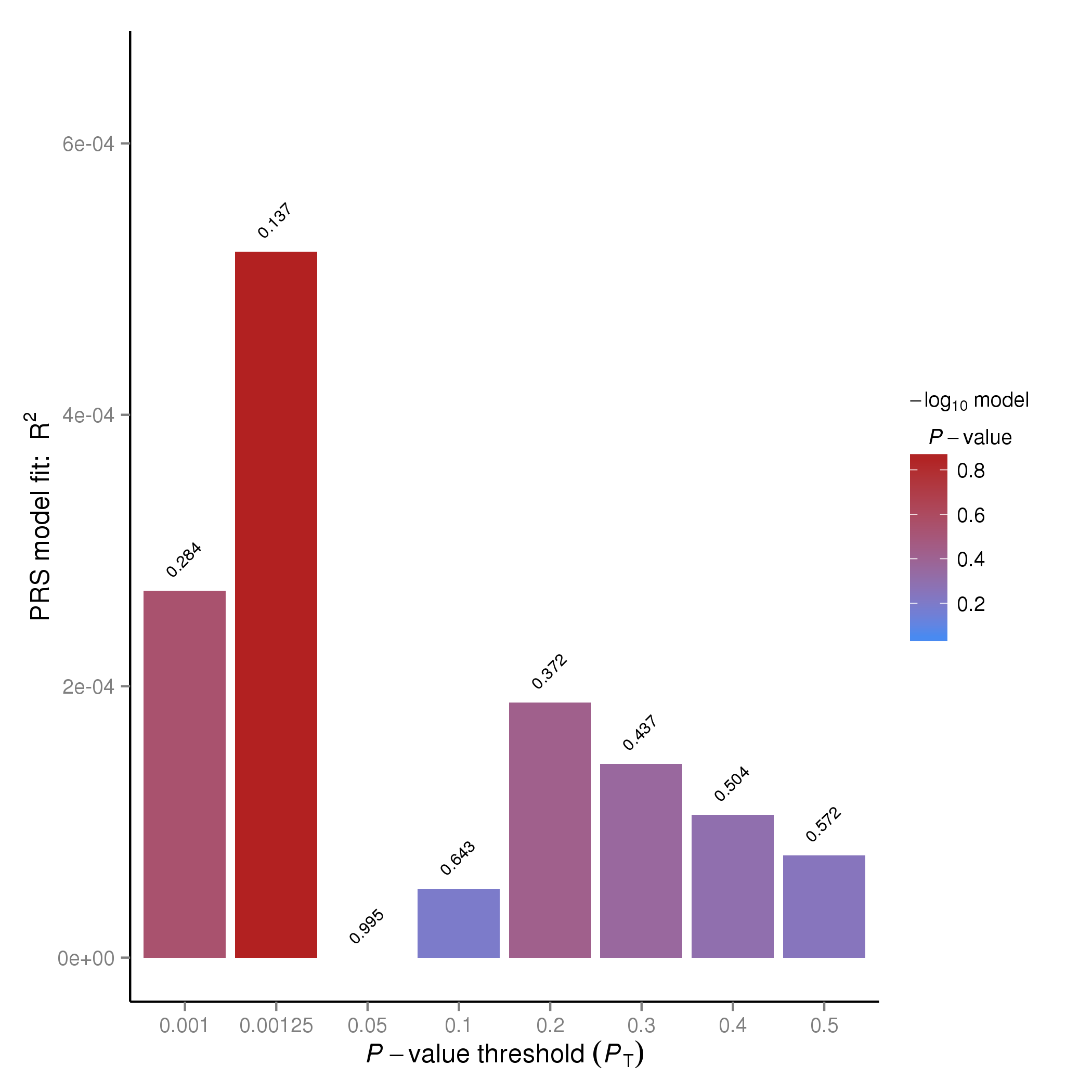


Supplementary Figure 7d: Major Depressive Disorder PRS association with response to fearful faces


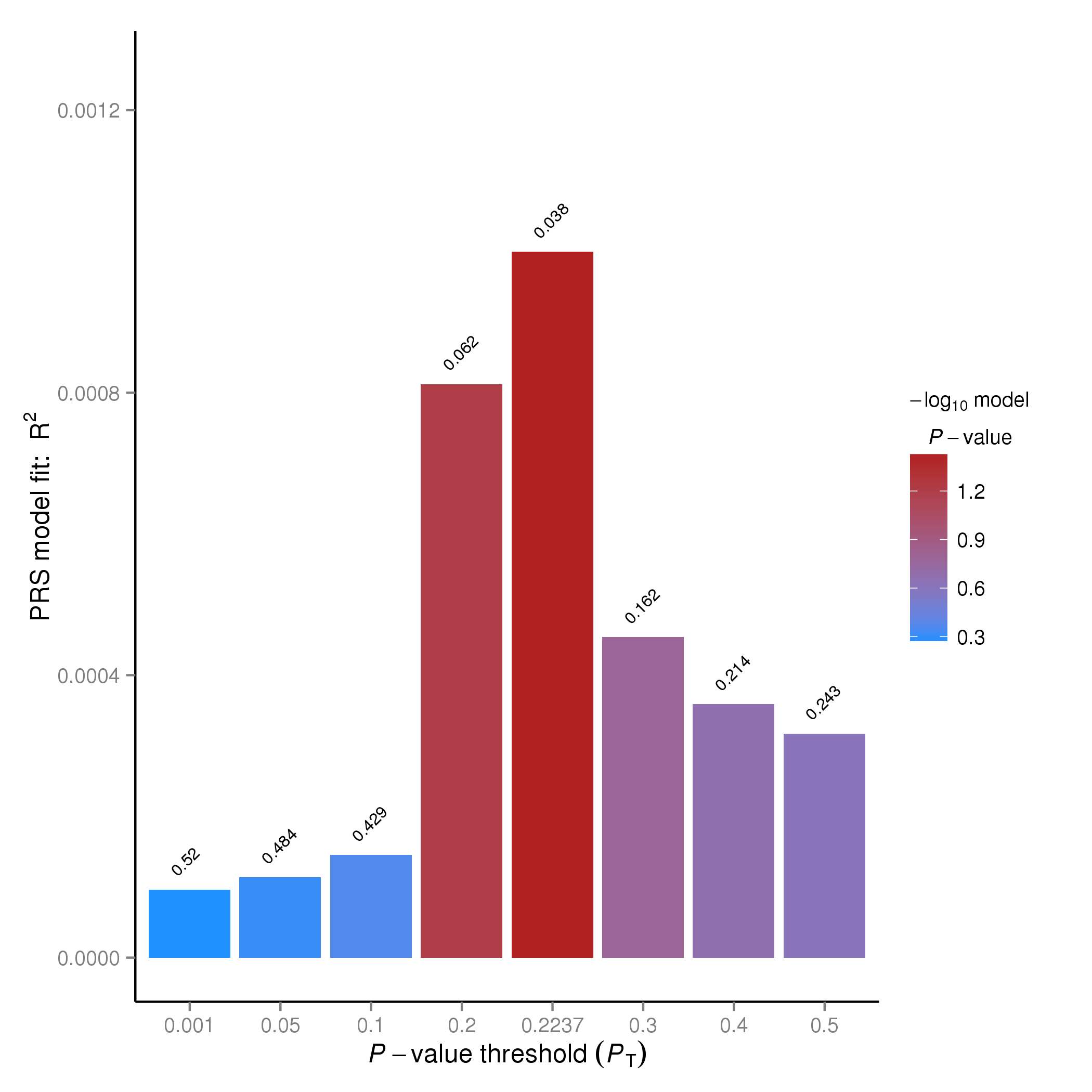


Supplementary Figure 7e: Major Depressive Disorder PRS association with response to facial emotion as a proportion index

Supplementary Figure 8

Supplementary Figure 8: Association of Autism Spectrum Disorder PRS across seven thresholds
(Pt = 0.01, 0.05, 0.1, 0.2, 0.3, 0.4, 0.5) and the optimal threshold.


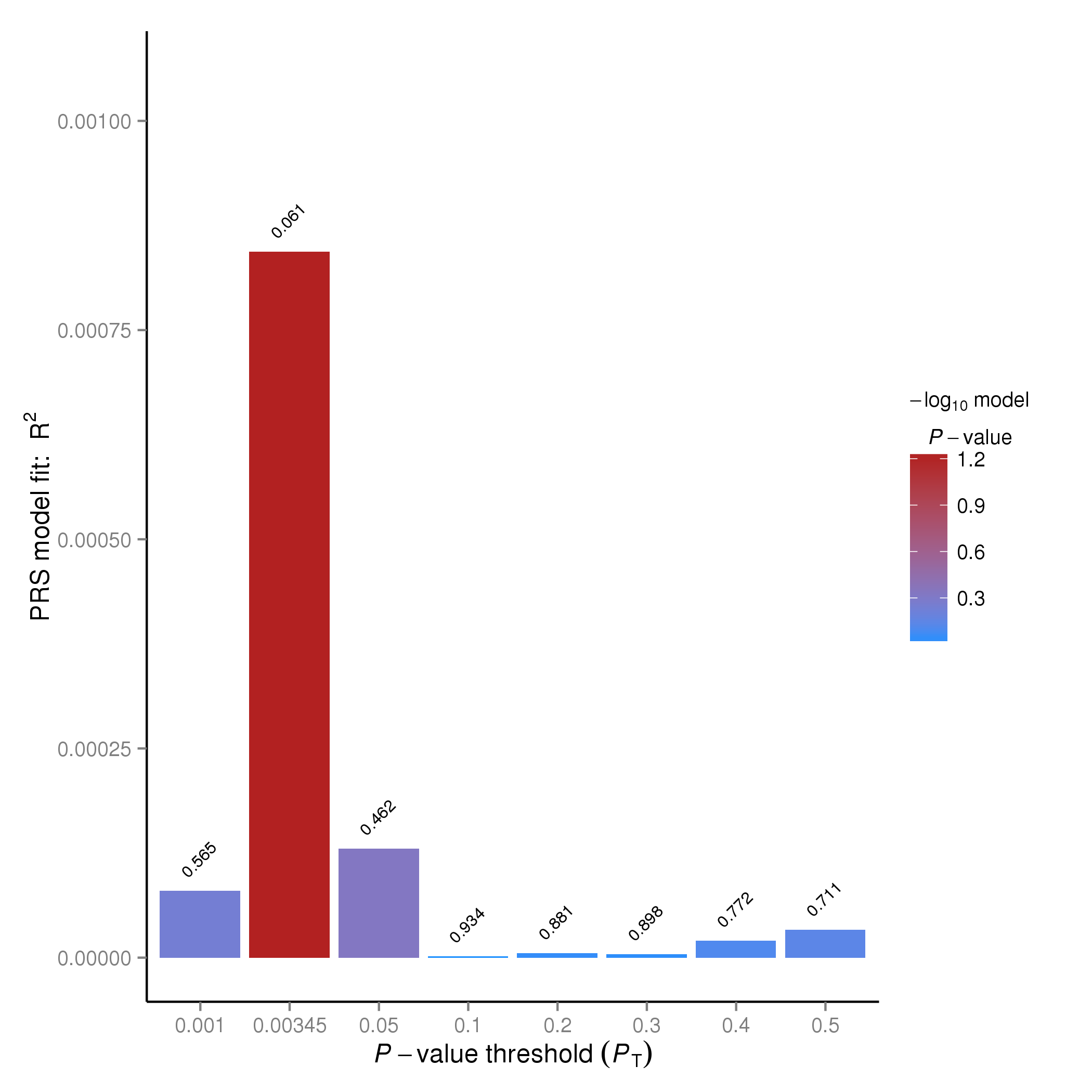


Supplementary Figure 8a: Autism Spectrum Disorder PRS association with response to happy faces


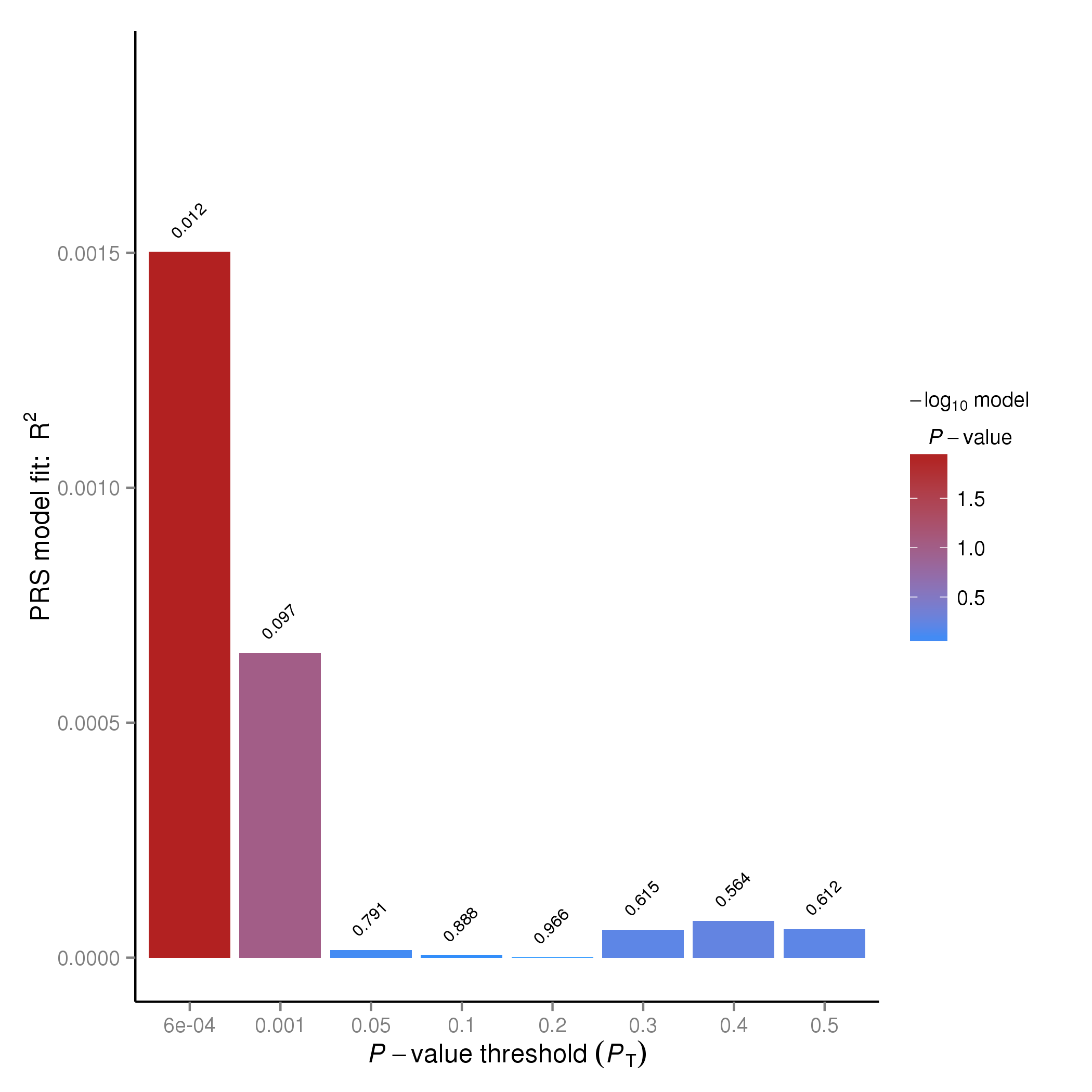


Supplementary Figure 8b: Autism Spectrum Disorder PRS association with response to sad faces


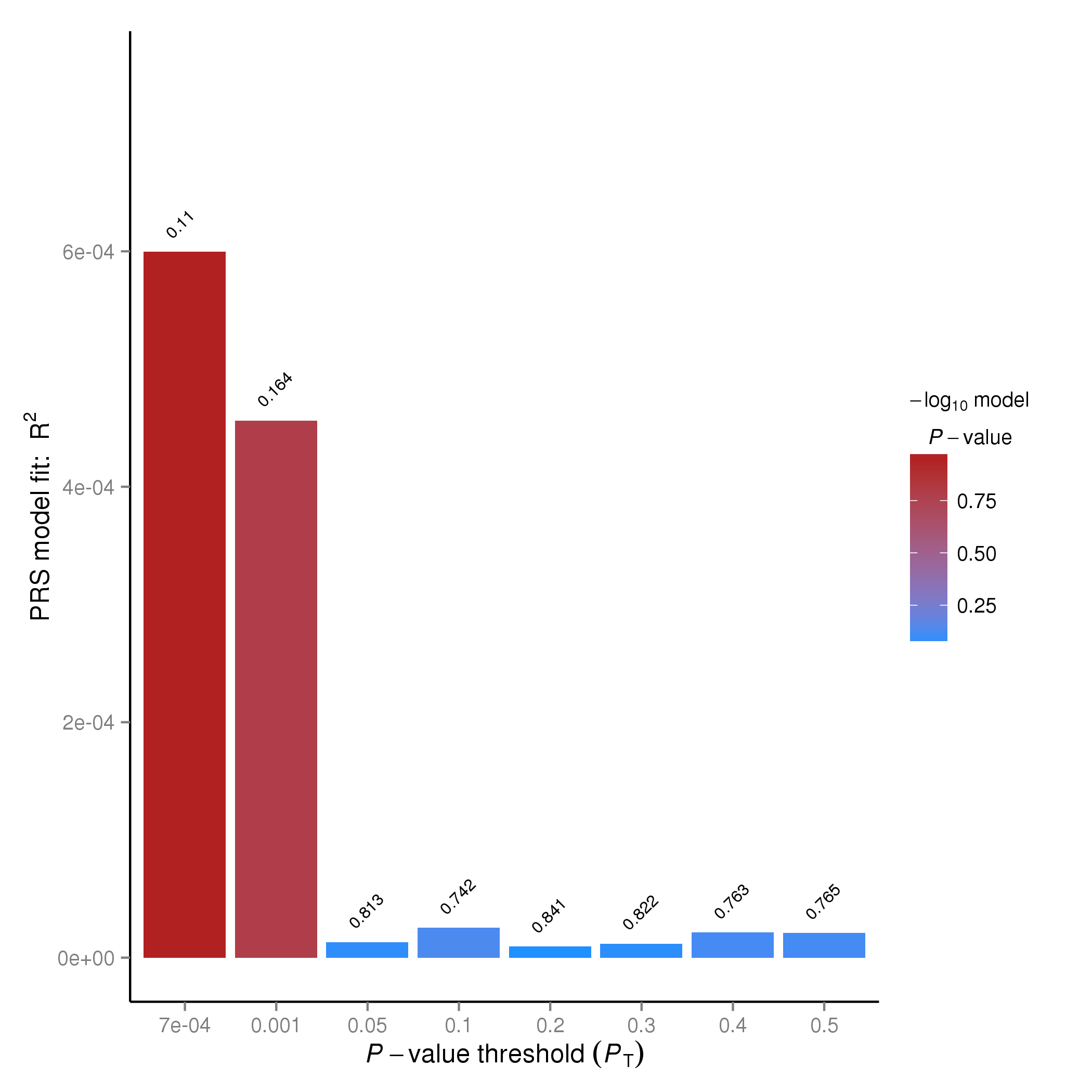


Supplementary Figure 8c: Autism Spectrum Disorder PRS association with response to angry faces


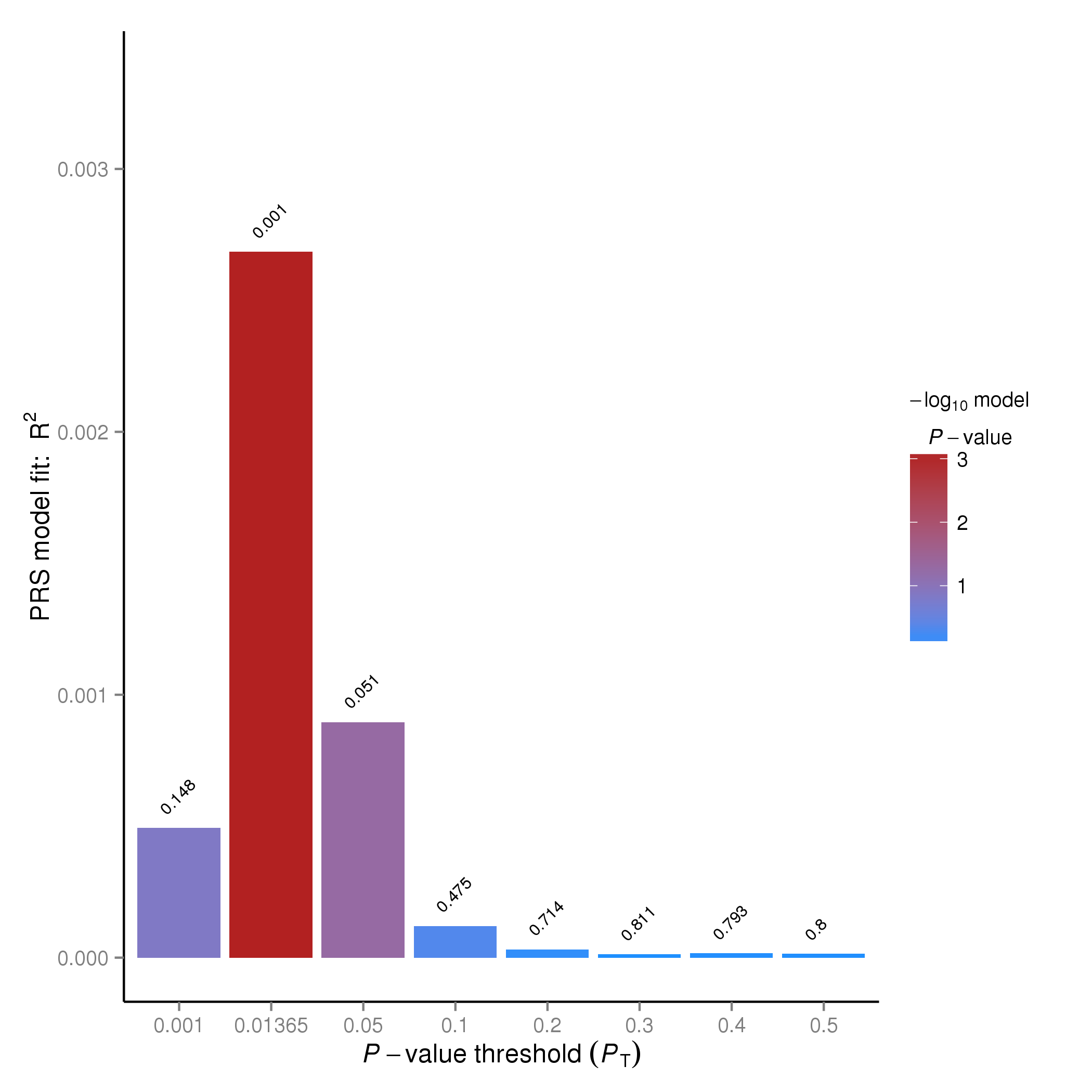


Supplementary Figure 8d: Autism Spectrum Disorder PRS association with response to fearful faces


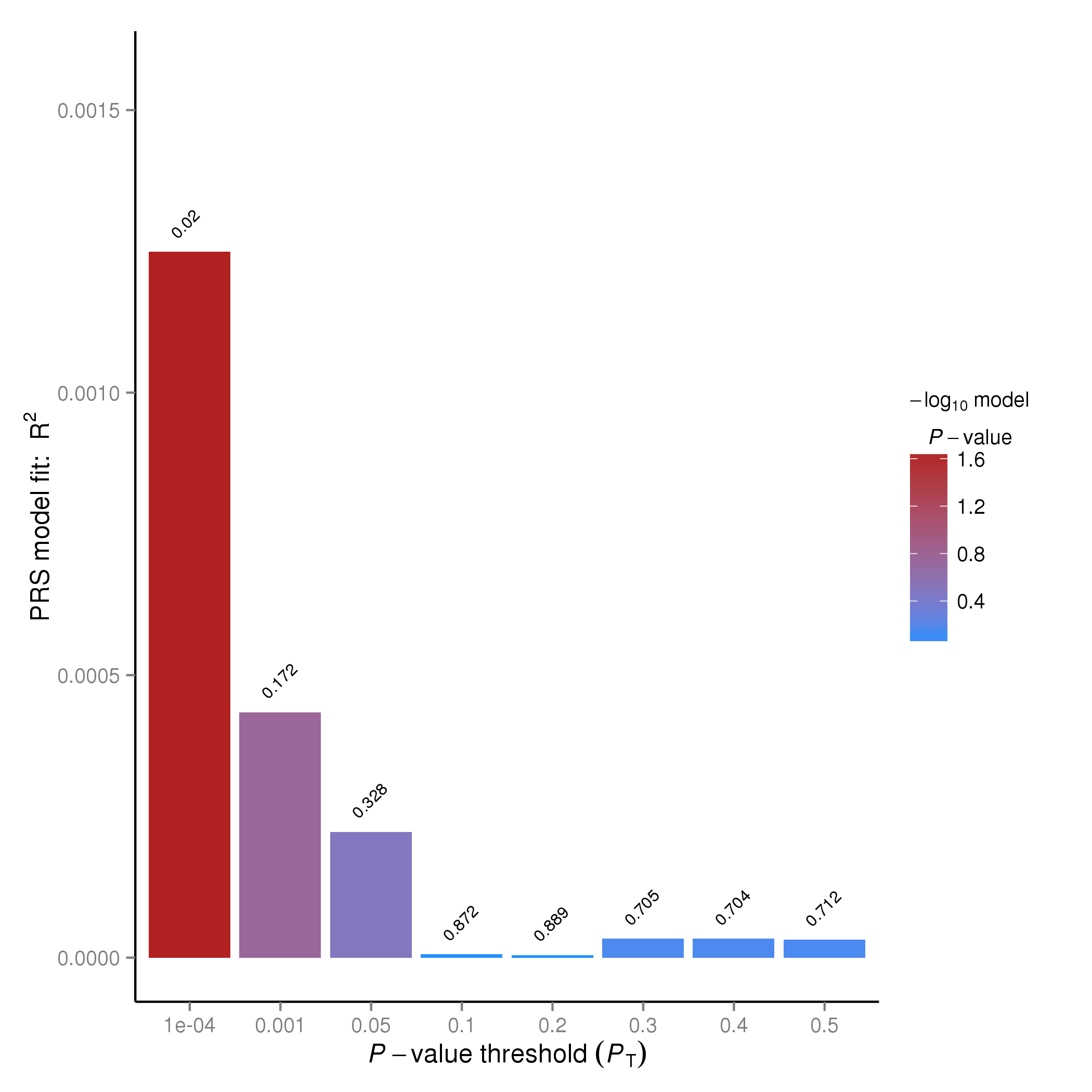


Supplementary Figure 8e: Autism Spectrum Disorder PRS association with response to facial emotion as a proportion index

Supplementary Figure 9

Supplementary Figure 9: Association of Anorexia Nervosa PRS across seven thresholds
(Pt = 0.01, 0.05, 0.1, 0.2, 0.3, 0.4, 0.5) and the optimal threshold.


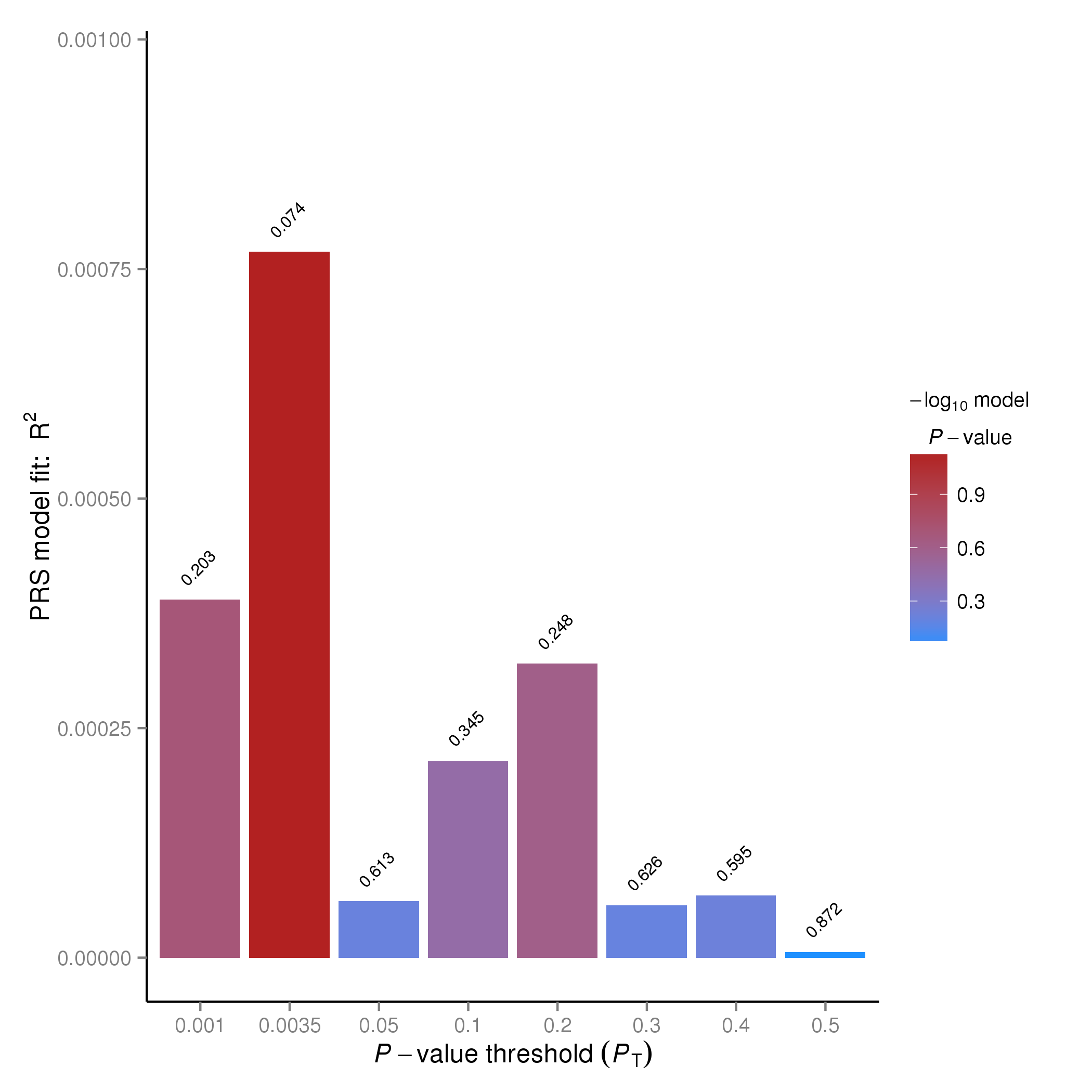


Supplementary Figure 9a: Anorexia Nervosa PRS association with response to happy faces


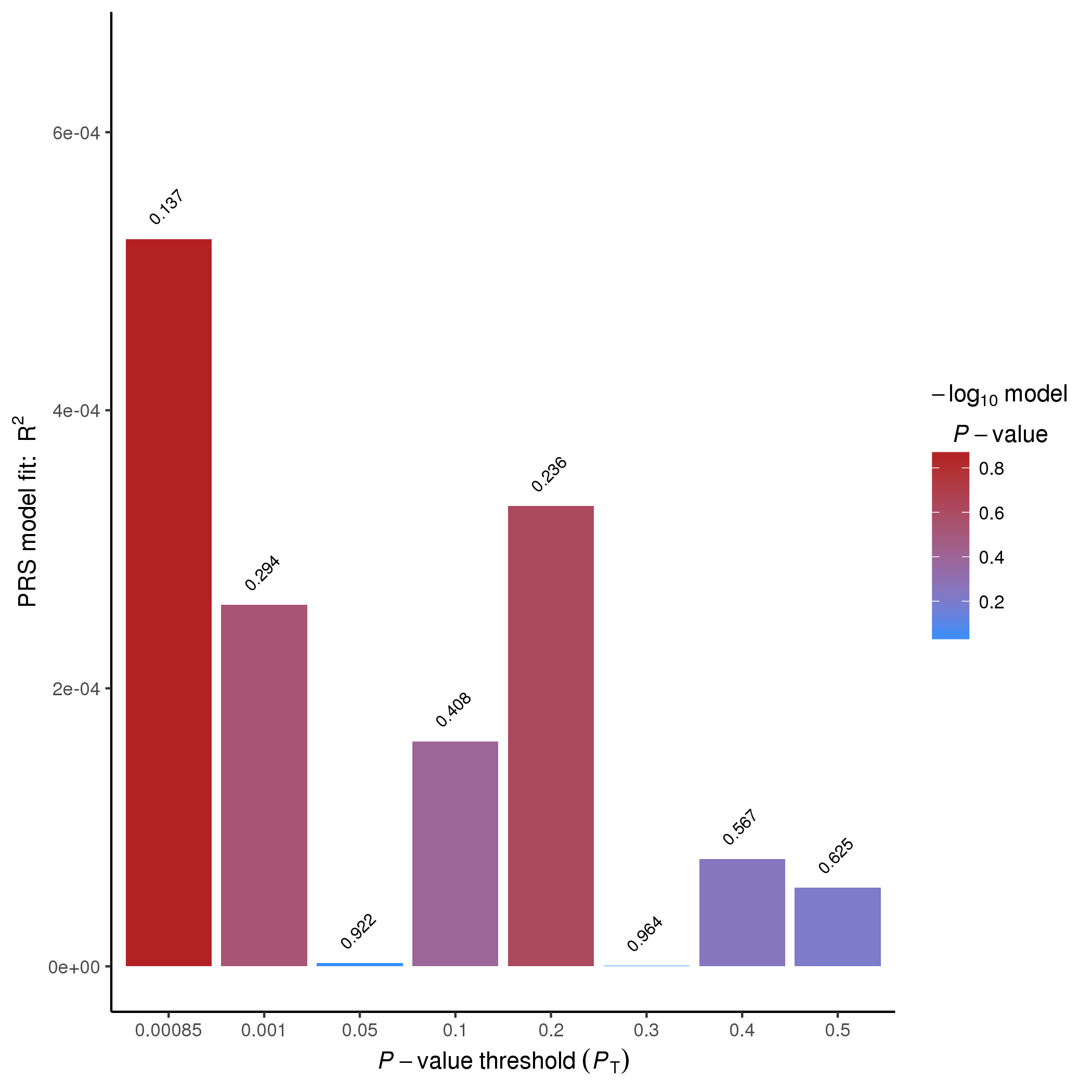


Supplementary Figure 9b: Anorexia Nervosa PRS association with response to sad faces


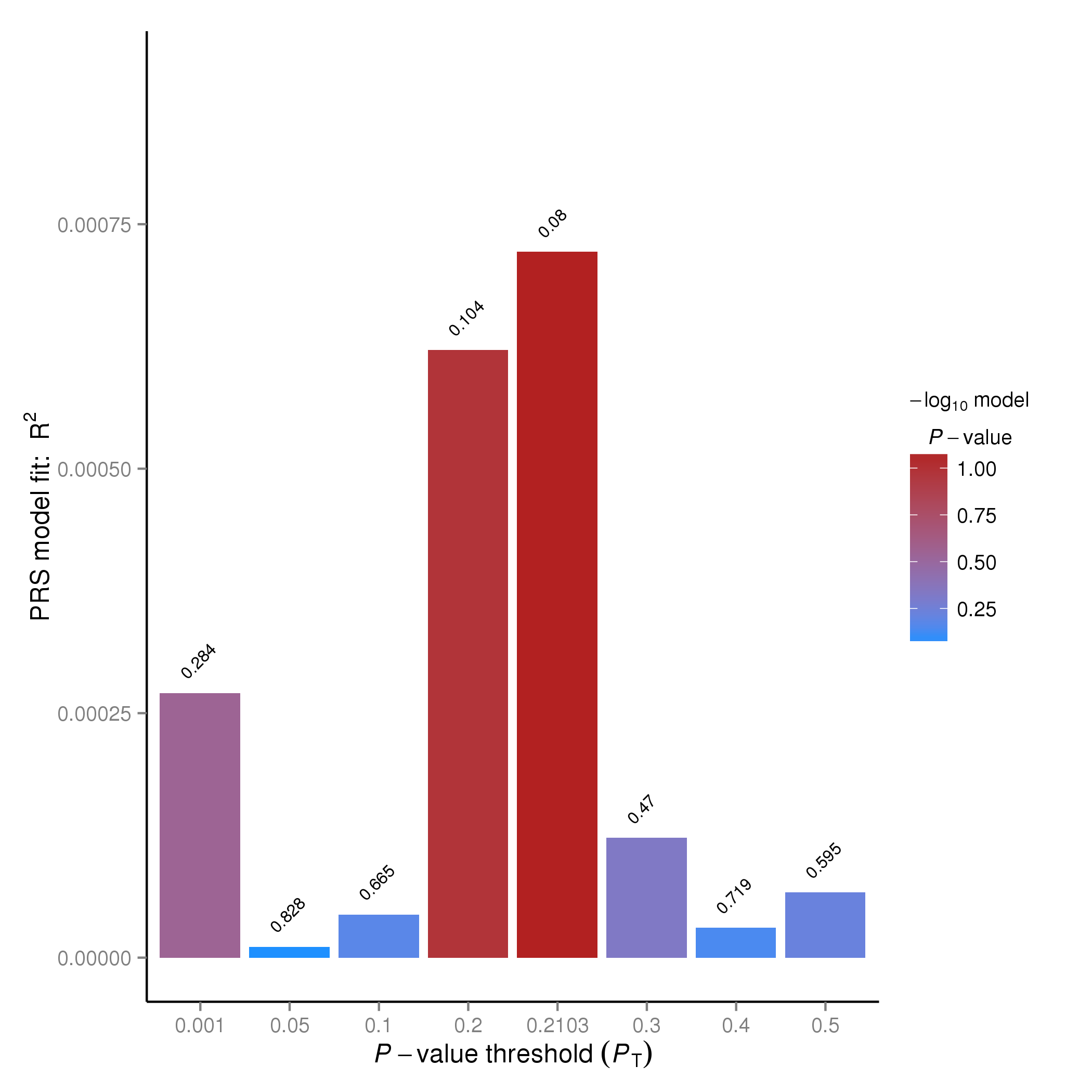


Supplementary Figure 9c: Anorexia Nervosa PRS association with response to angry faces


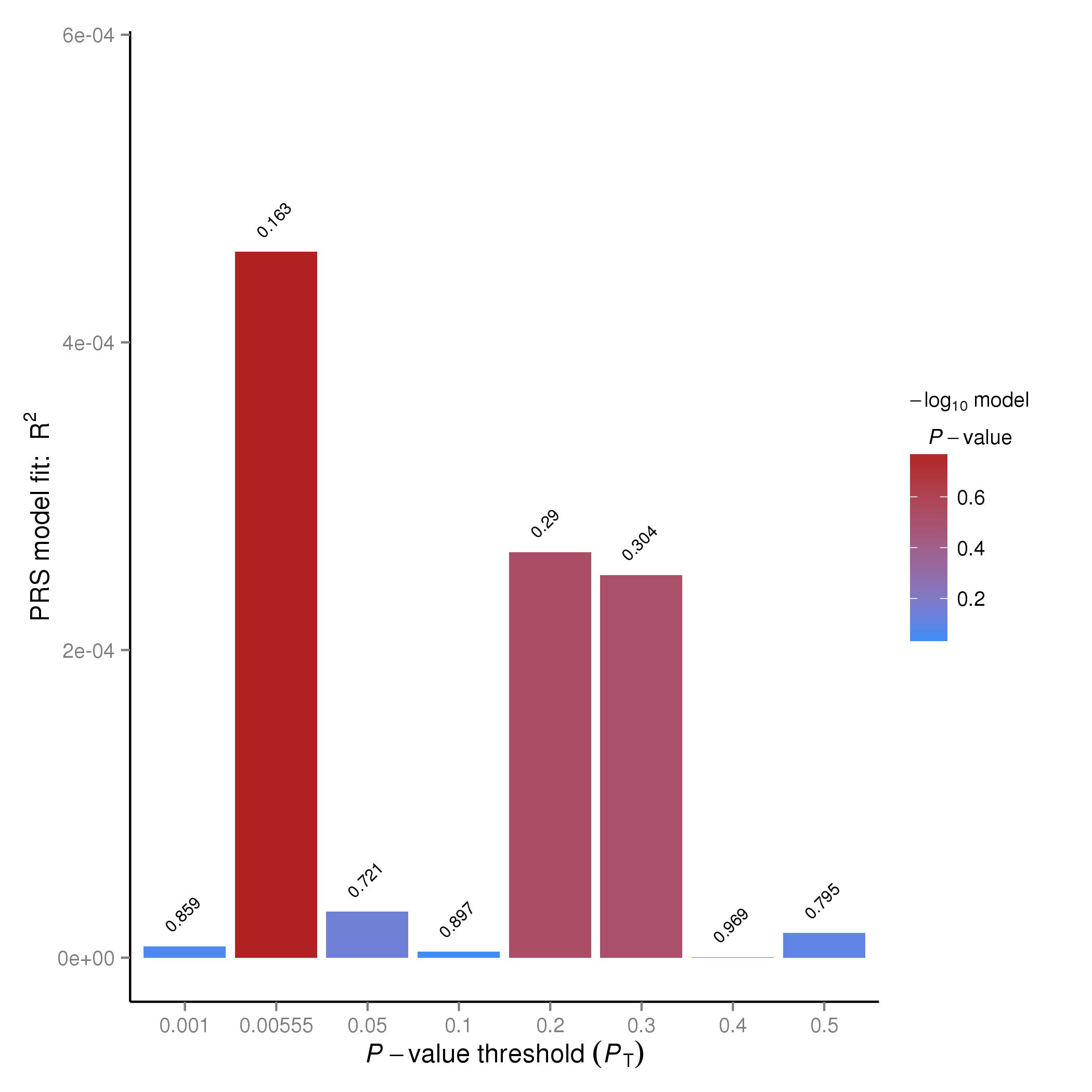


Supplementary Figure 9d: Anorexia Nervosa PRS association with response to fearful faces


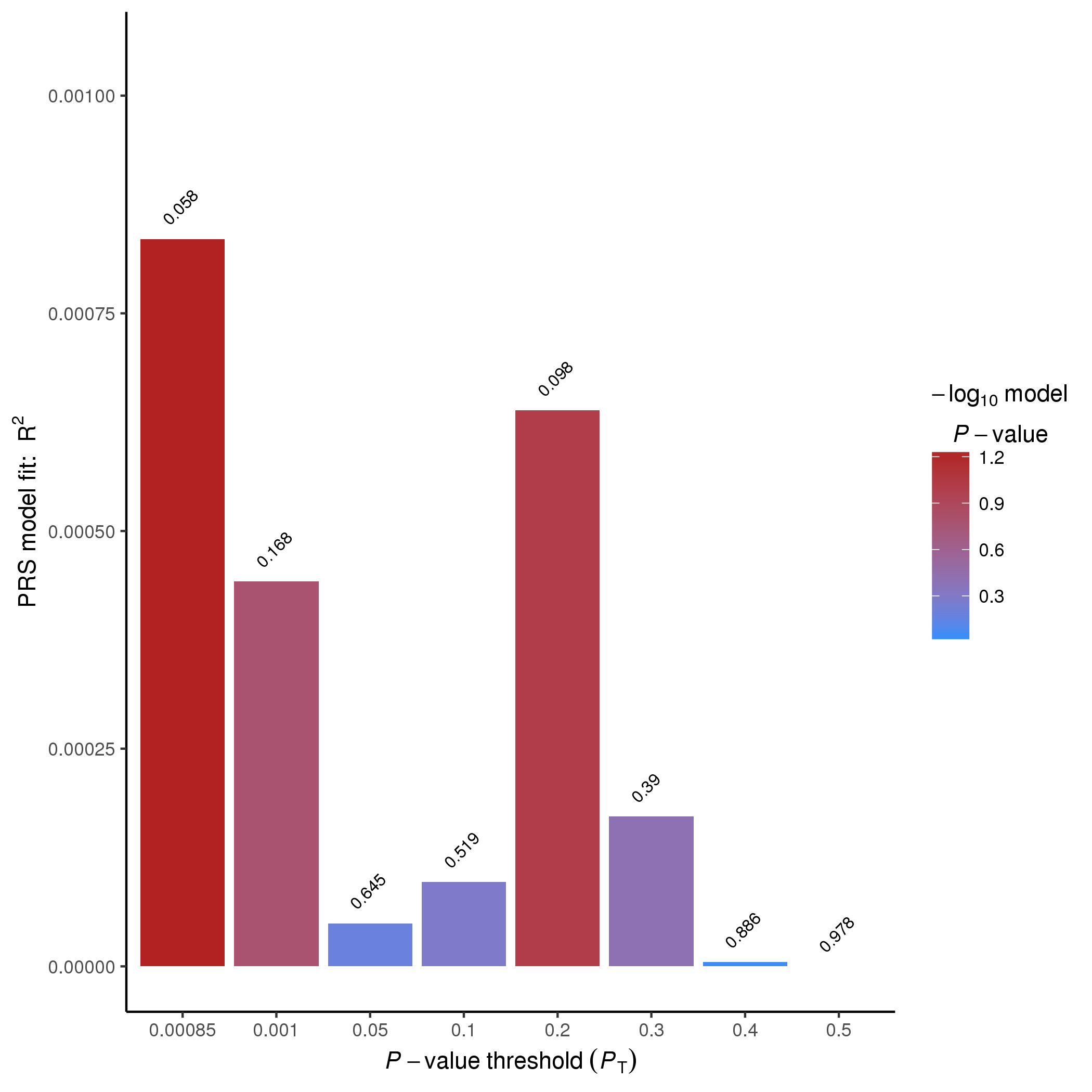


Supplementary Figure 9e: Anorexia Nervosa PRS association with response to facial emotion as a proportion index

Supplementary Figure 10

Supplementary Figure 10: Association of Anxiety (Case-Control) PRS across seven thresholds
(Pt = 0.01, 0.05, 0.1, 0.2, 0.3, 0.4, 0.5) and the optimal threshold.


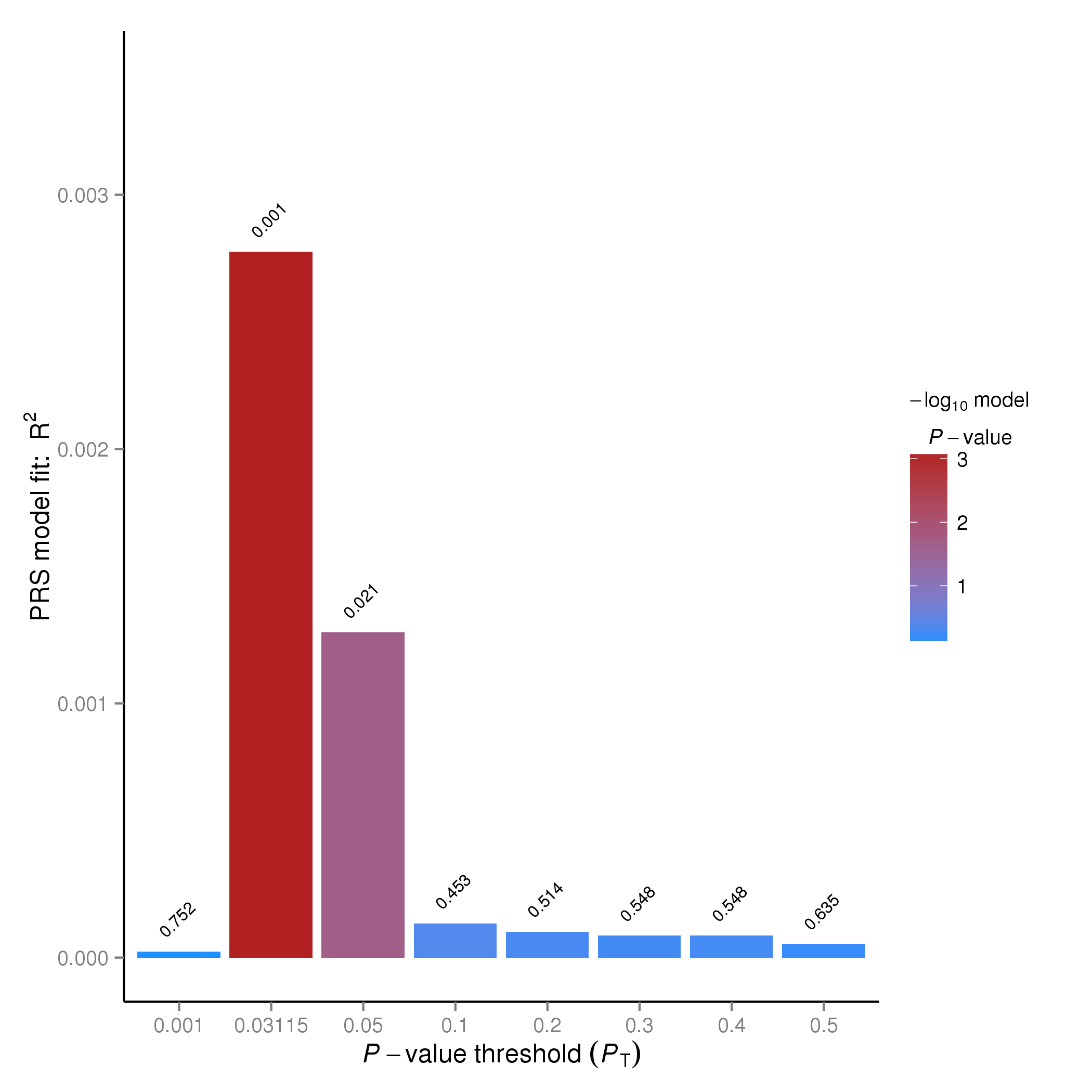


Supplementary Figure 10a: Anxiety (Case-Control) PRS association with response to happy faces


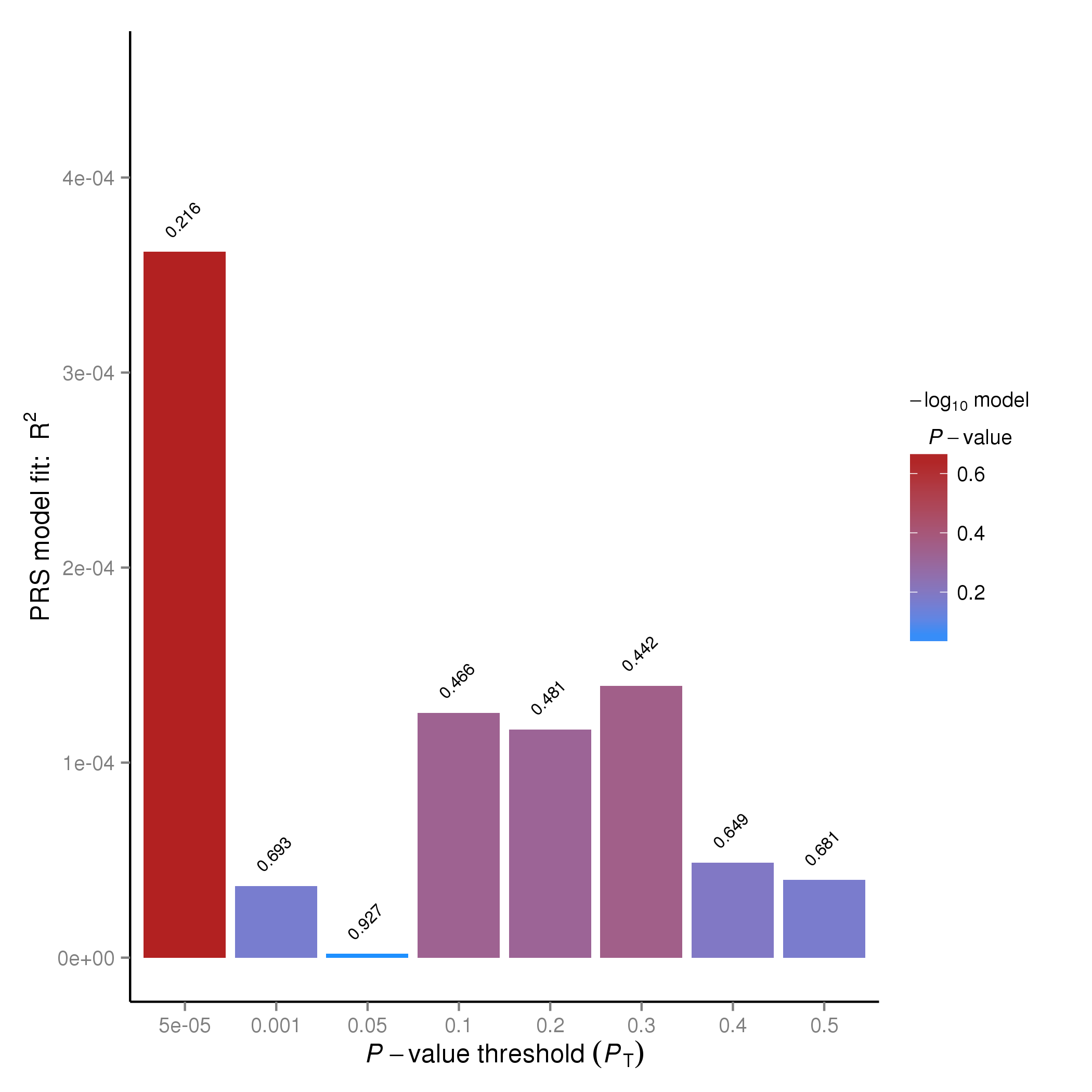


Supplementary Figure 10b: Anxiety (Case-Control) PRS association with response to sad faces


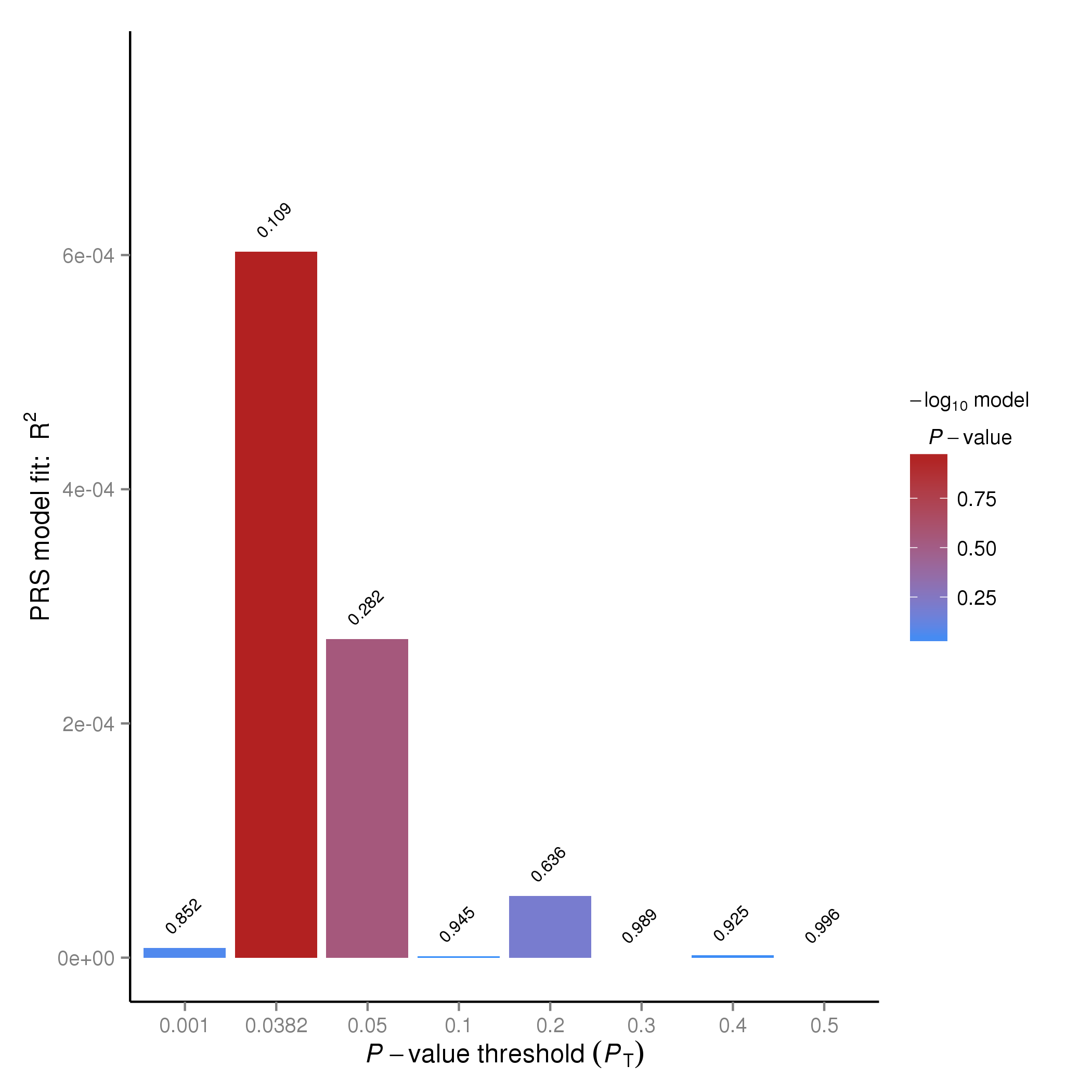


Supplementary Figure 10c: Anxiety (Case-Control) PRS association with response to angry faces


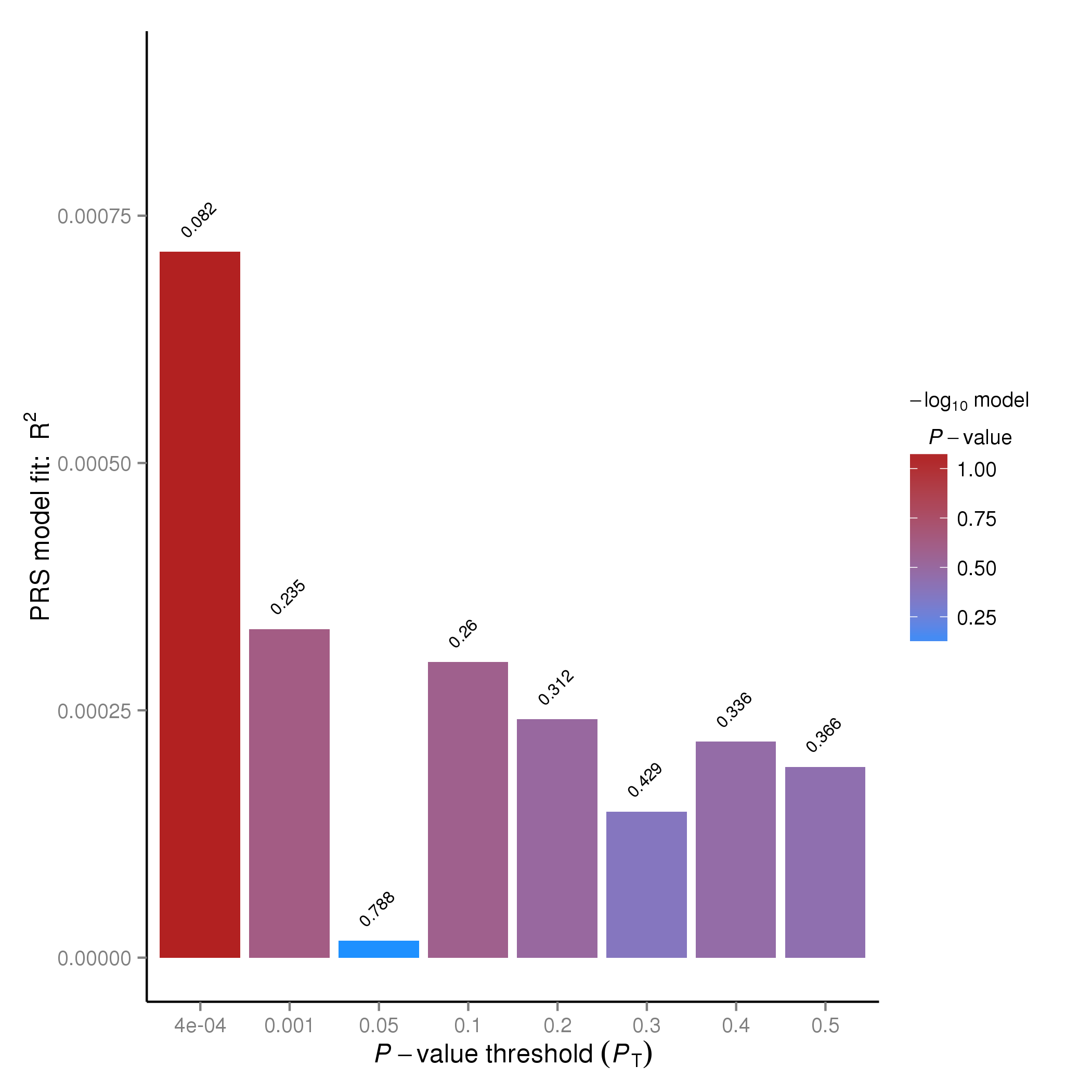


Supplementary Figure 10d: Anxiety (Case-Control) PRS association with response to fearful faces


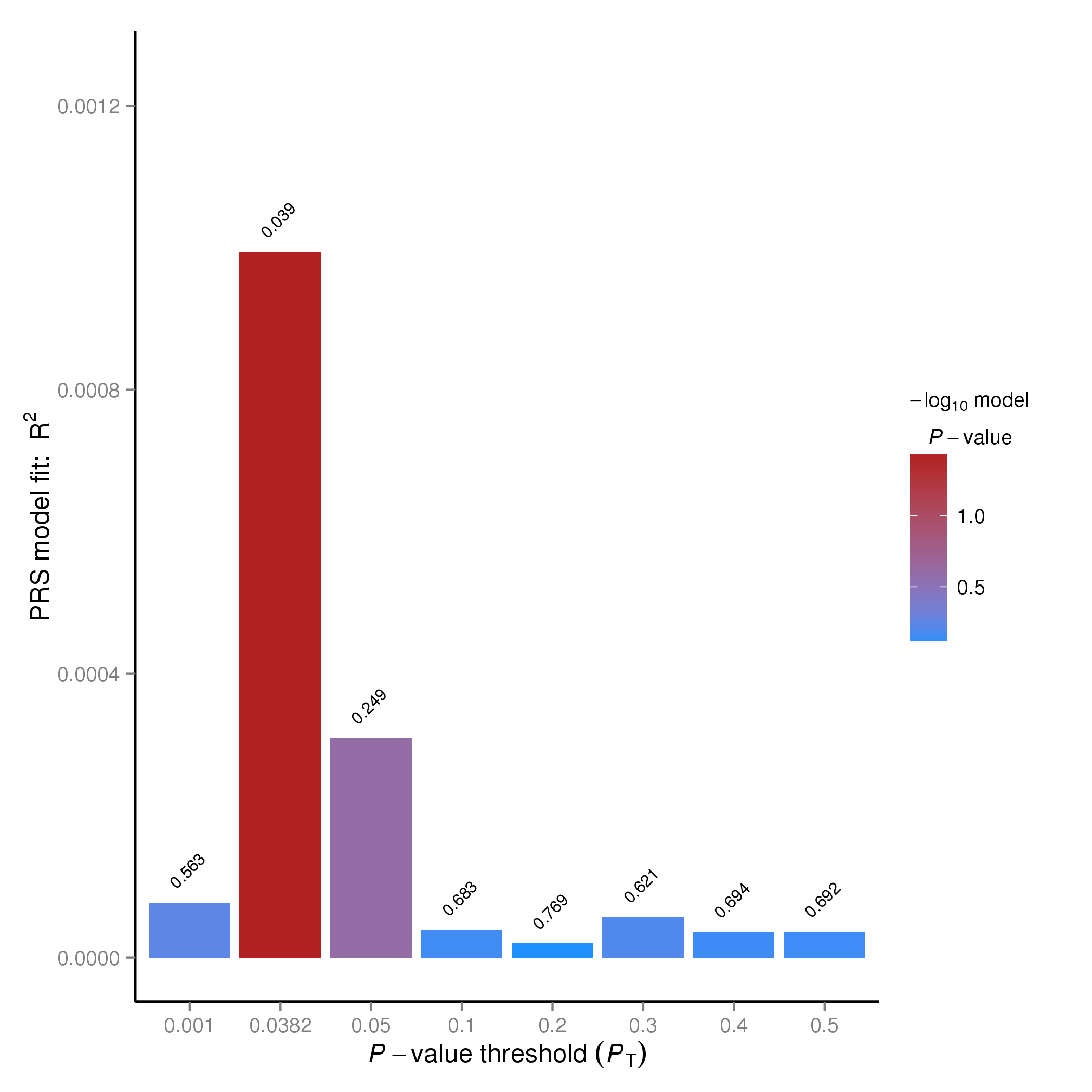


Supplementary Figure 10e: Anxiety (Case-Control) PRS association with response to facial emotion as a proportion index

Supplementary Figure 11

Supplementary Figure 11: Association of Anxiety (Factor Score) PRS across seven thresholds
(Pt = 0.01, 0.05, 0.1, 0.2, 0.3, 0.4, 0.5) and the optimal threshold.


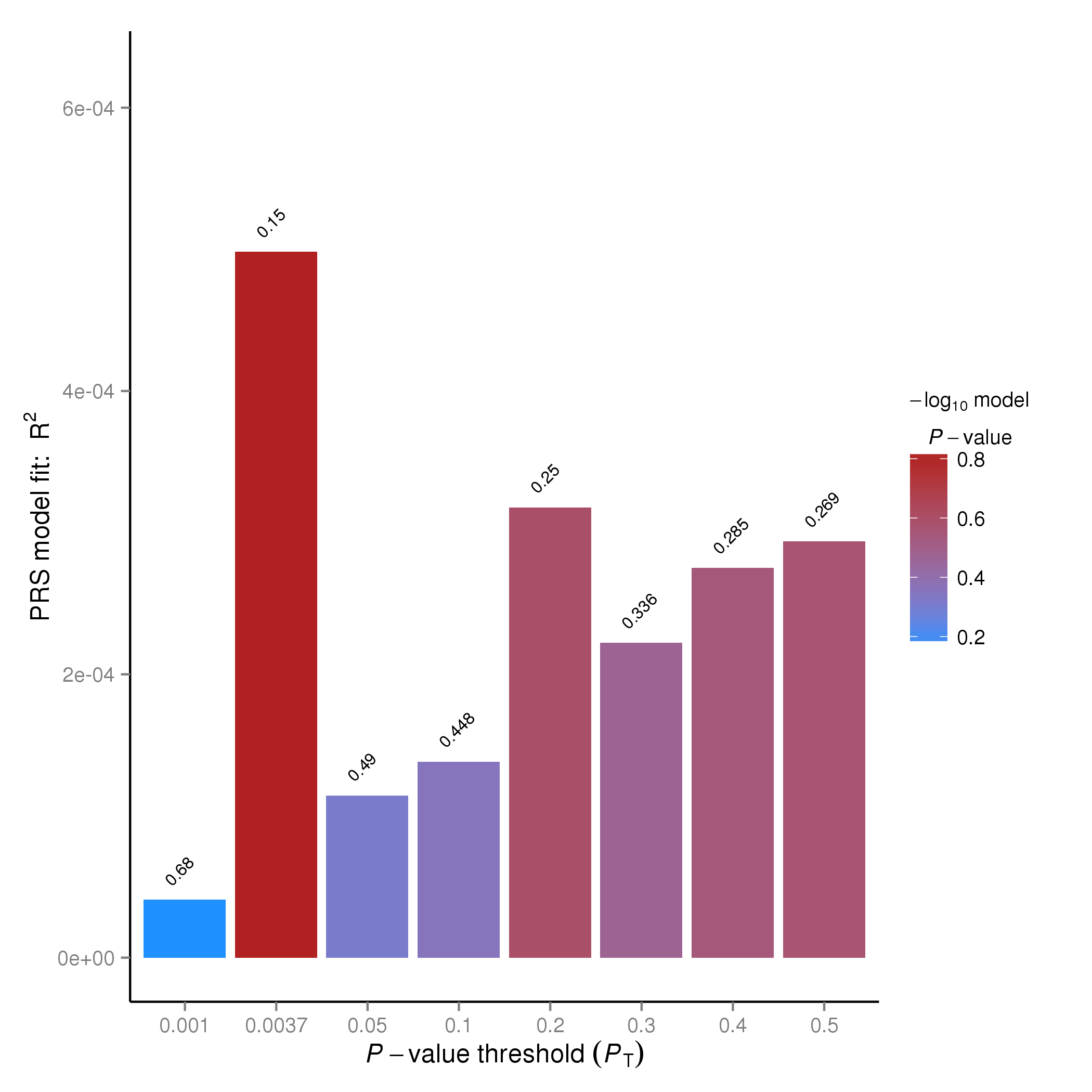


Supplementary Figure 11a: Anxiety (Factor Score) PRS association with response to happy faces


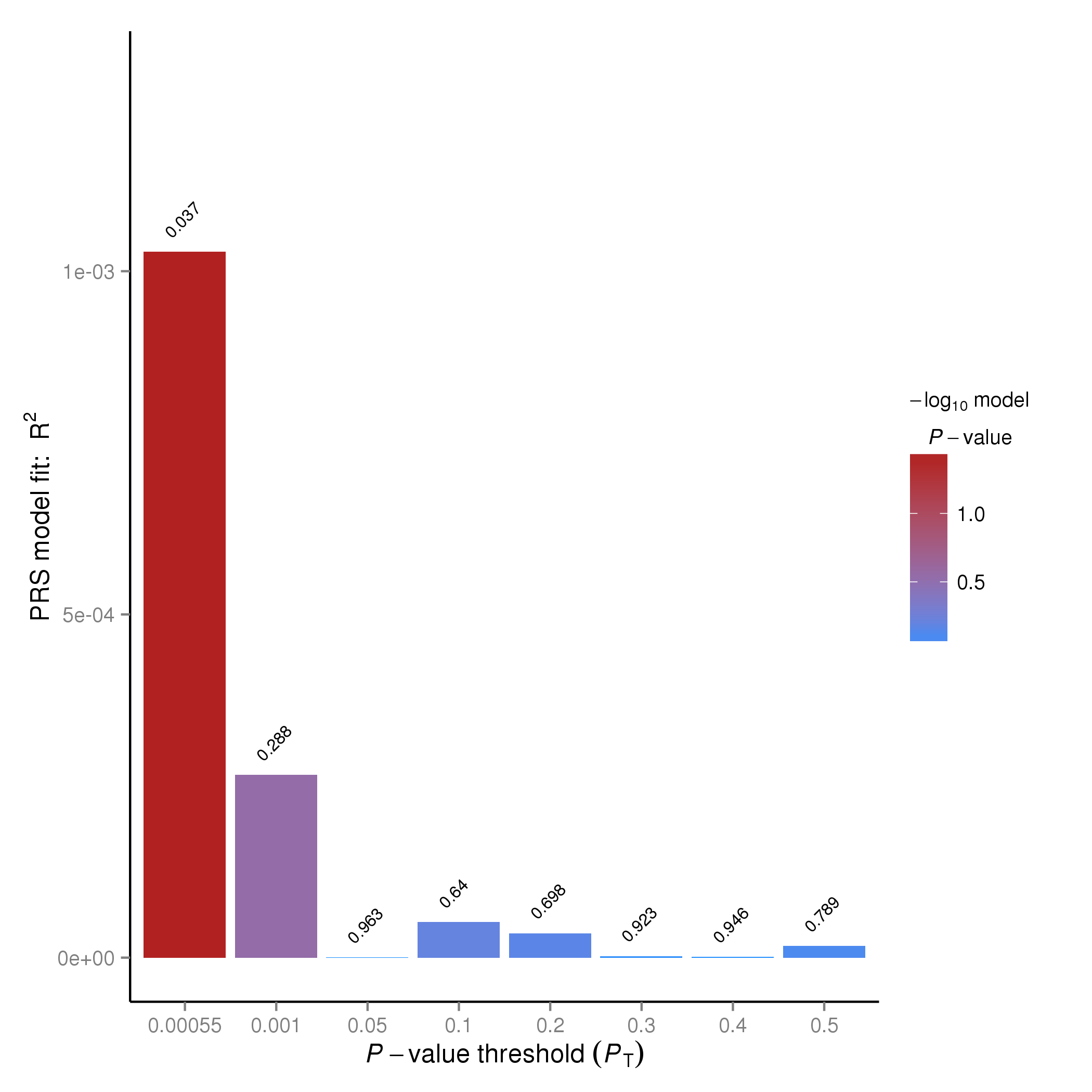


Supplementary Figure 11b: Anxiety (Factor Score) PRS association with response to sad faces


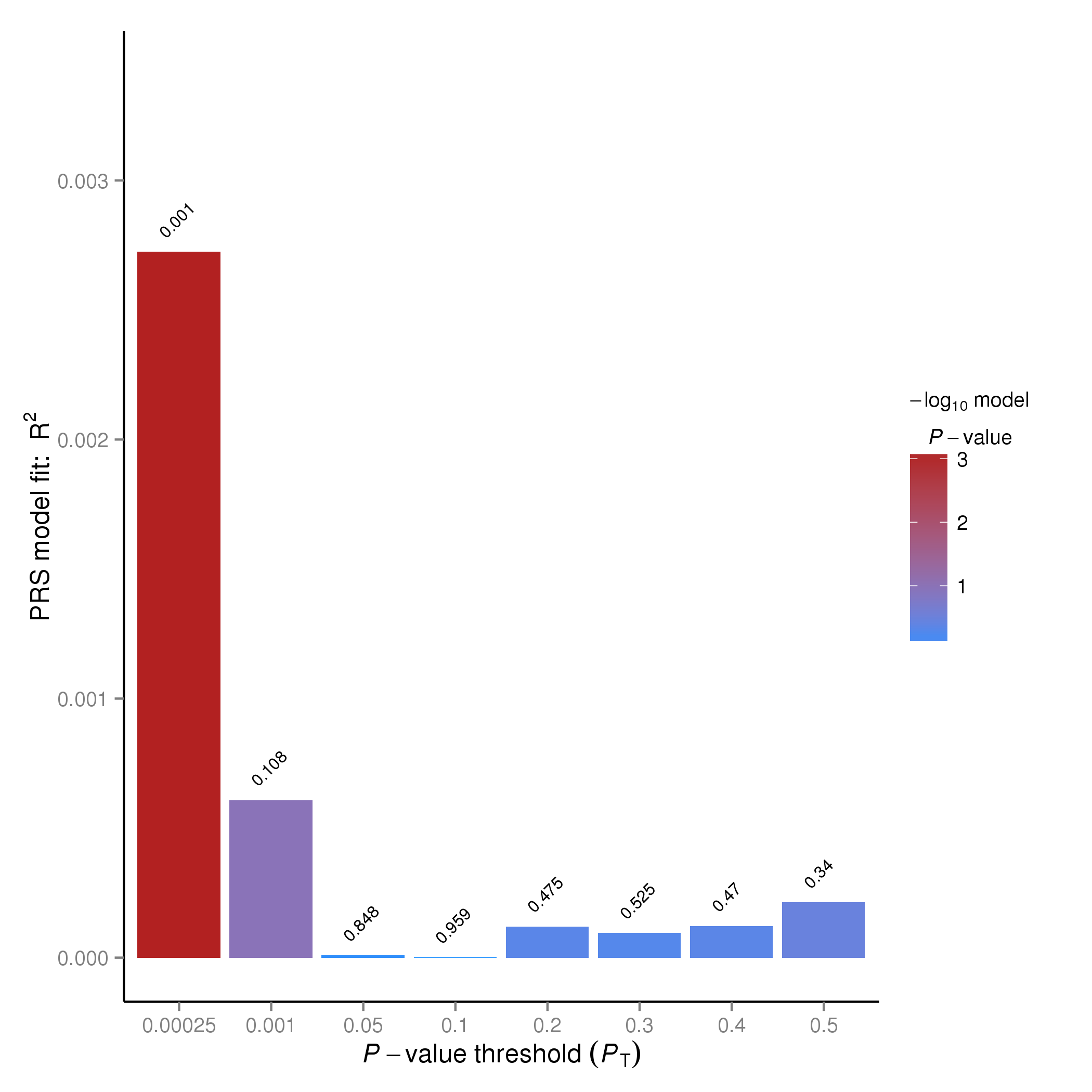


Supplementary Figure 11c: Anxiety (Factor Score) PRS association with response to angry faces


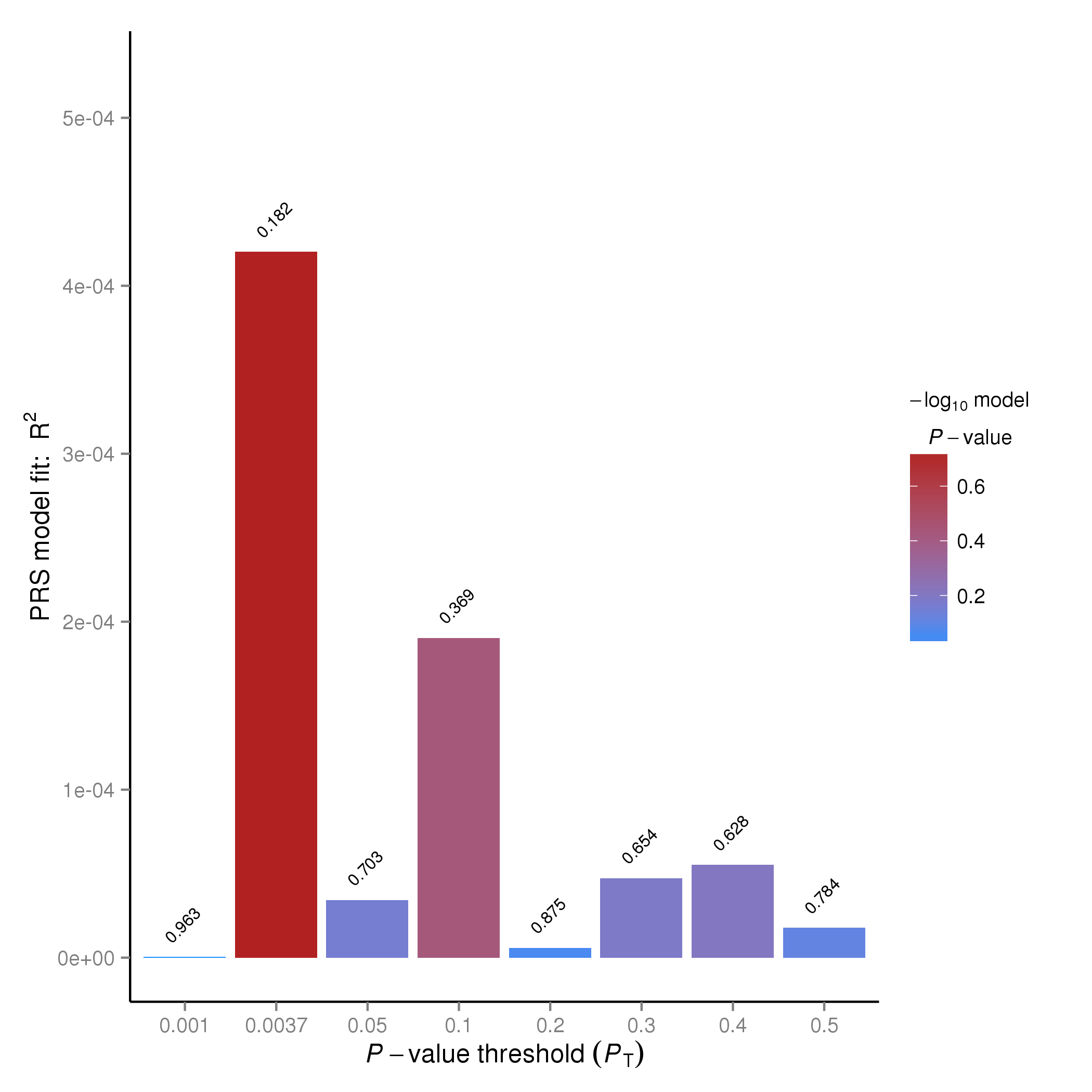


Supplementary Figure 11d: Anxiety (Factor Score) PRS association with response to fearful faces


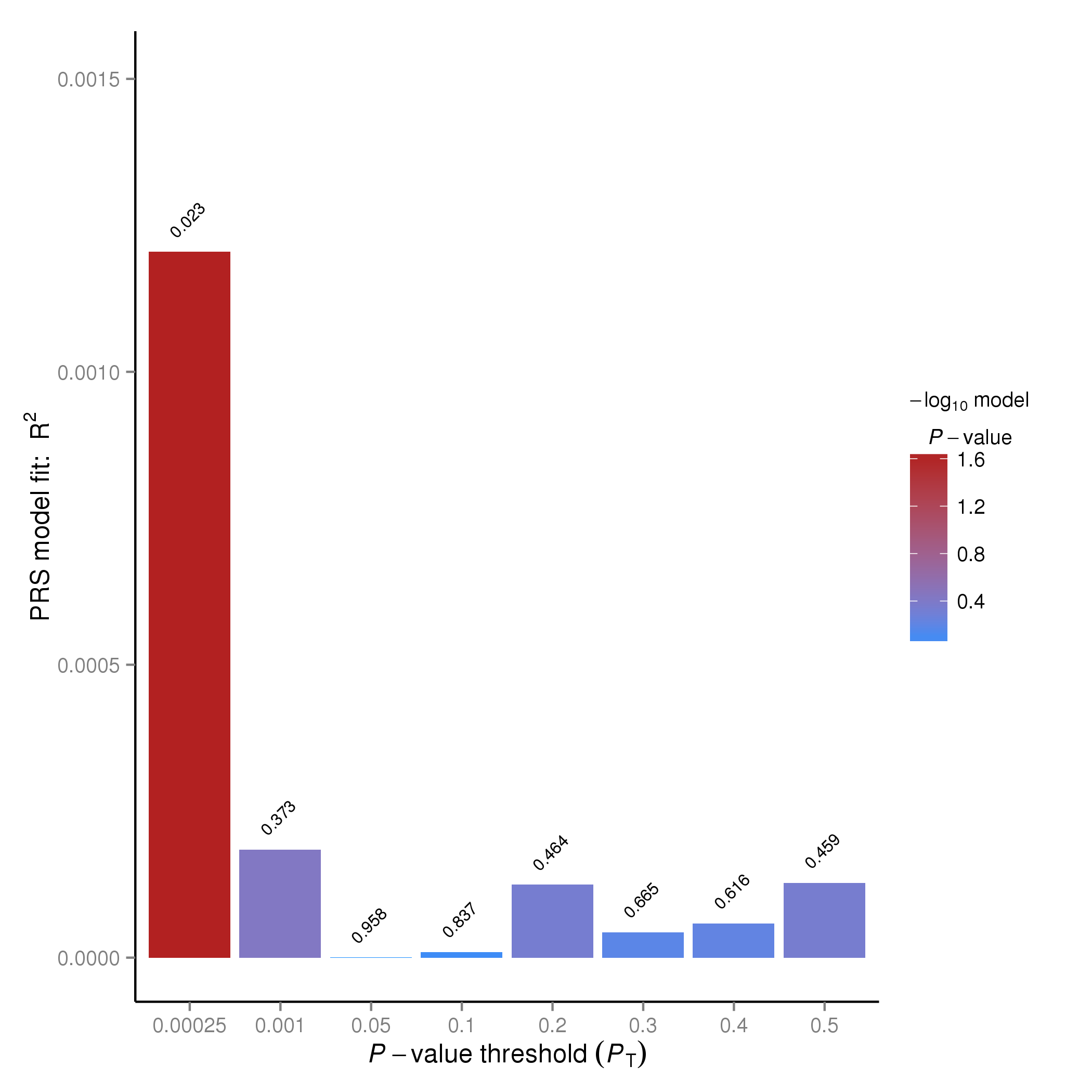


Supplementary Figure 11e: Anxiety (Factor Score) PRS association with response to facial emotion as a proportion index
